# Supplementary material for: IQCN disruption causes fertilization failure and male infertility due to manchette assembly defect
Source: EMBO Mol Med. 2022 Nov 2;14(12):e16501. doi: 10.15252/emmm.202216501 (PMC9728048; doi:10.15252/emmm.202216501)
Supplement: Supplementary file 1 — Appendix [file EMMM-14-e16501-s005.pdf]

Appendix

IQCN disruption causes fertilization failure and male infertility due to manchette assembly defect

Jing Dai,[1,2,3,6] Qi Li,[5,6] Qinwei Zhou,[2,3] Shen Zhang, [2,3] Junru Chen,[2,3] Yize Wang,[1] Jing Guo,[2,3] Yifan Gu,[1,2,3,4] Fei Gong,[1,2,3,4] Yueqiu Tan,[1,2,3,4] Guangxiu Lu,[2,3,4] Wei Zheng,[2,3,*] and Ge Lin,[1,2,3,4,*]

*Corresponding author. Email: linggf@hotmail.com (L.G.), ustczw@163.com (Z.W.)

Table of content

Appendix Table S1. Characteristic of two affected individuals.

Appendix Table S2. Down-regulated differentially expressed proteins identified in LC-MS.

Appendix Table S3. Genomic PCR primers of *IQCN* variants in the affected families.

Appendix Table S4. Guide RNA primers of *Iqcn* in mice.

Appendix Table S5. Genomic PCR primers of *Iqcn*-knockout in mice.

Appendix Table S1. Characteristic of two affected individuals.

	II-2 in Family 1	II-1 in Family 2
Male age	35	38
BMI (kg/m^2)	22.49	23.66
Infertility years	10	2
Infertility	Primary infertility	Primary infertility
Phenotype	Normal	Normal
Karyotype	46, XY	46, XY
Ultrasound	No obvious abnormality in prostate, seminal vesicles, testis, epididymis and spermatic veins	Varicocele (left, degree I) No obvious abnormality in prostate, seminal vesicles, testis and epididymis
Medical history	No history of significant illness	No history of significant illness
Habits	No smoking and drinking habits	No smoking and drinking habits

Appendix Table S2. Down-regulated differentially expressed proteins identified in LC-MS.

Protein accession	Protein description	Protein name	LFQ WT-ave	LFQ KO-ave	Ratio
A0A075B5R4	Immunoglobulin heavy variable 14-1 (Fragment) OS=Mus musculus OX=10090 GN=Ighv14-1 PE=4 SV=1	IGHV14-1	48.34	0.02	-2229.11
F6SQH7	Polymerase delta-interacting protein 2 (Fragment) OS=Mus musculus OX=10090 GN=Poldip2 PE=1 SV=1	POLDIP2	6.61	0.02	-304.65
Q6URW6	Myosin-14 OS=Mus musculus OX=10090 GN=Myh14 PE=1 SV=1	MYH14	4.53	0.02	-208.68
Q8VHM5	Heterogeneous nuclear ribonucleoprotein R OS=Mus musculus OX=10090 GN=Hnrnpr PE=1 SV=1	HNRNPR	2.42	0.02	-111.40
Q99PU8	ATP-dependent RNA helicase DHX30 OS=Mus musculus OX=10090 GN=Dhx30 PE=1 SV=1	DHX30	2.40	0.02	-110.76
Q6ZWZ4	60S ribosomal protein L36 OS=Mus musculus OX=10090 GN=Rpl36 PE=1 SV=1	RPL36	1.41	0.02	-64.89
Q9CX86	Heterogeneous nuclear ribonucleoprotein A0 OS=Mus musculus OX=10090 GN=Hnrnpa0 PE=1 SV=1	HNRNPA0	1.40	0.02	-64.64
Q80X56	E3 ubiquitin-protein ligase TRIM69 OS=Mus musculus OX=10090 GN=Trim69 PE=1 SV=1	TRIM69	0.82	0.02	-37.96
Q99MR6	Serrate RNA effector molecule homolog OS=Mus musculus OX=10090 GN=Srrt PE=1 SV=1	SRRT	0.77	0.02	-35.67
Q8BFZ3	Beta-actin-like protein 2 OS=Mus musculus OX=10090 GN=Actbl2 PE=1 SV=1	ACTBL2	0.66	0.02	-30.23
P62849	40S ribosomal protein S24 OS=Mus musculus OX=10090 GN=Rps24 PE=1 SV=1	RPS24	0.59	0.02	-27.25
P62881	Guanine nucleotide-binding protein subunit beta-5 OS=Mus musculus OX=10090 GN=Gnb5 PE=1 SV=1	GNB5	0.57	0.02	-26.48
Q9ERD7	Tubulin beta-3 chain OS=Mus musculus OX=10090 GN=Tubb3 PE=1 SV=1	TUBB3	0.55	0.02	-25.27
Q9QZB7	Actin-related protein 10 OS=Mus musculus OX=10090 GN=Actr10 PE=1 SV=2	ACTR10	0.53	0.02	-24.50
A0A0G2JDW7	40S ribosomal protein S27 (Fragment) OS=Mus musculus OX=10090 GN=Rps27 PE=1 SV=1	RPS27	0.52	0.02	-23.79
O89112	Glutathione S-transferase LANCL1 OS=Mus musculus OX=10090 GN=Lancl1 PE=1 SV=1	LANCL1	0.49	0.02	-22.47
P62897	Cytochrome c, somatic OS=Mus musculus OX=10090 GN=Cycs PE=1 SV=2	CYCS	0.46	0.02	-20.98
D3YV69	Ras-related protein Rab-6A OS=Mus musculus OX=10090 GN=Rab6a PE=1 SV=1	RAB6A	0.43	0.02	-19.79
H3BKQ7	Protein phosphatase 1, regulatory subunit 9A OS=Mus musculus OX=10090 GN=Ppp1r9a PE=1 SV=1	PPP1R9A	0.43	0.02	-19.74
Q9CR68	Cytochrome b-c1 complex subunit Rieske, mitochondrial OS=Mus musculus OX=10090 GN=Uqcrfs1 PE=1 SV=1	UQCRFS1	0.42	0.02	-19.42
P43275	Histone H1.1 OS=Mus musculus OX=10090 GN=H1-1 PE=1 SV=2	H1-1	0.40	0.02	-18.62
Q9CQM8	60S ribosomal protein L21 OS=Mus musculus OX=10090 GN=Rpl21 PE=1 SV=1	RPL21	0.40	0.02	-18.49
P34022	Ran-specific GTPase-activating protein OS=Mus musculus OX=10090 GN=Ranbp1 PE=1 SV=2	RANBP1	0.40	0.02	-18.39
A0A1D5RMD1	IQ motif-containing N OS=Mus musculus OX=10090 GN=Iqcn PE=4 SV=1	IQCN	0.38	0.02	-17.68
A0A3Q4EBV4	Protein FAM98A OS=Mus musculus OX=10090 GN=Fam98a PE=1 SV=1	FAM98A	0.36	0.02	-16.79
P54869	Hydroxymethylglutaryl-CoA synthase, mitochondrial OS=Mus musculus OX=10090 GN=Hmgcs2 PE=1 SV=2	HMGCS2	0.36	0.02	-16.40
O88531	Palmitoyl-protein thioesterase 1 OS=Mus musculus OX=10090 GN=Ppt1 PE=1 SV=2	PPT1	0.35	0.02	-15.96
F6ZV59	Heterogeneous nuclear ribonucleoprotein D0 (Fragment) OS=Mus musculus OX=10090 GN=Hnrnpd PE=1 SV=1	HNRNPD	0.34	0.02	-15.71
A0A1L1SV25	Alpha-actinin-4 OS=Mus musculus OX=10090 GN=Actn4 PE=1 SV=1	ACTN4	0.33	0.02	-15.09
Q9Z2U1	Proteasome subunit alpha type-5 OS=Mus musculus OX=10090 GN=Psma5 PE=1 SV=1	PSMA5	0.32	0.02	-14.96
P14206	40S ribosomal protein SA OS=Mus musculus OX=10090 GN=Rpsa PE=1 SV=4	RPSA	0.30	0.02	-13.78
A0A0G2JEX1	Nexilin OS=Mus musculus OX=10090 GN=Nexn PE=1 SV=1	NEXN	0.29	0.02	-13.43

A2AFK7	RNA helicase (Fragment) OS=Mus musculus OX=10090 GN=Eif4a3 PE=1 SV=1	EIF4A3	0.29	0.02	-13.20
P48771	Cytochrome c oxidase subunit 7A2, mitochondrial OS=Mus musculus OX=10090 GN=Cox7a2 PE=1 SV=2	COX7A2	0.28	0.02	-13.10
P15864	Histone H1.2 OS=Mus musculus OX=10090 GN=H1-2 PE=1 SV=2	H1-2	0.28	0.02	-13.05
A0A494B8X0	Spermatogenesis-associated protein 24 (Fragment) OS=Mus musculus OX=10090 GN=Spata24 PE=4 SV=1	SPATA24	0.25	0.02	-11.51
Q9DB20	ATP synthase subunit O, mitochondrial OS=Mus musculus OX=10090 GN=Atp5po PE=1 SV=1	ATP5PO	0.25	0.02	-11.37
Q9QZB9	Dynactin subunit 5 OS=Mus musculus OX=10090 GN=Dctn5 PE=1 SV=1	DCTN5	0.25	0.02	-11.37
Q3TF41	Nucleosome assembly protein 1-like 1 OS=Mus musculus OX=10090 GN=Nap1l1 PE=1 SV=1	NAP1L1	0.23	0.02	-10.75
Q9R112	Sulfide:quinone oxidoreductase, mitochondrial OS=Mus musculus OX=10090 GN=Sqor PE=1 SV=3	SQOR	0.23	0.02	-10.68
Q5SRX1	TOM1-like protein 2 OS=Mus musculus OX=10090 GN=Tom1l2 PE=1 SV=1	TOM1L2	0.23	0.02	-10.59
Q9Z2H2	Regulator of G-protein signaling 6 OS=Mus musculus OX=10090 GN=Rgs6 PE=1 SV=2	RGS6	0.22	0.02	-10.32
A0A087WRZ5	TAR DNA-binding protein 43 OS=Mus musculus OX=10090 GN=Tardbp PE=1 SV=1	TARDBP	0.22	0.02	-10.28
Q8VDM4	26S proteasome non-ATPase regulatory subunit 2 OS=Mus musculus OX=10090 GN=Psmd2 PE=1 SV=1	PSMD2	0.22	0.02	-10.07
Q9DAJ5	Dynein light chain roadblock-type 2 OS=Mus musculus OX=10090 GN=Dynlrb2 PE=1 SV=1	DYNLRB2	0.21	0.02	-9.69
Q04447	Creatine kinase B-type OS=Mus musculus OX=10090 GN=Ckb PE=1 SV=1	CKB	0.21	0.02	-9.49
G5E902	Phosphate carrier protein, mitochondrial OS=Mus musculus OX=10090 GN=Slc25a3 PE=1 SV=1	SLC25A3	0.20	0.02	-9.29
A0A0R4J0J8	Cytospin-A OS=Mus musculus OX=10090 GN=Specc1l PE=1 SV=1	SPECC1L	0.20	0.02	-9.07
Q9JHQ5	Leucine zipper transcription factor-like protein 1 OS=Mus musculus OX=10090 GN=Lztfl1 PE=1 SV=1	LZTFL1	0.19	0.02	-8.86
Q3U4W8	Ubiquitin carboxyl-terminal hydrolase OS=Mus musculus OX=10090 GN=Usp5 PE=1 SV=1	USP5	0.19	0.02	-8.74
D3YVW0	Metaxin OS=Mus musculus OX=10090 GN=Mtx1 PE=1 SV=3	MTX1	0.19	0.02	-8.60
O35723	DnaJ homolog subfamily B member 3 OS=Mus musculus OX=10090 GN=Dnajb3 PE=2 SV=1	DNAJB3	0.18	0.02	-8.50
Q60718	Disintegrin and metalloproteinase domain-containing protein 2 OS=Mus musculus OX=10090 GN=Adam2 PE=1 SV=2	ADAM2	0.18	0.02	-8.47
O54724	Caveolae-associated protein 1 OS=Mus musculus OX=10090 GN=Cavin1 PE=1 SV=1	CAVIN1	0.18	0.02	-8.29
Q9JJV2	Profilin-2 OS=Mus musculus OX=10090 GN=Pfn2 PE=1 SV=3	PFN2	0.18	0.02	-8.22
P63325	40S ribosomal protein S10 OS=Mus musculus OX=10090 GN=Rps10 PE=1 SV=1	RPS10	0.18	0.02	-8.16
F8WI80	Adenosine deaminase domain-containing protein 1 OS=Mus musculus OX=10090 GN=Adad1 PE=1 SV=1	ADAD1	0.18	0.02	-8.10
O70551	SRSF protein kinase 1 OS=Mus musculus OX=10090 GN=Srpk1 PE=1 SV=2	SRPK1	0.17	0.02	-7.81
I7HFT9	H1.6 linker histone, cluster member OS=Mus musculus OX=10090 GN=H1f6 PE=1 SV=1	H1F6	2.42	0.32	-7.67
Q91Z49	UAP56-interacting factor OS=Mus musculus OX=10090 GN=Fyttd1 PE=1 SV=1	FYTTD1	0.17	0.02	-7.65
Q8BFW7	Lipoma-preferred partner homolog OS=Mus musculus OX=10090 GN=Lpp PE=1 SV=1	LPP	0.16	0.02	-7.53
Q8BH80	Vesicle-associated membrane protein, associated protein B and C OS=Mus musculus OX=10090 GN=Vapb PE=1 SV=1	VAPB	0.16	0.02	-7.46
D3Z563	Acyl-CoA-binding protein OS=Mus musculus OX=10090 GN=Dbi PE=1 SV=1	DBI	0.16	0.02	-7.44
P53395	Lipoamide acyltransferase component of branched-chain alpha-keto acid dehydrogenase complex, mitochondrial OS=Mus musculus OX=10090 GN=Dbt PE=1 SV=2	DBT	0.16	0.02	-7.34

O08749	Dihydrolipoyl dehydrogenase, mitochondrial OS=Mus musculus OX=10090 GN=Dld PE=1 SV=2	DLD	0.16	0.02	-7.33
Q7M6Z4	Kinesin-like protein KIF27 OS=Mus musculus OX=10090 GN=Kif27 PE=1 SV=1	KIF27	0.16	0.02	-7.20
Q923D5	WW domain-binding protein 11 OS=Mus musculus OX=10090 GN=Wbp11 PE=1 SV=2	WBP11	0.16	0.02	-7.19
A2BFF8	Cytoplasmic dynein 1 intermediate chain 2 OS=Mus musculus OX=10090 GN=Dync1i2 PE=1 SV=1	DYNC1I2	0.15	0.02	-7.15
Q8VC66	Afadin- and alpha-actinin-binding protein OS=Mus musculus OX=10090 GN=Ssx2ip PE=1 SV=1	SSX2IP	0.15	0.02	-7.05
P24815	3 beta-hydroxysteroid dehydrogenase/Delta 5-->4-isomerase type 1 OS=Mus musculus OX=10090 GN=Hsd3b1 PE=1 SV=3	HSD3B1	0.15	0.02	-7.05
Q8CGC7	Bifunctional glutamate/proline--tRNA ligase OS=Mus musculus OX=10090 GN=Eprs1 PE=1 SV=4	EPRS1	0.15	0.02	-6.93
Q9QZD9	Eukaryotic translation initiation factor 3 subunit I OS=Mus musculus OX=10090 GN=Eif3i PE=1 SV=1	EIF3I	0.15	0.02	-6.90
A2AED8	Spermatogenesis-associated protein 6 OS=Mus musculus OX=10090 GN=Spata6 PE=1 SV=1	SPATA6	0.15	0.02	-6.89
A0A0N4SVB8	ADP-ribosylation factor-like protein 8B OS=Mus musculus OX=10090 GN=Arl8b PE=1 SV=1	ARL8B	0.15	0.02	-6.85
A0A1L1SSA3	Eukaryotic translation initiation factor 1b (Fragment) OS=Mus musculus OX=10090 GN=Eif1b PE=1 SV=1	EIF1B	0.15	0.02	-6.75
E9PVK4	Tetratricopeptide repeat protein 21B OS=Mus musculus OX=10090 GN=Ttc21b PE=1 SV=1	TTC21B	0.15	0.02	-6.73
Q9JJL0	Testis-specific gene A8 protein OS=Mus musculus OX=10090 GN=Tsga8 PE=1 SV=1	TSGA8	0.15	0.02	-6.72
P23116	Eukaryotic translation initiation factor 3 subunit A OS=Mus musculus OX=10090 GN=Eif3a PE=1 SV=5	EIF3A	0.14	0.02	-6.64
P99028	Cytochrome b-c1 complex subunit 6, mitochondrial OS=Mus musculus OX=10090 GN=Uqcrh PE=1 SV=2	UQCRH	0.14	0.02	-6.64
Q3UMU9	Hepatoma-derived growth factor-related protein 2 OS=Mus musculus OX=10090 GN=Hdgfl2 PE=1 SV=1	HDGFL2	0.14	0.02	-6.55
Q05CL8	La-related protein 7 OS=Mus musculus OX=10090 GN=Larp7 PE=1 SV=2	LARP7	0.14	0.02	-6.51
Q3UJB0	Splicing factor 3b, subunit 2 OS=Mus musculus OX=10090 GN=Sf3b2 PE=1 SV=1	SF3B2	0.14	0.02	-6.47
A0A140LIN9	Dynein heavy chain 3, axonemal OS=Mus musculus OX=10090 GN=Dnah3 PE=1 SV=2	DNAH3	0.14	0.02	-6.46
B1AWE0	Clathrin light chain OS=Mus musculus OX=10090 GN=Clta PE=1 SV=1	CLTA	0.14	0.02	-6.41
A0A0G2JGC1	Serine/threonine-protein phosphatase OS=Mus musculus OX=10090 GN=Ppp1cc PE=1 SV=1	PPP1CC	0.14	0.02	-6.39
P51859	Hepatoma-derived growth factor OS=Mus musculus OX=10090 GN=Hdgf PE=1 SV=2	HDGF	0.14	0.02	-6.38
A0A0R4J1H6	Golgin subfamily A member 3 OS=Mus musculus OX=10090 GN=Golga3 PE=1 SV=1	GOLGA3	0.14	0.02	-6.34
D3Z0R4	Tubby-like protein OS=Mus musculus OX=10090 GN=Tulp2 PE=1 SV=3	TULP2	0.14	0.02	-6.33
Q921M7	CYFIP-related Rac1 interactor B OS=Mus musculus OX=10090 GN=Cyrib PE=1 SV=1	CYRIB	0.14	0.02	-6.31
Z4YK11	SH3 domain-containing protein 21 OS=Mus musculus OX=10090 GN=Sh3d21 PE=1 SV=1	SH3D21	0.14	0.02	-6.29
A0A6Q6PKE1	Tubulin polyglutamylase complex subunit 1 OS=Mus musculus OX=10090 GN=Tpgs1 PE=1 SV=1	TPGS1	0.14	0.02	-6.29
P45377	Aldose reductase-related protein 2 OS=Mus musculus OX=10090 GN=Akr1b8 PE=1 SV=2	AKR1B8	0.14	0.02	-6.28
Q9DAG6	GLIPR1-like protein 1 OS=Mus musculus OX=10090 GN=Glipr1l1 PE=1 SV=1	GLIPR1L1	0.13	0.02	-6.18
Q922Y1	UBX domain-containing protein 1 OS=Mus musculus OX=10090 GN=Ubxn1 PE=1 SV=1	UBXN1	0.13	0.02	-6.10
Q99LF4	RNA-splicing ligase RtcB homolog OS=Mus musculus OX=10090 GN=Rtcb PE=1 SV=1	RTCB	0.13	0.02	-6.09

Q8R1B4	Eukaryotic translation initiation factor 3 subunit C OS=Mus musculus OX=10090 GN=Eif3c PE=1 SV=1	EIF3C	0.13	0.02	-6.02
Q5NC84	Zona pellucida binding protein OS=Mus musculus OX=10090 GN=Zpbp PE=1 SV=1	ZPBP	0.13	0.02	-5.96
Q5H8C4	Vacuolar protein sorting-associated protein 13A OS=Mus musculus OX=10090 GN=Vps13a PE=1 SV=1	VPS13A	0.13	0.02	-5.92
Q924M7	Mannose-6-phosphate isomerase OS=Mus musculus OX=10090 GN=Mpi PE=1 SV=1	MPI	0.13	0.02	-5.80
Q64727	Vinculin OS=Mus musculus OX=10090 GN=Vcl PE=1 SV=4	VCL	0.13	0.02	-5.79
Q3V061	Tripartite motif-containing 80 OS=Mus musculus OX=10090 GN=Trim80 PE=2 SV=1	TRIM80	0.12	0.02	-5.75
Q9CZU6	Citrate synthase, mitochondrial OS=Mus musculus OX=10090 GN=Cs PE=1 SV=1	CS	0.12	0.02	-5.59
O35594	Intraflagellar transport protein 81 homolog OS=Mus musculus OX=10090 GN=Ift81 PE=1 SV=4	IFT81	0.12	0.02	-5.58
D3Z3A0	Protein phosphatase inhibitor 2 OS=Mus musculus OX=10090 GN=Ppp1r2 PE=1 SV=1	PPP1R2	0.12	0.02	-5.58
Q99KJ8	Dynactin subunit 2 OS=Mus musculus OX=10090 GN=Dctn2 PE=1 SV=3	DCTN2	0.12	0.02	-5.52
Q99MV7	RING finger protein 17 OS=Mus musculus OX=10090 GN=Rnf17 PE=1 SV=2	RNF17	0.12	0.02	-5.50
Q9Z130	Heterogeneous nuclear ribonucleoprotein D-like OS=Mus musculus OX=10090 GN=Hnrnpdl PE=1 SV=1	HNRNPDL	0.12	0.02	-5.47
Q9D2H9	Dynein axonemal assembly factor 1 OS=Mus musculus OX=10090 GN=Dnaaf1 PE=1 SV=1	DNAAF1	0.12	0.02	-5.45
P35585	AP-1 complex subunit mu-1 OS=Mus musculus OX=10090 GN=Ap1m1 PE=1 SV=3	AP1M1	0.12	0.02	-5.44
Q8R127	Saccharopine dehydrogenase-like oxidoreductase OS=Mus musculus OX=10090 GN=Sccpdh PE=1 SV=1	SCCPDH	0.12	0.02	-5.42
E9Q3V6	Septin-2 OS=Mus musculus OX=10090 GN=Septin2 PE=1 SV=1	SEPTIN2	0.12	0.02	-5.38
O35887	Calumenin OS=Mus musculus OX=10090 GN=Calu PE=1 SV=1	CALU	0.12	0.02	-5.36
Q00623	Apolipoprotein A-I OS=Mus musculus OX=10090 GN=Apoa1 PE=1 SV=2	APOA1	0.12	0.02	-5.35
P28667	MARCKS-related protein OS=Mus musculus OX=10090 GN=Marcksl1 PE=1 SV=2	MARCKSL1	0.12	0.02	-5.31
A2AKV1	ATP synthase, H+-transporting, mitochondrial F1 complex, gamma polypeptide 1 (Fragment) OS=Mus musculus OX=10090 GN=Atp5c1 PE=1 SV=1	ATP5C1	0.11	0.02	-5.30
P62814	V-type proton ATPase subunit B, brain isoform OS=Mus musculus OX=10090 GN=Atp6v1b2 PE=1 SV=1	ATP6V1B2	0.11	0.02	-5.25
P12787	Cytochrome c oxidase subunit 5A, mitochondrial OS=Mus musculus OX=10090 GN=Cox5a PE=1 SV=2	COX5A	0.11	0.02	-5.14
Q9QY83	Actin-like protein 7B OS=Mus musculus OX=10090 GN=Actl7b PE=1 SV=2	ACTL7B	0.11	0.02	-5.14
E9Q1S9	Cystatin-12 OS=Mus musculus OX=10090 GN=Cst12 PE=1 SV=1	CST12	0.11	0.02	-5.10
Q3TWW8	Serine/arginine-rich splicing factor 6 OS=Mus musculus OX=10090 GN=Srsf6 PE=1 SV=1	SRSF6	0.11	0.02	-5.06
Q8K4Z5	Splicing factor 3A subunit 1 OS=Mus musculus OX=10090 GN=Sf3a1 PE=1 SV=1	SF3A1	0.11	0.02	-5.04
Q9JK53	Prolargin OS=Mus musculus OX=10090 GN=Prelp PE=1 SV=2	PRELP	0.11	0.02	-5.02
Q9CQQ7	ATP synthase F(0) complex subunit B1, mitochondrial OS=Mus musculus OX=10090 GN=Atp5pb PE=1 SV=1	ATP5PB	0.11	0.02	-5.00
F7CDT0	NEDD8-conjugating enzyme Ubc12 (Fragment) OS=Mus musculus OX=10090 GN=Ube2m PE=1 SV=1	UBE2M	0.11	0.02	-4.90
Q91Z31	Polypyrimidine tract-binding protein 2 OS=Mus musculus OX=10090 GN=Ptbp2 PE=1 SV=2	PTBP2	0.11	0.02	-4.87
D3YX34	Dynactin subunit 1 OS=Mus musculus OX=10090 GN=Dctn1 PE=1 SV=1	DCTN1	0.10	0.02	-4.81
Q9Z127	Large neutral amino acids transporter small subunit 1 OS=Mus musculus OX=10090 GN=Slc7a5 PE=1 SV=2	SLC7A5	0.10	0.02	-4.80
Q8CEE6	PAS domain-containing serine/threonine-protein kinase OS=Mus musculus OX=10090 GN=Pask PE=1 SV=3	PASK	0.10	0.02	-4.77
E9Q3Z5	Supervillin OS=Mus musculus OX=10090 GN=Svil PE=1 SV=1	SVIL	0.10	0.02	-4.72

P97473	RISC-loading complex subunit TARBP2 OS=Mus musculus OX=10090 GN=Tarbp2 PE=1 SV=2	TARBP2	0.10	0.02	-4.66
Q8CBY8	Dynactin subunit 4 OS=Mus musculus OX=10090 GN=Dctn4 PE=1 SV=1	DCTN4	0.56	0.12	-4.57
A0A087WRP2	Laminin subunit alpha-2 OS=Mus musculus OX=10090 GN=Lama2 PE=1 SV=1	LAMA2	0.10	0.02	-4.47
E9QAI5	Aspartate carbamoyltransferase OS=Mus musculus OX=10090 GN=Cad PE=1 SV=1	CAD	0.10	0.02	-4.45
Q99JF8	PC4 and SFRS1-interacting protein OS=Mus musculus OX=10090 GN=Psip1 PE=1 SV=1	PSIP1	0.10	0.02	-4.42
A0A2I3BQP6	Calcium/calmodulin-dependent protein kinase OS=Mus musculus OX=10090 GN=Camk2g PE=1 SV=1	CAMK2G	0.38	0.09	-4.40
Q99LC3	NADH dehydrogenase [ubiquinone] 1 alpha subcomplex subunit 10, mitochondrial OS=Mus musculus OX=10090 GN=Ndufa10 PE=1 SV=1	NDUFA10	0.09	0.02	-4.37
P52825	Carnitine O-palmitoyltransferase 2, mitochondrial OS=Mus musculus OX=10090 GN=Cpt2 PE=1 SV=2	CPT2	0.09	0.02	-4.30
Q9DBJ1	Phosphoglycerate mutase 1 OS=Mus musculus OX=10090 GN=Pgam1 PE=1 SV=3	PGAM1	0.09	0.02	-4.30
Q8K450	Sperm-associated antigen 16 protein OS=Mus musculus OX=10090 GN=Spag16 PE=1 SV=1	SPAG16	0.09	0.02	-4.30
O55022	Membrane-associated progesterone receptor component 1 OS=Mus musculus OX=10090 GN=Pgrmc1 PE=1 SV=4	PGRMC1	0.09	0.02	-4.24
Q3TXS7	26S proteasome non-ATPase regulatory subunit 1 OS=Mus musculus OX=10090 GN=Psmd1 PE=1 SV=1	PSMD1	0.09	0.02	-4.24
E9QKR0	Guanine nucleotide-binding protein G(I)/G(S)/G(T) subunit beta-2 OS=Mus musculus OX=10090 GN=Gnb2 PE=1 SV=1	GNB2	0.09	0.02	-4.22
Q9D0B0	Serine/arginine-rich splicing factor 9 OS=Mus musculus OX=10090 GN=Srsf9 PE=1 SV=1	SRSF9	0.09	0.02	-4.19
E9Q5G3	Kinesin-like protein KIF23 OS=Mus musculus OX=10090 GN=Kif23 PE=1 SV=1	KIF23	0.66	0.16	-4.11
H7BX21	Thioredoxin domain-containing protein 2 OS=Mus musculus OX=10090 GN=Txndc2 PE=1 SV=1	TXNDC2	0.09	0.02	-4.11
Q6P4T2	U5 small nuclear ribonucleoprotein 200 kDa helicase OS=Mus musculus OX=10090 GN=Snrnp200 PE=1 SV=1	SNRNP200	0.09	0.02	-4.08
Q925I1	ATPase family AAA domain-containing protein 3 OS=Mus musculus OX=10090 GN=Atad3 PE=1 SV=1	ATAD3	0.09	0.02	-4.06
P70195	Proteasome subunit beta type-7 OS=Mus musculus OX=10090 GN=Psmb7 PE=1 SV=1	PSMB7	0.09	0.02	-4.03
Q91VR5	ATP-dependent RNA helicase DDX1 OS=Mus musculus OX=10090 GN=Ddx1 PE=1 SV=1	DDX1	0.09	0.02	-4.02
Q6VSS7	Reproductive homeobox 8 OS=Mus musculus OX=10090 GN=Rhox8 PE=1 SV=1	RHOX8	0.09	0.02	-4.01
Q8CH09	SURP and G-patch domain-containing protein 2 OS=Mus musculus OX=10090 GN=Sugp2 PE=1 SV=2	SUGP2	0.09	0.02	-4.01
Q6ZWX6	Eukaryotic translation initiation factor 2 subunit 1 OS=Mus musculus OX=10090 GN=Eif2s1 PE=1 SV=3	EIF2S1	0.09	0.02	-4.00
Q8BG95	Protein phosphatase 1 regulatory subunit 12B OS=Mus musculus OX=10090 GN=Ppp1r12b PE=1 SV=2	PPP1R12B	0.09	0.02	-3.97
Q8BVQ9	26S proteasome AAA-ATPase subunit RPT1 OS=Mus musculus OX=10090 GN=Psmc2 PE=1 SV=1	PSMC2	0.09	0.02	-3.97
Q99JI4	26S proteasome non-ATPase regulatory subunit 6 OS=Mus musculus OX=10090 GN=Psmd6 PE=1 SV=1	PSMD6	0.09	0.02	-3.94
O55201	Transcription elongation factor SPT5 OS=Mus musculus OX=10090 GN=Supt5h PE=1 SV=1	SUPT5H	0.09	0.02	-3.92
Q8BKE0	Proteasome subunit alpha type-2 (Fragment) OS=Mus musculus OX=10090 GN=Psma2 PE=1 SV=1	PSMA2	0.08	0.02	-3.91
A0A0B4J1E2	SNW domain-containing protein 1 OS=Mus musculus OX=10090 GN=Snw1 PE=1 SV=1	SNW1	0.08	0.02	-3.91
Q61171	Peroxiredoxin-2 OS=Mus musculus OX=10090 GN=Prdx2 PE=1 SV=3	PRDX2	0.08	0.02	-3.89

Q9Z1W9	STE20/SPS1-related proline-alanine-rich protein kinase OS=Mus musculus OX=10090 GN=Stk39 PE=1 SV=1	STK39	0.08	0.02	-3.87
Q8R050	Eukaryotic peptide chain release factor GTP-binding subunit ERF3A OS=Mus musculus OX=10090 GN=Gspt1 PE=1 SV=2	GSPT1	0.08	0.02	-3.86
D3YWT0	Signal peptidase complex catalytic subunit SEC11 OS=Mus musculus OX=10090 GN=Sec11a PE=1 SV=1	SEC11A	0.08	0.02	-3.85
P60670	Nuclear protein localization protein 4 homolog OS=Mus musculus OX=10090 GN=Nploc4 PE=1 SV=3	NPLOC4	0.08	0.02	-3.85
P60766	Cell division control protein 42 homolog OS=Mus musculus OX=10090 GN=Cdc42 PE=1 SV=2	CDC42	0.08	0.02	-3.85
Q8BL82	Calreticulin-3 OS=Mus musculus OX=10090 GN=Calr3 PE=1 SV=1	CALR3	0.08	0.02	-3.83
O70194	Eukaryotic translation initiation factor 3 subunit D OS=Mus musculus OX=10090 GN=Eif3d PE=1 SV=2	EIF3D	0.08	0.02	-3.83
Q9DCH4	Eukaryotic translation initiation factor 3 subunit F OS=Mus musculus OX=10090 GN=Eif3f PE=1 SV=2	EIF3F	0.08	0.02	-3.80
Q3UYV9	Nuclear cap-binding protein subunit 1 OS=Mus musculus OX=10090 GN=Ncbp1 PE=1 SV=2	NCBP1	0.08	0.02	-3.80
B1AVH5	Coronin OS=Mus musculus OX=10090 GN=Coro2a PE=1 SV=1	CORO2A	0.43	0.11	-3.78
A0A338P7J7	Radial spoke head protein 3 homolog A OS=Mus musculus OX=10090 GN=Rsph3a PE=3 SV=1	RSPH3A	0.08	0.02	-3.78
Q9JJ28	Protein flightless-1 homolog OS=Mus musculus OX=10090 GN=Flii PE=1 SV=1	FLII	0.39	0.10	-3.77
Q9JHX6	Probable allantoicase OS=Mus musculus OX=10090 GN=Allc PE=1 SV=2	ALLC	0.08	0.02	-3.75
P14152	Malate dehydrogenase, cytoplasmic OS=Mus musculus OX=10090 GN=Mdh1 PE=1 SV=3	MDH1	0.08	0.02	-3.75
Q8CF20	RIKEN cDNA 1700020N01 gene OS=Mus musculus OX=10090 GN=1700020N01Rik PE=1 SV=1	1700020N01RIK	0.08	0.02	-3.75
Q9WTP6	Adenylate kinase 2, mitochondrial OS=Mus musculus OX=10090 GN=Ak2 PE=1 SV=5	AK2	0.08	0.02	-3.74
O35593	26S proteasome non-ATPase regulatory subunit 14 OS=Mus musculus OX=10090 GN=Psmd14 PE=1 SV=2	PSMD14	0.08	0.02	-3.73
G3UY93	Valyl-tRNA synthetase (Fragment) OS=Mus musculus OX=10090 GN=Vars PE=1 SV=1	VARS	0.08	0.02	-3.73
Q9CQA3	Succinate dehydrogenase [ubiquinone] iron-sulfur subunit, mitochondrial OS=Mus musculus OX=10090 GN=Sdhb PE=1 SV=1	SDHB	0.08	0.02	-3.68
Q9QZ06	Toll-interacting protein OS=Mus musculus OX=10090 GN=Tollip PE=1 SV=1	TOLLIP	0.32	0.09	-3.62
Q9WTQ5	A-kinase anchor protein 12 OS=Mus musculus OX=10090 GN=Akap12 PE=1 SV=1	AKAP12	0.08	0.02	-3.62
A0A3B2WB12	Male-enhanced antigen 1 (Fragment) OS=Mus musculus OX=10090 GN=Mea1 PE=1 SV=1	MEA1	0.08	0.02	-3.60
B1ARW4	Complex I-15 kDa (Fragment) OS=Mus musculus OX=10090 GN=Ndufs5 PE=1 SV=1	NDUFS5	0.08	0.02	-3.58
F8VQB6	Unconventional myosin-X OS=Mus musculus OX=10090 GN=Myo10 PE=1 SV=1	MYO10	0.08	0.02	-3.56
A0A0R4IZY0	Thimet oligopeptidase OS=Mus musculus OX=10090 GN=Thop1 PE=1 SV=1	THOP1	0.08	0.02	-3.54
Q9JKK7	Tropomodulin-2 OS=Mus musculus OX=10090 GN=Tmod2 PE=1 SV=2	TMOD2	1.55	0.44	-3.53
A0A0R4J1Q0	Enhancer of mRNA-decapping protein 4 OS=Mus musculus OX=10090 GN=Edc4 PE=1 SV=1	EDC4	0.08	0.02	-3.51
A0A1D5RLS2	Cleavage and polyadenylation specificity factor subunit 5 (Fragment) OS=Mus musculus OX=10090 GN=Nudt21 PE=1 SV=1	NUDT21	0.08	0.02	-3.51
P70675	Testis-specific protein TSX OS=Mus musculus OX=10090 GN=Tsx PE=1 SV=1	TSX	0.08	0.02	-3.49
D3YWV9	Gametocyte-specific factor 1 (Fragment) OS=Mus musculus OX=10090 GN=Gtsf1 PE=1 SV=1	GTSF1	0.07	0.02	-3.46
Q8CC70	Cilia- and flagella-associated protein 300 OS=Mus musculus OX=10090 GN=Cfap300 PE=2 SV=1	CFAP300	0.07	0.02	-3.46

A0A3B2WBE1	Band 4.1-like protein 3 (Fragment) OS=Mus musculus OX=10090 GN=Epb41l3 PE=1 SV=1	EPB41L3	0.07	0.02	-3.43
Q8C0C7	Phenylalanine--tRNA ligase alpha subunit OS=Mus musculus OX=10090 GN=Farsa PE=1 SV=1	FARSA	0.07	0.02	-3.40
A0A0R4J0F3	Protein phosphatase 1 regulatory subunit 32 OS=Mus musculus OX=10090 GN=Ppp1r32 PE=1 SV=1	PPP1R32	0.07	0.02	-3.40
Q91XQ0	Dynein axonemal heavy chain 8 OS=Mus musculus OX=10090 GN=Dnah8 PE=1 SV=2	DNAH8	0.07	0.02	-3.39
Q9DBR7	Protein phosphatase 1 regulatory subunit 12A OS=Mus musculus OX=10090 GN=Ppp1r12a PE=1 SV=2	PPP1R12A	0.41	0.12	-3.39
A0A087WQE6	Elongin-C (Fragment) OS=Mus musculus OX=10090 GN=Eloc PE=1 SV=1	ELOC	0.07	0.02	-3.37
Q3UKC1	Tax1-binding protein 1 homolog OS=Mus musculus OX=10090 GN=Tax1bp1 PE=1 SV=2	TAX1BP1	0.07	0.02	-3.37
Q9JLV5	Cullin-3 OS=Mus musculus OX=10090 GN=Cul3 PE=1 SV=1	CUL3	0.07	0.02	-3.34
Q8VCW8	Medium-chain acyl-CoA ligase ACSF2, mitochondrial OS=Mus musculus OX=10090 GN=Acsf2 PE=1 SV=1	ACSF2	0.07	0.02	-3.33
P97819	85/88 kDa calcium-independent phospholipase A2 OS=Mus musculus OX=10090 GN=Pla2g6 PE=1 SV=3	PLA2G6	0.07	0.02	-3.31
P28474	Alcohol dehydrogenase class-3 OS=Mus musculus OX=10090 GN=Adh5 PE=1 SV=3	ADH5	0.07	0.02	-3.31
Q9R099	Transducin beta-like protein 2 OS=Mus musculus OX=10090 GN=Tbl2 PE=1 SV=2	TBL2	0.07	0.02	-3.28
Q80ZJ2	Inositol-1-monophosphatase OS=Mus musculus OX=10090 GN=Impa1 PE=1 SV=1	IMPA1	0.07	0.02	-3.28
Q99NB9	Splicing factor 3B subunit 1 OS=Mus musculus OX=10090 GN=Sf3b1 PE=1 SV=1	SF3B1	0.07	0.02	-3.28
A2AH85	116 kDa U5 small nuclear ribonucleoprotein component OS=Mus musculus OX=10090 GN=Eftud2 PE=1 SV=1	EFTUD2	0.07	0.02	-3.28
Q99MV1	Tudor domain-containing protein 1 OS=Mus musculus OX=10090 GN=Tdrd1 PE=1 SV=2	TDRD1	0.07	0.02	-3.27
P62075	Mitochondrial import inner membrane translocase subunit Tim13 OS=Mus musculus OX=10090 GN=Timm13 PE=1 SV=1	TIMM13	0.07	0.02	-3.26
Q9CQ43	Deoxyuridine 5'-triphosphate nucleotidohydrolase OS=Mus musculus OX=10090 GN=Dut PE=1 SV=1	DUT	0.07	0.02	-3.25
P17426	AP-2 complex subunit alpha-1 OS=Mus musculus OX=10090 GN=Ap2a1 PE=1 SV=1	AP2A1	0.07	0.02	-3.25
Q9CQM9	Glutaredoxin-3 OS=Mus musculus OX=10090 GN=Glrx3 PE=1 SV=1	GLRX3	0.07	0.02	-3.25
Q91VD9	NADH-ubiquinone oxidoreductase 75 kDa subunit, mitochondrial OS=Mus musculus OX=10090 GN=Ndufs1 PE=1 SV=2	NDUFS1	0.07	0.02	-3.24
Q9WUA2	Phenylalanine--tRNA ligase beta subunit OS=Mus musculus OX=10090 GN=Farsb PE=1 SV=2	FARSB	0.07	0.02	-3.22
Q91ZT8	Ankyrin repeat and SOCS box protein 9 OS=Mus musculus OX=10090 GN=Asb9 PE=1 SV=2	ASB9	0.07	0.02	-3.20
Q9CR86	Calcium-regulated heat stable protein 1 OS=Mus musculus OX=10090 GN=Carhsp1 PE=1 SV=1	CARHSP1	0.07	0.02	-3.19
Q91V41	Ras-related protein Rab-14 OS=Mus musculus OX=10090 GN=Rab14 PE=1 SV=3	RAB14	0.07	0.02	-3.18
Q6VH22	Intraflagellar transport protein 172 homolog OS=Mus musculus OX=10090 GN=Ift172 PE=1 SV=1	IFT172	0.24	0.07	-3.16
P14685	26S proteasome non-ATPase regulatory subunit 3 OS=Mus musculus OX=10090 GN=Psmd3 PE=1 SV=3	PSMD3	0.07	0.02	-3.13
Q9D8L3	Signal sequence receptor subunit delta OS=Mus musculus OX=10090 GN=Ssr4 PE=1 SV=1	SSR4	0.07	0.02	-3.13
G3X928	SEC23-interacting protein OS=Mus musculus OX=10090 GN=Sec23ip PE=1 SV=1	SEC23IP	0.07	0.02	-3.12
Q5RKN9	F-actin-capping protein subunit alpha OS=Mus musculus OX=10090 GN=Capza1 PE=1 SV=1	CAPZA1	1.53	0.49	-3.12
P70168	Importin subunit beta-1 OS=Mus musculus OX=10090 GN=Kpnb1 PE=1 SV=2	KPNB1	0.07	0.02	-3.10

Q8CDR2	Radial spoke head protein 6 homolog A OS=Mus musculus OX=10090 GN=Rsph6a PE=1 SV=1	RSPH6A	0.07	0.02	-3.09
Q9CQB4	Cytochrome b-c1 complex subunit 7 OS=Mus musculus OX=10090 GN=Uqcrb PE=1 SV=1	UQCRB	0.07	0.02	-3.09
Q9JHJ0	Tropomodulin-3 OS=Mus musculus OX=10090 GN=Tmod3 PE=1 SV=1	TMOD3	1.39	0.45	-3.08
Q3UEB3	Poly(U)-binding-splicing factor PUF60 OS=Mus musculus OX=10090 GN=Puf60 PE=1 SV=2	PUF60	0.07	0.02	-3.08
O54774	AP-3 complex subunit delta-1 OS=Mus musculus OX=10090 GN=Ap3d1 PE=1 SV=1	AP3D1	0.07	0.02	-3.06
Q9JKV1	Proteasomal ubiquitin receptor ADRM1 OS=Mus musculus OX=10090 GN=Adrm1 PE=1 SV=2	ADRM1	0.07	0.02	-3.05
Q9D515	Slx-like 1 OS=Mus musculus OX=10090 GN=Slxl1 PE=2 SV=1	SLXL1	0.07	0.02	-3.04
Q9WUM3	Coronin-1B OS=Mus musculus OX=10090 GN=Coro1b PE=1 SV=1	CORO1B	0.07	0.02	-3.03
B2KF24	T-complex protein 11 OS=Mus musculus OX=10090 GN=Tcp11 PE=1 SV=2	TCP11	0.28	0.09	-3.03
Q8JZP9	GAS2-like protein 1 OS=Mus musculus OX=10090 GN=Gas2l1 PE=1 SV=1	GAS2L1	0.28	0.09	-3.02
Q61768	Kinesin-1 heavy chain OS=Mus musculus OX=10090 GN=Kif5b PE=1 SV=3	KIF5B	0.28	0.09	-3.02
E0CZ27	Histone H3 (Fragment) OS=Mus musculus OX=10090 GN=H3f3a PE=3 SV=1	H3F3A	2.53	0.84	-3.02
A2AX52	Collagen alpha-4(VI) chain OS=Mus musculus OX=10090 GN=Col6a4 PE=1 SV=2	COL6A4	0.07	0.02	-3.00
Q99JY0	Trifunctional enzyme subunit beta, mitochondrial OS=Mus musculus OX=10090 GN=Hadhb PE=1 SV=1	HADHB	1.73	0.58	-2.98
B1AWG1	Intraflagellar transport protein 74 homolog OS=Mus musculus OX=10090 GN=Ift74 PE=1 SV=1	IFT74	0.06	0.02	-2.97
Q02053	Ubiquitin-like modifier-activating enzyme 1 OS=Mus musculus OX=10090 GN=Uba1 PE=1 SV=1	UBA1	0.06	0.02	-2.95
B2RXP1	DISP complex protein LRCH3 OS=Mus musculus OX=10090 GN=Lrch3 PE=1 SV=1	LRCH3	0.62	0.21	-2.93
Q8VDP4	Cell cycle and apoptosis regulator protein 2 OS=Mus musculus OX=10090 GN=Ccar2 PE=1 SV=2	CCAR2	0.06	0.02	-2.91
A0A0R4J083	Long-chain specific acyl-CoA dehydrogenase, mitochondrial OS=Mus musculus OX=10090 GN=Acadl PE=1 SV=1	ACADL	0.06	0.02	-2.91
Q3UGF1	WD repeat-containing protein 19 OS=Mus musculus OX=10090 GN=Wdr19 PE=1 SV=1	WDR19	0.06	0.02	-2.90
A0A0N4SUV6	Selenocysteine-specific elongation factor OS=Mus musculus OX=10090 GN=Eefsec PE=1 SV=1	EEFSEC	0.06	0.02	-2.87
P61027	Ras-related protein Rab-10 OS=Mus musculus OX=10090 GN=Rab10 PE=1 SV=1	RAB10	0.06	0.02	-2.86
E9QNH6	Unconventional myosin-Ib OS=Mus musculus OX=10090 GN=Myo1b PE=1 SV=1	MYO1B	0.51	0.18	-2.83
Q9ERE7	LRP chaperone MESD OS=Mus musculus OX=10090 GN=Mesd PE=1 SV=1	MESD	0.06	0.02	-2.81
E9PXY1	Cullin-4B OS=Mus musculus OX=10090 GN=Cul4b PE=1 SV=2	CUL4B	0.06	0.02	-2.81
A0A1B0GR78	Stromal interaction molecule 1 OS=Mus musculus OX=10090 GN=Stim1 PE=1 SV=1	STIM1	0.06	0.02	-2.80
A2AN08	E3 ubiquitin-protein ligase UBR4 OS=Mus musculus OX=10090 GN=Ubr4 PE=1 SV=1	UBR4	0.06	0.02	-2.80
O70252	Heme oxygenase 2 OS=Mus musculus OX=10090 GN=Hmox2 PE=1 SV=1	HMOX2	0.06	0.02	-2.77
Q8BQ30	Phostensin OS=Mus musculus OX=10090 GN=Ppp1r18 PE=1 SV=1	PPP1R18	0.35	0.13	-2.76
B1AYJ9	Obg-like ATPase 1 OS=Mus musculus OX=10090 GN=Ola1 PE=1 SV=1	OLA1	0.06	0.02	-2.75
A0A0B4J1E7	Importin subunit alpha-3 OS=Mus musculus OX=10090 GN=Kpna4 PE=1 SV=1	KPNA4	0.06	0.02	-2.73
Q9EQH3	Vacuolar protein sorting-associated protein 35 OS=Mus musculus OX=10090 GN=Vps35 PE=1 SV=1	VPS35	0.06	0.02	-2.72

P53994	Ras-related protein Rab-2A OS=Mus musculus OX=10090 GN=Rab2a PE=1 SV=1	RAB2A	0.30	0.11	-2.71
A0A2R8W6P6	GDP-4-keto-6-deoxy-D-mannose-3,5-epimerase-4-reductase OS=Mus musculus OX=10090 GN=Gfus PE=1 SV=1	GFUS	0.06	0.02	-2.66
Q8CGP5	Histone H2A type 1-F OS=Mus musculus OX=10090 GN=Hist1h2af PE=1 SV=3	HIST1H2AF	8.88	3.34	-2.66
Q45VK7	Cytoplasmic dynein 2 heavy chain 1 OS=Mus musculus OX=10090 GN=Dync2h1 PE=1 SV=1	DYNC2H1	0.06	0.02	-2.65
Q99PV0	Pre-mRNA-processing-splicing factor 8 OS=Mus musculus OX=10090 GN=Prpf8 PE=1 SV=2	PRPF8	0.16	0.06	-2.65
G3X9J0	Signal-induced proliferation-associated 1-like protein 3 OS=Mus musculus OX=10090 GN=Sipa1l3 PE=1 SV=1	SIPA1L3	0.06	0.02	-2.64
Q3UM45	Protein phosphatase 1 regulatory subunit 7 OS=Mus musculus OX=10090 GN=Ppp1r7 PE=1 SV=2	PPP1R7	0.06	0.02	-2.63
Q921H8	3-ketoacyl-CoA thiolase A, peroxisomal OS=Mus musculus OX=10090 GN=Acaa1a PE=1 SV=1	ACAA1A	0.06	0.02	-2.62
Q9DAI2	Intraflagellar transport protein 22 homolog OS=Mus musculus OX=10090 GN=Ift22 PE=1 SV=1	IFT22	0.06	0.02	-2.61
P42125	Enoyl-CoA delta isomerase 1, mitochondrial OS=Mus musculus OX=10090 GN=Eci1 PE=1 SV=2	ECI1	0.06	0.02	-2.61
Q9JJ78	Lymphokine-activated killer T-cell-originated protein kinase OS=Mus musculus OX=10090 GN=Pbk PE=1 SV=1	PBK	0.06	0.02	-2.59
G3X972	Sec24-related gene family, member C (S. cerevisiae) OS=Mus musculus OX=10090 GN=Sec24c PE=1 SV=1	SEC24C	0.06	0.02	-2.58
Q9CR16	Peptidyl-prolyl cis-trans isomerase D OS=Mus musculus OX=10090 GN=Ppid PE=1 SV=3	PPID	0.06	0.02	-2.58
Q8BKC5	Importin-5 OS=Mus musculus OX=10090 GN=Ipo5 PE=1 SV=3	IPO5	0.19	0.08	-2.57
A0A3Q4EI12	Ras-related protein Rab-18 OS=Mus musculus OX=10090 GN=Rab18 PE=1 SV=1	RAB18	0.06	0.02	-2.56
Q7TSC1	Protein PRRC2A OS=Mus musculus OX=10090 GN=Prrc2a PE=1 SV=1	PRRC2A	0.06	0.02	-2.56
Q9Z2N8	Actin-like protein 6A OS=Mus musculus OX=10090 GN=Actl6a PE=1 SV=2	ACTL6A	0.06	0.02	-2.55
Q9D1Q6	Endoplasmic reticulum resident protein 44 OS=Mus musculus OX=10090 GN=Erp44 PE=1 SV=1	ERP44	0.05	0.02	-2.52
Q8R2P8	Lysine--tRNA ligase OS=Mus musculus OX=10090 GN=Kars PE=1 SV=1	KARS	0.05	0.02	-2.52
O35083	1-acyl-sn-glycerol-3-phosphate acyltransferase alpha OS=Mus musculus OX=10090 GN=Agpat1 PE=1 SV=1	AGPAT1	0.05	0.02	-2.51
A3KGU5	Spectrin alpha chain, non-erythrocytic 1 OS=Mus musculus OX=10090 GN=Sptan1 PE=1 SV=1	SPTAN1	0.39	0.15	-2.50
A0A1L1SQA8	40S ribosomal protein S25 OS=Mus musculus OX=10090 GN=Rps25 PE=1 SV=1	RPS25	0.95	0.38	-2.50
Q9CX34	Protein SGT1 homolog OS=Mus musculus OX=10090 GN=Sugt1 PE=1 SV=3	SUGT1	0.05	0.02	-2.50
A0A0N4SUM3	Lysophospholipid acyltransferase 5 OS=Mus musculus OX=10090 GN=Lpcat3 PE=1 SV=1	LPCAT3	0.05	0.02	-2.49
Q8C2Q3	RNA-binding protein 14 OS=Mus musculus OX=10090 GN=Rbm14 PE=1 SV=1	RBM14	0.42	0.17	-2.48
Q8C6E0	Cilia- and flagella-associated protein 36 OS=Mus musculus OX=10090 GN=Cfap36 PE=1 SV=1	CFAP36	0.18	0.07	-2.47
D3YYM6	40S ribosomal protein S5 (Fragment) OS=Mus musculus OX=10090 GN=Rps5 PE=1 SV=1	RPS5	0.43	0.18	-2.44
Q9WU65	Glycerol kinase 2 OS=Mus musculus OX=10090 GN=Gk2 PE=1 SV=1	GK2	0.05	0.02	-2.44
Q61029	Lamina-associated polypeptide 2, isoforms beta/delta/epsilon/gamma OS=Mus musculus OX=10090 GN=Tmpo PE=1 SV=4	TMPO	0.05	0.02	-2.44
P57784	U2 small nuclear ribonucleoprotein A' OS=Mus musculus OX=10090 GN=Snrpa1 PE=1 SV=2	SNRPA1	0.05	0.02	-2.44
Q9WU78	Programmed cell death 6-interacting protein OS=Mus musculus OX=10090 GN=Pdcd6ip PE=1 SV=3	PDCD6IP	0.05	0.02	-2.43

A0A171KXD3	Protein arginine N-methyltransferase 1 OS=Mus musculus OX=10090 GN=Prmt1 PE=1 SV=1	PRMT1	0.05	0.02	-2.43
Q9D4K7	Coiled-coil domain-containing protein 105 OS=Mus musculus OX=10090 GN=Ccdc105 PE=1 SV=3	CCDC105	0.29	0.12	-2.42
Q6ZQ38	Cullin-associated NEDD8-dissociated protein 1 OS=Mus musculus OX=10090 GN=Cand1 PE=1 SV=2	CAND1	0.20	0.08	-2.41
Q9DCN2	NADH-cytochrome b5 reductase 3 OS=Mus musculus OX=10090 GN=Cyb5r3 PE=1 SV=3	CYB5R3	0.05	0.02	-2.41
Q60864	Stress-induced-phosphoprotein 1 OS=Mus musculus OX=10090 GN=Stip1 PE=1 SV=1	STIP1	0.22	0.09	-2.39
O35129	Prohibitin-2 OS=Mus musculus OX=10090 GN=Phb2 PE=1 SV=1	PHB2	0.31	0.13	-2.39
Q61884	Meiosis-specific nuclear structural protein 1 OS=Mus musculus OX=10090 GN=Mns1 PE=1 SV=1	MNS1	0.15	0.06	-2.39
P40142	Transketolase OS=Mus musculus OX=10090 GN=Tkt PE=1 SV=1	TKT	0.05	0.02	-2.38
Q8BKZ9	Pyruvate dehydrogenase protein X component, mitochondrial OS=Mus musculus OX=10090 GN=Pdhx PE=1 SV=1	PDHX	0.05	0.02	-2.36
P61982	14-3-3 protein gamma OS=Mus musculus OX=10090 GN=Ywhag PE=1 SV=2	YWHAG	0.17	0.07	-2.35
D3YVB4	40S ribosomal protein S15a (Fragment) OS=Mus musculus OX=10090 GN=Rps15a PE=1 SV=1	RPS15A	0.83	0.35	-2.35
Q80UG5	Septin-9 OS=Mus musculus OX=10090 GN=Septin9 PE=1 SV=1	SEPTIN9	0.05	0.02	-2.34
O88374	Branched-chain-amino-acid aminotransferase OS=Mus musculus OX=10090 GN=Bcat2 PE=1 SV=1	BCAT2	0.05	0.02	-2.33
P09405	Nucleolin OS=Mus musculus OX=10090 GN=Ncl PE=1 SV=2	NCL	0.32	0.14	-2.33
O35490	Betaine--homocysteine S-methyltransferase 1 OS=Mus musculus OX=10090 GN=Bhmt PE=1 SV=1	BHMT	0.05	0.02	-2.32
A2AEX8	Four and a half LIM domains protein 1 OS=Mus musculus OX=10090 GN=Fhl1 PE=1 SV=1	FHL1	0.05	0.02	-2.32
B7ZNL2	Nap1l4 protein OS=Mus musculus OX=10090 GN=Nap1l4 PE=1 SV=1	NAP1L4	0.15	0.07	-2.32
A0A1L1STE6	Isocitrate dehydrogenase [NAD] subunit, mitochondrial OS=Mus musculus OX=10090 GN=Idh3a PE=1 SV=1	IDH3A	0.05	0.02	-2.29
Q5STT6	Protein FAM71B OS=Mus musculus OX=10090 GN=Fam71b PE=3 SV=3	FAM71B	0.45	0.20	-2.29
Q8C633	Calcium-binding and spermatid-specific protein 1 OS=Mus musculus OX=10090 GN=Cabs1 PE=1 SV=1	CABS1	0.22	0.10	-2.29
Q9WVK4	EH domain-containing protein 1 OS=Mus musculus OX=10090 GN=Ehd1 PE=1 SV=1	EHD1	0.05	0.02	-2.28
Q9ERK4	Exportin-2 OS=Mus musculus OX=10090 GN=Cse1l PE=1 SV=1	CSE1L	0.05	0.02	-2.28
P20108	Thioredoxin-dependent peroxide reductase, mitochondrial OS=Mus musculus OX=10090 GN=Prdx3 PE=1 SV=1	PRDX3	0.05	0.02	-2.27
Q80VD1	Protein FAM98B OS=Mus musculus OX=10090 GN=Fam98b PE=1 SV=1	FAM98B	0.05	0.02	-2.27
D3YXL1	Cancer-related nucleoside-triphosphatase homolog OS=Mus musculus OX=10090 GN=Ntpcr PE=1 SV=1	NTPCR	0.05	0.02	-2.26
P60229	Eukaryotic translation initiation factor 3 subunit E OS=Mus musculus OX=10090 GN=Eif3e PE=1 SV=1	EIF3E	0.05	0.02	-2.25
P52293	Importin subunit alpha-1 OS=Mus musculus OX=10090 GN=Kpna2 PE=1 SV=2	KPNA2	0.05	0.02	-2.25
Q5XJY5	Coatomer subunit delta OS=Mus musculus OX=10090 GN=Arcn1 PE=1 SV=2	ARCN1	0.05	0.02	-2.24
Q60972	Histone-binding protein RBBP4 OS=Mus musculus OX=10090 GN=Rbbp4 PE=1 SV=5	RBBP4	0.19	0.09	-2.24
P58389	Serine/threonine-protein phosphatase 2A activator OS=Mus musculus OX=10090 GN=Ptpa PE=1 SV=1	PTPA	0.22	0.10	-2.22
Q9DCW4	Electron transfer flavoprotein subunit beta OS=Mus musculus OX=10090 GN=Etfb PE=1 SV=3	ETFB	0.05	0.02	-2.22
Q3TT92	Dihydropyrimidinase-related protein 3 OS=Mus musculus OX=10090 GN=Dpysl3 PE=1 SV=1	DPYSL3	0.05	0.02	-2.21
P27612	Phospholipase A-2-activating protein OS=Mus musculus OX=10090 GN=Plaa PE=1 SV=4	PLAA	0.05	0.02	-2.20

P09671	Superoxide dismutase [Mn], mitochondrial OS=Mus musculus OX=10090 GN=Sod2 PE=1 SV=3	SOD2	0.25	0.11	-2.20
E9Q317	18 kDa Sin3-associated polypeptide OS=Mus musculus OX=10090 GN=Sap18 PE=1 SV=1	SAP18	0.21	0.09	-2.20
A6PWC3	Nardilysin, N-arginine dibasic convertase, NRD convertase 1 OS=Mus musculus OX=10090 GN=Nrd1 PE=1 SV=1	NRD1	0.05	0.02	-2.19
D3Z158	Glutaminyl-tRNA synthetase OS=Mus musculus OX=10090 GN=Qars PE=1 SV=2	QARS	0.05	0.02	-2.18
Q8JZQ9	Eukaryotic translation initiation factor 3 subunit B OS=Mus musculus OX=10090 GN=Eif3b PE=1 SV=1	EIF3B	0.39	0.18	-2.18
Q99PU5	Long-chain-fatty-acid--CoA ligase ACSBG1 OS=Mus musculus OX=10090 GN=Acsbg1 PE=1 SV=1	ACSBG1	0.08	0.04	-2.18
O88844	Isocitrate dehydrogenase [NADP] cytoplasmic OS=Mus musculus OX=10090 GN=Idh1 PE=1 SV=2	IDH1	0.05	0.02	-2.18
Q9CQE8	RNA transcription, translation and transport factor protein OS=Mus musculus OX=10090 GN=RTRAF PE=1 SV=1	RTRAF	0.22	0.10	-2.17
P08249	Malate dehydrogenase, mitochondrial OS=Mus musculus OX=10090 GN=Mdh2 PE=1 SV=3	MDH2	0.44	0.20	-2.17
P40336	Vacuolar protein sorting-associated protein 26A OS=Mus musculus OX=10090 GN=Vps26a PE=1 SV=1	VPS26A	0.05	0.02	-2.16
O70200	Allograft inflammatory factor 1 OS=Mus musculus OX=10090 GN=Aif1 PE=1 SV=1	AIF1	1.90	0.88	-2.15
E9QK36	WD repeat-containing protein 62 OS=Mus musculus OX=10090 GN=Wdr62 PE=1 SV=1	WDR62	0.13	0.06	-2.15
Q8CDC8	Four and a half LIM domains 4 OS=Mus musculus OX=10090 GN=Fhl4 PE=1 SV=1	FHL4	0.05	0.02	-2.14
P47754	F-actin-capping protein subunit alpha-2 OS=Mus musculus OX=10090 GN=Capza2 PE=1 SV=3	CAPZA2	1.70	0.80	-2.14
Q99M87	DnaJ homolog subfamily A member 3, mitochondrial OS=Mus musculus OX=10090 GN=Dnaja3 PE=1 SV=1	DNAJA3	0.21	0.10	-2.13
Q5DTX6	Junctional protein associated with coronary artery disease OS=Mus musculus OX=10090 GN=Jcad PE=1 SV=2	JCAD	0.16	0.08	-2.12
Q9JHU9	Inositol-3-phosphate synthase 1 OS=Mus musculus OX=10090 GN=Isyna1 PE=1 SV=1	ISYNA1	0.32	0.15	-2.12
Q8BMS1	Trifunctional enzyme subunit alpha, mitochondrial OS=Mus musculus OX=10090 GN=Hadha PE=1 SV=1	HADHA	1.78	0.84	-2.12
Q9D9L5	Actin-related protein T2 OS=Mus musculus OX=10090 GN=Actrt2 PE=2 SV=1	ACTRT2	0.16	0.08	-2.10
A2A6Q8	Myosin light chain 4 (Fragment) OS=Mus musculus OX=10090 GN=Myl4 PE=1 SV=8	MYL4	1.64	0.79	-2.09
F6RJV6	LanC-like protein 2 (Fragment) OS=Mus musculus OX=10090 GN=Lancl2 PE=1 SV=1	LANCL2	0.20	0.09	-2.09
P18572	Basigin OS=Mus musculus OX=10090 GN=Bsg PE=1 SV=2	BSG	0.23	0.11	-2.08
A0A1Y7VJ48	Tubulin-specific chaperone A OS=Mus musculus OX=10090 GN=Tbca PE=1 SV=1	TBCA	0.24	0.12	-2.08
Q60749	KH domain-containing, RNA-binding, signal transduction-associated protein 1 OS=Mus musculus OX=10090 GN=Khdrbs1 PE=1 SV=2	KHDRBS1	0.58	0.28	-2.08
Q9D8B3	Charged multivesicular body protein 4b OS=Mus musculus OX=10090 GN=Chmp4b PE=1 SV=2	CHMP4B	0.32	0.15	-2.08
A0A0G2JEP4	Leucine-rich repeat flightless-interacting protein 2 OS=Mus musculus OX=10090 GN=Lrrfip2 PE=1 SV=1	LRRFIP2	1.20	0.58	-2.07
Q02788	Collagen alpha-2(VI) chain OS=Mus musculus OX=10090 GN=Col6a2 PE=1 SV=3	COL6A2	0.45	0.22	-2.07
P27546	Microtubule-associated protein 4 OS=Mus musculus OX=10090 GN=Map4 PE=1 SV=3	MAP4	0.08	0.04	-2.06
B2RY50	Outer dynein arm-docking complex subunit 2 OS=Mus musculus OX=10090 GN=Odad2 PE=1 SV=1	ODAD2	0.04	0.02	-2.03
P31428	Dipeptidase 1 OS=Mus musculus OX=10090 GN=Dpep1 PE=1 SV=2	DPEP1	0.04	0.02	-2.03
Q3TWV4	AP-2 complex subunit mu OS=Mus musculus OX=10090 GN=Ap2m1 PE=1 SV=1	AP2M1	0.17	0.08	-2.03

P61089	Ubiquitin-conjugating enzyme E2 N OS=Mus musculus OX=10090 GN=Ube2n PE=1 SV=1	UBE2N	0.31	0.15	-2.03
Q8BVN8	Axonemal dynein light intermediate polypeptide 1 OS=Mus musculus OX=10090 GN=Dnali1 PE=1 SV=1	DNALI1	2.24	1.11	-2.02
Q62348	Translin OS=Mus musculus OX=10090 GN=Tsn PE=1 SV=1	TSN	0.16	0.08	-2.02
A0A087WRM0	Protein CDV3 (Fragment) OS=Mus musculus OX=10090 GN=Cdv3 PE=1 SV=1	CDV3	0.04	0.02	-2.01
Q9D4J1	EF-hand domain-containing protein D1 OS=Mus musculus OX=10090 GN=Efhd1 PE=1 SV=1	EFHD1	1.40	0.70	-2.00
Q6P5F9	Exportin-1 OS=Mus musculus OX=10090 GN=Xpo1 PE=1 SV=1	XPO1	0.04	0.02	-1.99
Q9DAP0	Leucine-rich repeat-containing protein 46 OS=Mus musculus OX=10090 GN=Lrrc46 PE=1 SV=2	LRRC46	0.21	0.10	-1.99
Q3U1U6	TSC22 domain family protein 4 OS=Mus musculus OX=10090 GN=Tsc22d4 PE=1 SV=1	TSC22D4	0.47	0.23	-1.99
A2AGN7	26S proteasome regulatory subunit 6A OS=Mus musculus OX=10090 GN=Psmc3 PE=1 SV=1	PSMC3	0.22	0.11	-1.99
P49312	Heterogeneous nuclear ribonucleoprotein A1 OS=Mus musculus OX=10090 GN=Hnrnpa1 PE=1 SV=2	HNRNPA1	0.04	0.02	-1.98
E9PY46	Intraflagellar transport protein 140 homolog OS=Mus musculus OX=10090 GN=Ift140 PE=1 SV=1	IFT140	0.67	0.34	-1.97
O54734	Dolichyl-diphosphooligosaccharide--protein glycosyltransferase 48 kDa subunit OS=Mus musculus OX=10090 GN=Ddost PE=1 SV=2	DDOST	0.35	0.18	-1.96
E9Q405	Unconventional myosin-XVIIIa OS=Mus musculus OX=10090 GN=Myo18a PE=1 SV=1	MYO18A	0.36	0.18	-1.96
Q9EP89	Serine beta-lactamase-like protein LACTB, mitochondrial OS=Mus musculus OX=10090 GN=Lactb PE=1 SV=1	LACTB	0.30	0.15	-1.94
P70372	ELAV-like protein 1 OS=Mus musculus OX=10090 GN=Elavl1 PE=1 SV=2	ELAVL1	0.37	0.19	-1.94
E9PVA8	eIF-2-alpha kinase activator GCN1 OS=Mus musculus OX=10090 GN=Gcn1 PE=1 SV=1	GCN1	0.04	0.02	-1.93
G3UZI2	Heterogeneous nuclear ribonucleoprotein Q OS=Mus musculus OX=10090 GN=Syncrip PE=1 SV=1	SYNCRIP	0.04	0.02	-1.91
P14131	40S ribosomal protein S16 OS=Mus musculus OX=10090 GN=Rps16 PE=1 SV=4	RPS16	1.43	0.75	-1.91
Q8VDM6	Heterogeneous nuclear ribonucleoprotein U-like protein 1 OS=Mus musculus OX=10090 GN=Hnrnpul1 PE=1 SV=1	HNRNPUL1	0.34	0.18	-1.91
Q9CXW4	60S ribosomal protein L11 OS=Mus musculus OX=10090 GN=Rpl11 PE=1 SV=4	RPL11	1.28	0.67	-1.90
A0A087WQS2	Basic leucine zipper and W2 domain-containing protein 1 OS=Mus musculus OX=10090 GN=Bzw1 PE=1 SV=1	BZW1	0.04	0.02	-1.90
P09528	Ferritin heavy chain OS=Mus musculus OX=10090 GN=Fth1 PE=1 SV=2	FTH1	0.32	0.17	-1.90
B9EHJ3	Tight junction protein ZO-1 OS=Mus musculus OX=10090 GN=Tjp1 PE=1 SV=1	TJP1	0.61	0.33	-1.89
P54775	26S proteasome regulatory subunit 6B OS=Mus musculus OX=10090 GN=Psmc4 PE=1 SV=2	PSMC4	0.19	0.10	-1.88
Q810Q5	Normal mucosa of esophagus-specific gene 1 protein OS=Mus musculus OX=10090 GN=Nmes1 PE=1 SV=1	NMES1	0.93	0.50	-1.88
F6YVP7	40S ribosomal protein S18 OS=Mus musculus OX=10090 GN=Rps18-ps6 PE=3 SV=2	RPS18-PS6	2.37	1.26	-1.88
P68040	Receptor of activated protein C kinase 1 OS=Mus musculus OX=10090 GN=Rack1 PE=1 SV=3	RACK1	0.26	0.14	-1.88
Q0P5Y3	Tubulin polymerization-promoting protein family member 2 OS=Mus musculus OX=10090 GN=Tppp2 PE=2 SV=1	TPPP2	0.04	0.02	-1.88
Q9R0P9	Ubiquitin carboxyl-terminal hydrolase isozyme L1 OS=Mus musculus OX=10090 GN=Uchl1 PE=1 SV=1	UCHL1	0.62	0.33	-1.88
Q5SVG5	AP complex subunit beta OS=Mus musculus OX=10090 GN=Ap1b1 PE=1 SV=1	AP1B1	0.04	0.02	-1.88
D3YV10	Coiled-coil domain-containing protein 13 OS=Mus musculus OX=10090 GN=Ccdc13 PE=1 SV=1	CCDC13	0.35	0.19	-1.87
Q9CYA0	Protein disulfide isomerase Creld2 OS=Mus musculus OX=10090 GN=Creld2 PE=1 SV=1	CRELD2	0.21	0.11	-1.86

Q61792	LIM and SH3 domain protein 1 OS=Mus musculus OX=10090 GN=Lasp1 PE=1 SV=1	LASP1	0.24	0.13	-1.84
O08795	Glucosidase 2 subunit beta OS=Mus musculus OX=10090 GN=Prkcsh PE=1 SV=1	PRKCSH	0.04	0.02	-1.84
G5E8G6	Unconventional myosin-Vb OS=Mus musculus OX=10090 GN=Myo5b PE=1 SV=1	MYO5B	0.53	0.29	-1.84
P56480	ATP synthase subunit beta, mitochondrial OS=Mus musculus OX=10090 GN=Atp5f1b PE=1 SV=2	ATP5F1B	0.80	0.44	-1.83
B1AU75	Nuclear autoantigenic sperm protein OS=Mus musculus OX=10090 GN=Nasp PE=1 SV=1	NASP	0.43	0.24	-1.82
Q8K410	Disintegrin and metalloproteinase domain-containing protein 32 OS=Mus musculus OX=10090 GN=Adam32 PE=1 SV=3	ADAM32	0.04	0.02	-1.81
A0A494BAJ6	ADP-ribosylation factor-like protein 3 OS=Mus musculus OX=10090 GN=Arl3 PE=1 SV=1	ARL3	0.44	0.24	-1.81
A0A087WS46	Eukaryotic translation elongation factor 1 beta 2 OS=Mus musculus OX=10090 GN=Eef1b2 PE=1 SV=1	EEF1B2	0.24	0.13	-1.80
Q9WTX5	S-phase kinase-associated protein 1 OS=Mus musculus OX=10090 GN=Skp1 PE=1 SV=3	SKP1	0.49	0.28	-1.78
A0A1L1SV73	Ubiquitin carboxyl-terminal hydrolase 47 OS=Mus musculus OX=10090 GN=Usp47 PE=1 SV=1	USP47	0.04	0.02	-1.78
A0A0J9YUS5	Eukaryotic translation initiation factor 4 gamma 1 OS=Mus musculus OX=10090 GN=Eif4g1 PE=1 SV=1	EIF4G1	0.04	0.02	-1.77
P11378	Nuclear transition protein 2 OS=Mus musculus OX=10090 GN=Tnp2 PE=2 SV=3	TNP2	1.90	1.07	-1.77
P58021	Transmembrane 9 superfamily member 2 OS=Mus musculus OX=10090 GN=Tm9sf2 PE=1 SV=1	TM9SF2	0.04	0.02	-1.76
A0A1B0GRV4	Dickkopf-like protein 1 OS=Mus musculus OX=10090 GN=Dkkl1 PE=1 SV=1	DKKL1	0.31	0.18	-1.75
P84104	Serine/arginine-rich splicing factor 3 OS=Mus musculus OX=10090 GN=Srsf3 PE=1 SV=1	SRSF3	0.66	0.38	-1.74
Q99KV1	DnaJ homolog subfamily B member 11 OS=Mus musculus OX=10090 GN=Dnajb11 PE=1 SV=1	DNAJB11	0.32	0.19	-1.74
P63276	40S ribosomal protein S17 OS=Mus musculus OX=10090 GN=Rps17 PE=1 SV=2	RPS17	1.86	1.07	-1.74
P16627	Heat shock 70 kDa protein 1-like OS=Mus musculus OX=10090 GN=Hspa1l PE=1 SV=4	HSPA1L	0.24	0.14	-1.73
Q9QXD6	Fructose-1,6-bisphosphatase 1 OS=Mus musculus OX=10090 GN=Fbp1 PE=1 SV=3	FBP1	0.08	0.04	-1.73
Q8VCQ8	Caldesmon 1 OS=Mus musculus OX=10090 GN=Cald1 PE=1 SV=1	CALD1	1.03	0.60	-1.73
Q2VPR5	HDGF-like 1 OS=Mus musculus OX=10090 GN=Hdgfl1 PE=1 SV=1	HDGFL1	0.17	0.10	-1.73
P30416	Peptidyl-prolyl cis-trans isomerase FKBP4 OS=Mus musculus OX=10090 GN=Fkbp4 PE=1 SV=5	FKBP4	0.26	0.15	-1.72
A0A0N4SVS6	Cellular nucleic acid-binding protein OS=Mus musculus OX=10090 GN=Cnbp PE=1 SV=1	CNBP	0.55	0.32	-1.72
Q9DCX2	ATP synthase subunit d, mitochondrial OS=Mus musculus OX=10090 GN=Atp5pd PE=1 SV=3	ATP5PD	0.34	0.20	-1.71
Q8R326	Paraspeckle component 1 OS=Mus musculus OX=10090 GN=Pspc1 PE=1 SV=1	PSPC1	0.17	0.10	-1.70
Q9D051	Pyruvate dehydrogenase E1 component subunit beta, mitochondrial OS=Mus musculus OX=10090 GN=Pdhb PE=1 SV=1	PDHB	0.41	0.24	-1.70
Q5SW75	Protein phosphatase Slingshot homolog 2 OS=Mus musculus OX=10090 GN=Ssh2 PE=1 SV=2	SSH2	0.17	0.10	-1.69
P06745	Glucose-6-phosphate isomerase OS=Mus musculus OX=10090 GN=Gpi PE=1 SV=4	GPI	0.29	0.17	-1.69
Q8CDE2	Calicin OS=Mus musculus OX=10090 GN=Ccin PE=1 SV=1	CCIN	0.54	0.32	-1.69
A0A0G2JEU1	Aldehyde dehydrogenase, mitochondrial OS=Mus musculus OX=10090 GN=Aldh2 PE=1 SV=1	ALDH2	0.17	0.10	-1.68
Q9EQ00	Ropporin-1-like protein OS=Mus musculus OX=10090 GN=Ropn1l PE=1 SV=2	ROPN1L	0.22	0.13	-1.67

Q60737	Casein kinase II subunit alpha OS=Mus musculus OX=10090 GN=Csnk2a1 PE=1 SV=2	CSNK2A1	0.25	0.15	-1.66
A0A0R4J2A3	Transketolase-like protein 2 OS=Mus musculus OX=10090 GN=Tktl2 PE=1 SV=1	TKTL2	0.11	0.07	-1.65
P38647	Stress-70 protein, mitochondrial OS=Mus musculus OX=10090 GN=Hspa9 PE=1 SV=3	HSPA9	0.98	0.60	-1.65
Q9DA69	Intraflagellar transport protein 43 homolog OS=Mus musculus OX=10090 GN=Ift43 PE=1 SV=2	IFT43	0.21	0.13	-1.64
Q99104	Unconventional myosin-Va OS=Mus musculus OX=10090 GN=Myo5a PE=1 SV=2	MYO5A	0.92	0.56	-1.64
Q8CBB6	Histone H2B OS=Mus musculus OX=10090 GN=H2bc24 PE=2 SV=1	H2BC24	10.93	6.68	-1.64
Q80XR5	U2 snRNP auxiliary factor large subunit OS=Mus musculus OX=10090 GN=U2af2 PE=1 SV=1	U2AF2	0.40	0.25	-1.64
Q9Z204	Heterogeneous nuclear ribonucleoproteins C1/C2 OS=Mus musculus OX=10090 GN=Hnrnpc PE=1 SV=1	HNRNPC	0.46	0.28	-1.63
Q8VIG3	Radial spoke head 1 homolog OS=Mus musculus OX=10090 GN=Rsph1 PE=1 SV=2	RSPH1	0.67	0.41	-1.63
A0A2I3BRL8	Predicted gene 7324 OS=Mus musculus OX=10090 GN=Gm7324 PE=4 SV=1	GM7324	0.37	0.23	-1.62
Q91X72	Hemopexin OS=Mus musculus OX=10090 GN=Hpx PE=1 SV=2	HPX	0.12	0.08	-1.62
Q9ERG0	LIM domain and actin-binding protein 1 OS=Mus musculus OX=10090 GN=Lima1 PE=1 SV=3	LIMA1	0.96	0.59	-1.62
Q3U2G2	Heat shock 70 kDa protein 4 OS=Mus musculus OX=10090 GN=Hspa4 PE=1 SV=1	HSPA4	0.16	0.10	-1.62
P0DP28	Calmodulin-3 OS=Mus musculus OX=10090 GN=Calm3 PE=1 SV=1	CALM3	14.04	8.68	-1.62
P62141	Serine/threonine-protein phosphatase PP1-beta catalytic subunit OS=Mus musculus OX=10090 GN=Ppp1cb PE=1 SV=3	PPP1CB	0.48	0.30	-1.61
Q9DBF1	Alpha-aminoadipic semialdehyde dehydrogenase OS=Mus musculus OX=10090 GN=Aldh7a1 PE=1 SV=4	ALDH7A1	0.03	0.02	-1.61
P34884	Macrophage migration inhibitory factor OS=Mus musculus OX=10090 GN=Mif PE=1 SV=2	MIF	0.37	0.23	-1.60
Q9D5Y1	Coiled-coil domain-containing protein 39 OS=Mus musculus OX=10090 GN=Ccdc39 PE=1 SV=1	CCDC39	0.55	0.35	-1.60
Q921I1	Serotransferrin OS=Mus musculus OX=10090 GN=Tf PE=1 SV=1	TF	0.07	0.04	-1.59
Q62465	Synaptic vesicle membrane protein VAT-1 homolog OS=Mus musculus OX=10090 GN=Vat1 PE=1 SV=3	VAT1	0.03	0.02	-1.58
Q810N3	Ly6/PLAUR domain-containing 10 OS=Mus musculus OX=10090 GN=Lypd10 PE=1 SV=1	LYPD10	0.34	0.22	-1.58
Q3U1J4	DNA damage-binding protein 1 OS=Mus musculus OX=10090 GN=Ddb1 PE=1 SV=2	DDB1	0.10	0.07	-1.57
P42932	T-complex protein 1 subunit theta OS=Mus musculus OX=10090 GN=Cct8 PE=1 SV=3	CCT8	0.25	0.16	-1.57
Q9WUM5	Succinate--CoA ligase [ADP/GDP-forming] subunit alpha, mitochondrial OS=Mus musculus OX=10090 GN=Suclg1 PE=1 SV=4	SUCLG1	0.50	0.32	-1.57
Q9D0M3	Cytochrome c1, heme protein, mitochondrial OS=Mus musculus OX=10090 GN=Cyc1 PE=1 SV=1	CYC1	0.15	0.09	-1.57
P16045	Galectin-1 OS=Mus musculus OX=10090 GN=Lgals1 PE=1 SV=3	LGALS1	0.21	0.13	-1.56
A0A286YCQ5	Nidogen-2 (Fragment) OS=Mus musculus OX=10090 GN=Nid2 PE=1 SV=1	NID2	0.16	0.10	-1.56
Q9CXW3	Calcyclin-binding protein OS=Mus musculus OX=10090 GN=Cacybp PE=1 SV=1	CACYBP	0.24	0.15	-1.55
Q922G7	Tektin-2 OS=Mus musculus OX=10090 GN=Tekt2 PE=1 SV=1	TEKT2	0.29	0.19	-1.55
O35901	G7b alternative form OS=Mus musculus OX=10090 GN=Lsm2 PE=1 SV=1	LSM2	0.03	0.02	-1.55
Q61166	Microtubule-associated protein RP/EB family member 1 OS=Mus musculus OX=10090 GN=Mapre1 PE=1 SV=3	MAPRE1	0.03	0.02	-1.54
P67871	Casein kinase II subunit beta OS=Mus musculus OX=10090 GN=Csnk2b PE=1 SV=1	CSNK2B	0.39	0.25	-1.54
P09103	Protein disulfide-isomerase OS=Mus musculus OX=10090 GN=P4hb PE=1 SV=2	P4HB	0.14	0.09	-1.53

Q9CZ13	Cytochrome b-c1 complex subunit 1, mitochondrial OS=Mus musculus OX=10090 GN=Uqcrc1 PE=1 SV=2	UQCRC1	0.15	0.10	-1.53
E9PZF0	Nucleoside diphosphate kinase OS=Mus musculus OX=10090 GN=Gm20390 PE=3 SV=1	GM20390	0.51	0.33	-1.53
A0A338P7E5	Ubiquitin-conjugating enzyme E2 L3 OS=Mus musculus OX=10090 GN=Ube2l3 PE=1 SV=1	UBE2L3	0.25	0.17	-1.53
A0A3B2WBH9	Tight junction protein ZO-2 OS=Mus musculus OX=10090 GN=Tjp2 PE=1 SV=1	TJP2	0.36	0.24	-1.52
P48722	Heat shock 70 kDa protein 4L OS=Mus musculus OX=10090 GN=Hspa4l PE=1 SV=2	HSPA4L	0.47	0.31	-1.52
P70296	Phosphatidylethanolamine-binding protein 1 OS=Mus musculus OX=10090 GN=Pebp1 PE=1 SV=3	PEBP1	2.05	1.36	-1.51
Q3UYG1	Coiled-coil domain-containing protein 160 OS=Mus musculus OX=10090 GN=Ccdc160 PE=2 SV=1	CCDC160	0.14	0.09	-1.51
Q9QY84	Actin-like protein 7A OS=Mus musculus OX=10090 GN=Actl7a PE=1 SV=1	ACTL7A	0.40	0.27	-1.51
A0A498WGK6	Transmembrane 9 superfamily member OS=Mus musculus OX=10090 GN=Tm9sf3 PE=1 SV=1	TM9SF3	0.03	0.02	-1.51
D3Z789	Sorting nexin-3 OS=Mus musculus OX=10090 GN=Snx3 PE=1 SV=1	SNX3	0.07	0.05	-1.51
A0A0R4J0X5	Alpha-1-antitrypsin 1-3 OS=Mus musculus OX=10090 GN=Serpina1c PE=1 SV=1	SERPINA1C	0.27	0.18	-1.51
Q45VK5	Interleukin enhancer-binding factor 3 OS=Mus musculus OX=10090 GN=Ilf3 PE=1 SV=1	ILF3	0.17	0.11	-1.50
Q6ZWQ9	Myosin, light chain 12A, regulatory, non-sarcomeric OS=Mus musculus OX=10090 GN=Myl12a PE=1 SV=1	MYL12A	7.68	5.12	-1.50

Appendix Table S3. Genomic PCR primers of *IQCN* variants in the affected families.

	Primer Sequence (5′–3′)
Family-1-F	GAAGGGAAACCTGAGGTCGG
Family-1-R	GGAGGAGGGGGACATACCTT
Family-2-F	TCGGCCAATTTACCCTGCAT
Family-2-R	GGCAGCCGATCACAGACATA

Appendix Table S4. Guide RNA primers of *Iqcn* in mice.

	Primer Sequence (5′–3′)	PAM
gRNA-A1	TCACTGCACAGTGACGCATC	TGG
gRNA-A2	CCCATGTGGTATCTATAGTT	AGG

Appendix Table S5. Genomic PCR primers of *Iqcn*-knockout in mice.

	Primer Sequence (5′–3′)
m-F1	TGGTATTTCTAACAGAACCAGCC
m-R1	ATGTGGTGTGTTCCTACATGCCG
m-F2	CTCACCACGGATGCACTCTTG
