# Supplementary material for: IQCN disruption causes fertilization failure and male infertility due to manchette assembly defect
Source: EMBO Mol Med. 2022 Nov 2;14(12):e16501. doi: 10.15252/emmm.202216501 (PMC9728048; doi:10.15252/emmm.202216501)
Supplement: Supplementary file 2 — Expanded View Figures PDF [file EMMM-14-e16501-s001.pdf]

Expanded View Figures

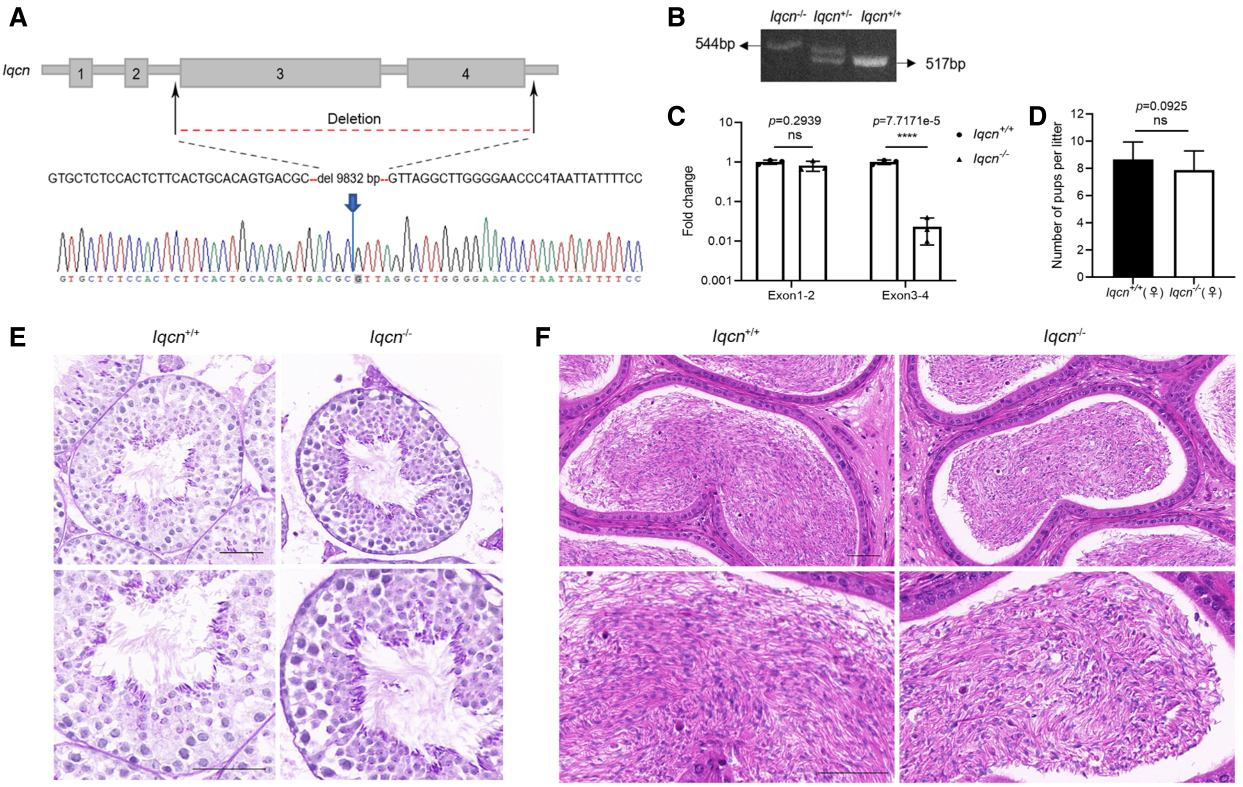

Figure EV1. Identification of *Iqcn*-knockout mice and analysis of spermatogenesis.

A Schematic diagram of the *Iqcn*-knockout strategy; confirmation by Sanger sequencing is shown below the diagram.

B Identification of *Iqcn* homozygous knockout mice by gel electrophoresis.

C Validation of the knockout efficiency in mice by qRT–PCR ($n = 3$). The fold changes of exons 1–2 and exons 3–4 are shown in the histogram.

D Fertility assessment experiments in WT and *Iqcn*$^{-/-}$ female mice after mating with WT male mice ($n = 18$).

E The histomorphology of seminiferous tubules by PAS staining of testes in WT and *Iqcn*$^{-/-}$ male mice. Scale bars, 50 μm.

F The histomorphology of epididymis in WT and *Iqcn*$^{-/-}$ male mice. Scale bars, 50 μm.

Data information: Data represent mean ± SEM. ****$P < 0.0001$, ns represents no significance by the one-way ANOVA (C) or by the unpaired Student's *t*-test (D).

 © 2022 The Authors

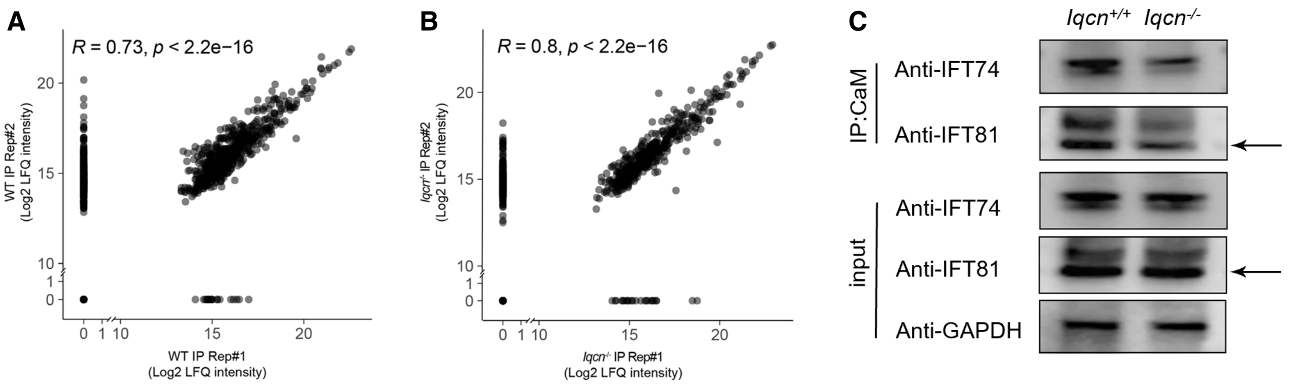

Figure EV2. Data quality control between the two groups of replicates.

A Mass spectrometry results from two replicates in the WT group.

B Mass spectrometry results from two replicates in the *Iqcn*$^{-/-}$ group.

C Interaction of calmodulin with IFT74 and IFT81 following co-IP by calmodulin antibody in testes from WT and *Iqcn*$^{-/-}$ male mice (n = 3). The interaction of calmodulin with IFT74 and IFT81 was lower in *Iqcn*$^{-/-}$ mice than in WT mice. GAPDH was used as the internal control.

Data information: Mass spectrometry results evaluated by the Spearman's correlation analysis (A and B).
Source data are available online for this figure.

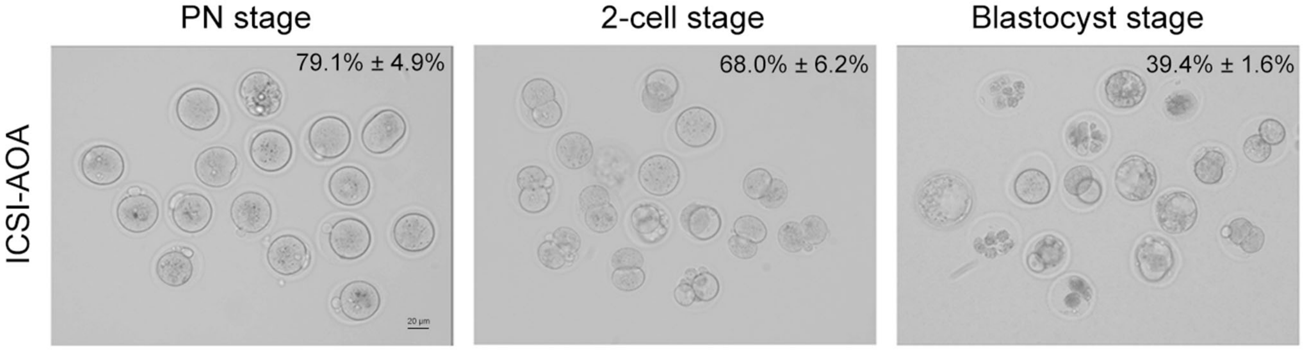

Figure EV3. Outcomes of ICSI with AOA treatment using sperm from *Iqcn*$^{-/-}$ mice.

The percentages of PN stage, 2-cell stage, and blastocyst stage were 79.1% ± 4.9%, 68.0% ± 6.2%, and 39.4% ± 1.6%, respectively (n = 3 biological replicates). Scale bars, 20 μm.

© 2022 The Authors

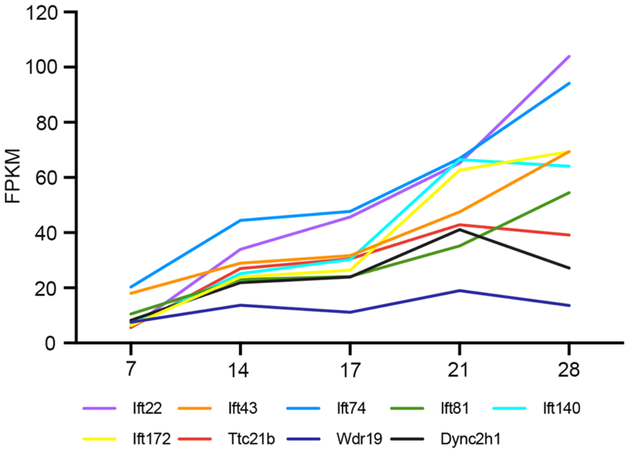

Figure EV4. Expression of IFT family genes during spermatogenesis.

According to the transcriptome sequencing data of Laiho *et al* (2013), the expression of IFT family genes increased with the growth of development days during spermatogenesis.

 © 2022 The Authors
