# Supplementary figures and images for: IQCN disruption causes fertilization failure and male infertility due to manchette assembly defect
Source: EMBO Mol Med. 2022 Nov 2;14(12):e16501. doi: 10.15252/emmm.202216501 (PMC9728048; doi:10.15252/emmm.202216501)

1) IP:IQCEN: Anti-CaM

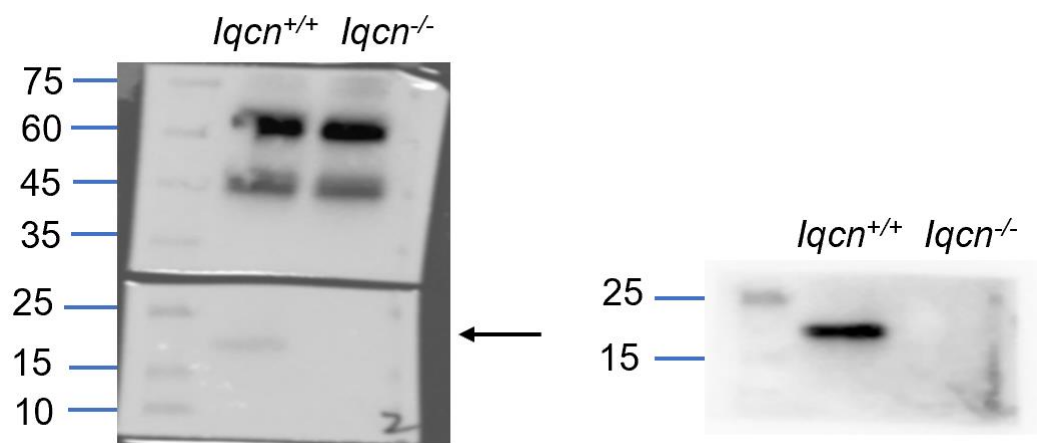

2) IP:IQCEN Anti-IQCEN

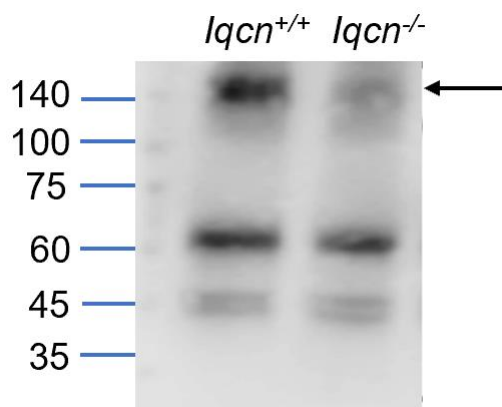

3) Input: Anti-CaM

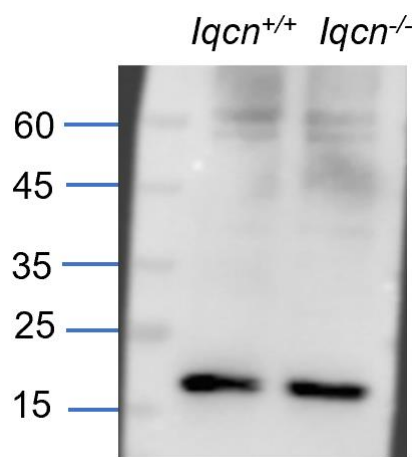

4) Input: Anti-IQCN

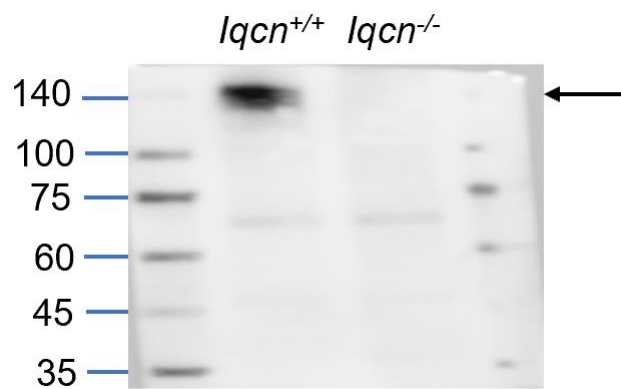

5) Input: Anti-GAPDH

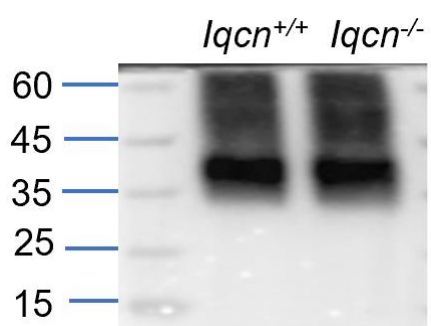

Supplement: Supplementary file 8 — Source Data for Figure 6 [file EMMM-14-e16501-s006.zip › EMM-2022-16501-V3-Figure_6_Source_Data-sd.pdf]
