# Supplementary material for: IQCN disruption causes fertilization failure and male infertility due to manchette assembly defect
Source: EMBO Mol Med. 2022 Nov 2;14(12):e16501. doi: 10.15252/emmm.202216501 (PMC9728048; doi:10.15252/emmm.202216501)
Supplement: Supplementary file 9 — PDF+ [file EMMM-14-e16501-s010.pdf]

Article

SOURCE DATA | TRANSPARENT PROCESS | OPEN ACCESS

EMBO Molecular Medicine

IQCN disruption causes fertilization failure and male infertility due to manchette assembly defect

Jing Dai[1,2,3,†], Qi Li[4,†], Qinwei Zhou[2,3], Shen Zhang[2,3], Junru Chen[2,3], Yize Wang[1], Jing Guo[2,3], Yifan Gu[1,2,3,5], Fei Gong[1,2,3,5], Yueqiu Tan[1,2,3,5], Guangxiu Lu[2,3,5], Wei Zheng[2,3,*] & Ge Lin[1,2,3,5,**]

Abstract

Total fertilization failure (TFF) is an important cause of infertility; however, the genetic basis of TFF caused by male factors remains to be clarified. In this study, whole-exome sequencing was firstly used to screen for genetic causes of TFF after intracytoplasmic sperm injection (ICSI), and homozygous variants in the novel gene IQ motif-containing N (*IQCN*) were identified in two affected individuals with abnormal acrosome structures. Then, *Iqcn*-knockout mice were generated by CRISPR-Cas9 technology and showed that the knockout male mice resembled the human phenotypes. Additionally, we found that IQCN regulates microtubule nucleation during manchette assembly via calmodulin and related calmodulin-binding proteins, which resulted in head deformity with aberrant oocyte activation factor PLCζ. Fortunately, ICSI with assisted oocyte activation can overcome IQCN-associate TFF and male infertility. Thus, our study firstly identified the function of *IQCN*, highlights the relationship between the manchette assembly and fertilization, and provides a genetic marker and a therapeutic option for male-source TFF.

Keywords calmodulin; fertilization failure; IQCN; manchette; PLCζ
Subject Categories Development; Urogenital System
DOI 10.15252/emmm.202216501 | Received 27 June 2022 | Revised 29 September 2022 | Accepted 10 October 2022 | Published online 2 November 2022
EMBO Mol Med (2022) 14: e16501

Introduction

Fertilization is the process by which male and female gametes are fused, which initiates the development of a new organism. Total fertilization failure (TFF) is a frequently encountered difficulty in assisted reproductive technology (ART); indeed, TFF in intracytoplasmic sperm injection (ICSI) carries a 1–3% incidence, nearly half of which is caused by male factors (van der Westerlaken *et al*, 2005; Kashir *et al*, 2010; Nasr-Esfahani *et al*, 2010). Among them, severe sperm head deformity is a common male-source TFF.

For a long period, only globozoospermia is known to have sperm head deformity, which can cause TFF because of the absence of an acrosome structure and the loss of sperm-borne oocyte activation factor PLCζ (Modarres *et al*, 2019). Recently, both our group and that of Sun separately reported a novel type of head deformity, characterized as acrosome detachment, which is responsible for TFF, and identified the causative genes, *ACTL9* and *ACTL7A* (Xin *et al*, 2020; Dai *et al*, 2021). These actin-like proteins are located in the acroplaxome, which participates in acrosome anchoring. As for acrosome specialization and sperm head shaping, the acroplaxome and manchette are two momentous structures that cooperate with various functional proteins to ensure the success of the process.

The manchette is a temporary structure that participates in the elongation and condensation of the spermatid nucleus during spermiogenesis (Kierszenbaum, 2002). The structure consists of a perinuclear ring and inserted microtubular structure (Khawar *et al*, 2019). Previous studies have suggested that microtubule-related proteins or intraflagellar transport (IFT) might participate in manchette assembly, and defects of such genes in mice, such as *Kif3a*, *Ift88*, and *Clip-170*, are known to cause sperm head deformity and infertility (Chen *et al*, 2016). Sperm head shaping is accompanied by acrosome specialization. However, whether the defects in the manchette lead to aberrant acrosome structure and whether this type of sperm deformity is related to human TFF remains unclear.

The novel gene, IQ motif-containing N (*IQCN*) (HGNC: 29350, GenBank: NM_001145304.2) is located on chromosome 19 and has four exons, encoding 1,180 amino acids. The Genotype-Tissue Expression (GTEx) database showed that *IQCN* was specifically expressed in the testis. Bioinformatics analysis showed that IQCN contains six IQ motifs (IQxxxRGxxxR or I/L/VQxxxRxxxxR/K). Two independent studies suggested that IQ motif-containing proteins could recruit calmodulin, which is a postacrosomal sheath-restricted expressed protein in sperm (Li *et al*, 2014; Fang *et al*, 2015). However, no information about the expression and function of *IQCN* has yet been reported.

1 Institute of Reproductive and Stem Cell Engineering, School of Basic Medical Science, Central South University, ChangSha, China
2 Reproductive and Genetic Hospital of CITIC-XIANGYA, ChangSha, China
3 Clinical Research Center for Reproduction and Genetics in Hunan Province, ChangSha, China
4 Reproductive Medicine Center, Xiangya Hospital, Central South University, ChangSha, China
5 Laboratory of Reproductive and Stem Cell Engineering, National Health and Family Planning Commission, ChangSha, China
*Corresponding author. Tel: +86 18670362652; E-mail: ustczw@163.com
**Corresponding author. Tel: +86 13187057355; E-mail: linggf@hotmail.com
†These authors contributed equally to this work

© 2022 The Authors. Published under the terms of the CC BY 4.0 license.

Here, for the first time, we report that IQCN is essential for spermiogenesis and fertilization. Two homozygous pathogenic variants in *IQCN* in TFF cohorts were identified by whole-exome sequencing (WES). We confirmed that sperm from *Iqcn*-knockout (*Iqcn*$^{-/-}$) mice represented as TFF phenotypes. Moreover, we found calmodulin-IQCN interaction loss in *Iqcn*$^{-/-}$ mice, which affects manchette assembly and resulted in sperm head deformity and aberrant PLCζ localization. Remarkably, ICSI with assisted oocyte activation (AOA) treatment is an effective treatment for both *Iqcn*$^{-/-}$ mice and men harboring homozygous *IQCN* variants. Collectively, our results present a novel genetic diagnostic indicator of male-source TFF and infertility and suggest a positive treatment for men carrying homozygous *IQCN* variants.

Results

WES identified homozygous variants in a novel gene *IQCN* in the male factor TFF cohort

To identify the genetic cause of TFF in men, we conducted WES analyses in a male factor TFF cohort. According to the filtering criteria listed in the Methods and Materials, we identified homozygous variants in a novel gene *IQCN* (GenBank: NM_001145304.2) in two independent Han Chinese families (Fig 1A). Sanger sequencing confirmed that II-2 in family 1 carried a homozygous nonsense variant (M1: c.910C > T [p.Gln304Ter]) in *IQCN*, while each of the parents carried a heterozygous variant. Moreover, II-1 in family 2 carried a homozygous frameshift variant (M2: c.2453_2454del [p.Gln818Argfs*9]) (Fig 1A). The inheritance pattern in family 1 followed a recessive inheritance pattern, whereas that in family 2 was uncertain due to the parental DNA being unavailable.

Furthermore, the protein domains were predicted by SMART software, which showed that IQCN contained six IQ motifs (Fig 1B). Two variants were highly conserved and located ahead of the second IQ motif, leading to the loss of the other five IQ motifs in IQCN (Fig 1B). The allelic frequencies of the two variants were extremely low or absent in gnomAD and ExAC browsers (Table 1). Both *IQCN* variants were absent in our control database of 100 men with normal fertility. From the above results, we speculate that the homozygous variants in *IQCN* cause TFF and male infertility.

Disruption of IQCN led to TFF accompanied by defects in acrosome structure

Proband II-2 in family 1 underwent three cycles of ART treatment (Table 2). A total of 29 oocytes were obtained from the subject's partner, but none were successfully fertilized, even though donor oocytes were used in the third cycle and suffered TFF. Proband II-1 in family 2 underwent one ART treatment and suffered TFF after ICSI. Five mature oocytes from his partner were injected with his spermatozoa but failed to fertilize. The medical history and information derived from basic examinations of two men are listed in Appendix Table S1. By observing the polar body (PB) of unfertilized oocytes from the partner of II-2 in family 1, we found that the second PB failed to extrude (Fig 2A); this suggests that fertilization failure might be caused by the defects in oocyte activation. As PLCζ has been reported as the main sperm-borne oocyte activation factor (Swann, 2022), we detected the localization of PLCζ in the sperm from affected individuals. In normal spermatozoa, PLCζ is primarily located in the equatorial region of the sperm head (Fig 2B). However, PLCζ was abnormally located in the neck region of the mutant sperm (Fig 2B). This finding indicates that variants in *IQCN* lead to abnormal localization of PLCζ and deficiency of oocyte activation, resulting in TFF.

The semen analysis showed the affected individuals had normal sperm concentration and motility (Table 3). However, sperm morphology was assessed by H&E staining and transmission electron microscopy (TEM), and the percentage of sperm with normal morphology was 3.2 and 1.8%, respectively, which were lower than the reference limit (4%) according to the World Health Organization Standards, Fifth Edition (Cooper *et al*, 2010). Remarkably, two affected individuals showed a high proportion of sperm head anomalies, mainly manifested as a significantly higher proportion of tapered head and microcephalic head in two affected individuals (65.0 and 36.6%; 2.8 and 2.2%, respectively) and a higher proportion of thin head (40.5%) in II-1 in family 2 (Table 3 and Fig 2C).

To detect the ultrastructure of cephalic structures of spermatozoa, we analyzed the spermatozoa from II-2 in family 1 and the normal control by TEM. It was found that the acrosome structure of normal spermatozoa was tightly anchored to the nucleus and covered nearly half of the sperm head (Fig 2D). However, *IQCN* mutant spermatozoa showed head deformities, with a higher range of acrosome structure covered in the sperm head compared to that observed in normal sperm (79.5% ± 2.2% vs. 56.0% ± 1.3%) (Fig 2E). It is worth noting that the acrosome structure, especially the perinuclear ring, cannot be tightly anchored to the nucleus and exhibited a ruffled acrosome. Additionally, immunostaining analysis of the acrosome marker lectin (acrosomal outer membrane marker) showed that the acrosome signal in the affected individuals lost the cap-shaped structure (Fig 2B). These phenomena indicate that men harboring homozygous *IQCN* variants may suffer TFF accompanied by defects in the acrosome structure.

Iqcn-knockout (*Iqcn*$^{-/-}$) mice resembled abnormal acrosome structure and TFF phenotypes

To further explore whether the homozygous variants in *IQCN* were the pathogenic cause of TFF and abnormal acrosome structure, given that variants in exon 3 of *IQCN* lead to premature termination of translation, we generated a knockout mouse model with deletions of exons 3 and 4 in *IQCN* using CRISPR-Cas9 technology (Fig EV1A). Sanger sequencing of *Iqcn*$^{-/-}$ mice confirmed the presence of a 9,832-bp deletion in exons 3 and 4 (Fig EV1A) and homozygous mutated mice could be detected by specific primers through PCR (Fig EV1B). Compared with the expression level of *Iqcn*$^{-/-}$ in the testes of WT male mice, that in exons 3 and 4 was significantly reduced in the testes of *Iqcn*$^{-/-}$ male mice (Fig EV1C), indicating that exons 3 and 4 of *Iqcn* are totally deleted in homozygous mutated mice. For fertility assessment, *Iqcn*$^{-/-}$ male mice failed to produce offspring (Fig 3A), while *Iqcn*$^{-/-}$ female mice have normal fertility (Fig EV1D), suggesting that IQCN is exclusively indispensable for male fertility in mice.

To explore the cause of male infertility, we examined the *Iqcn*$^{-/-}$ male mice at the gross and histological levels. The testis weights and sizes showed no significant differences between WT and *Iqcn*$^{-/-}$ male mice (Fig 3B and C), nor were the spermatogenesis (Fig EV1E

 © 2022 The Authors

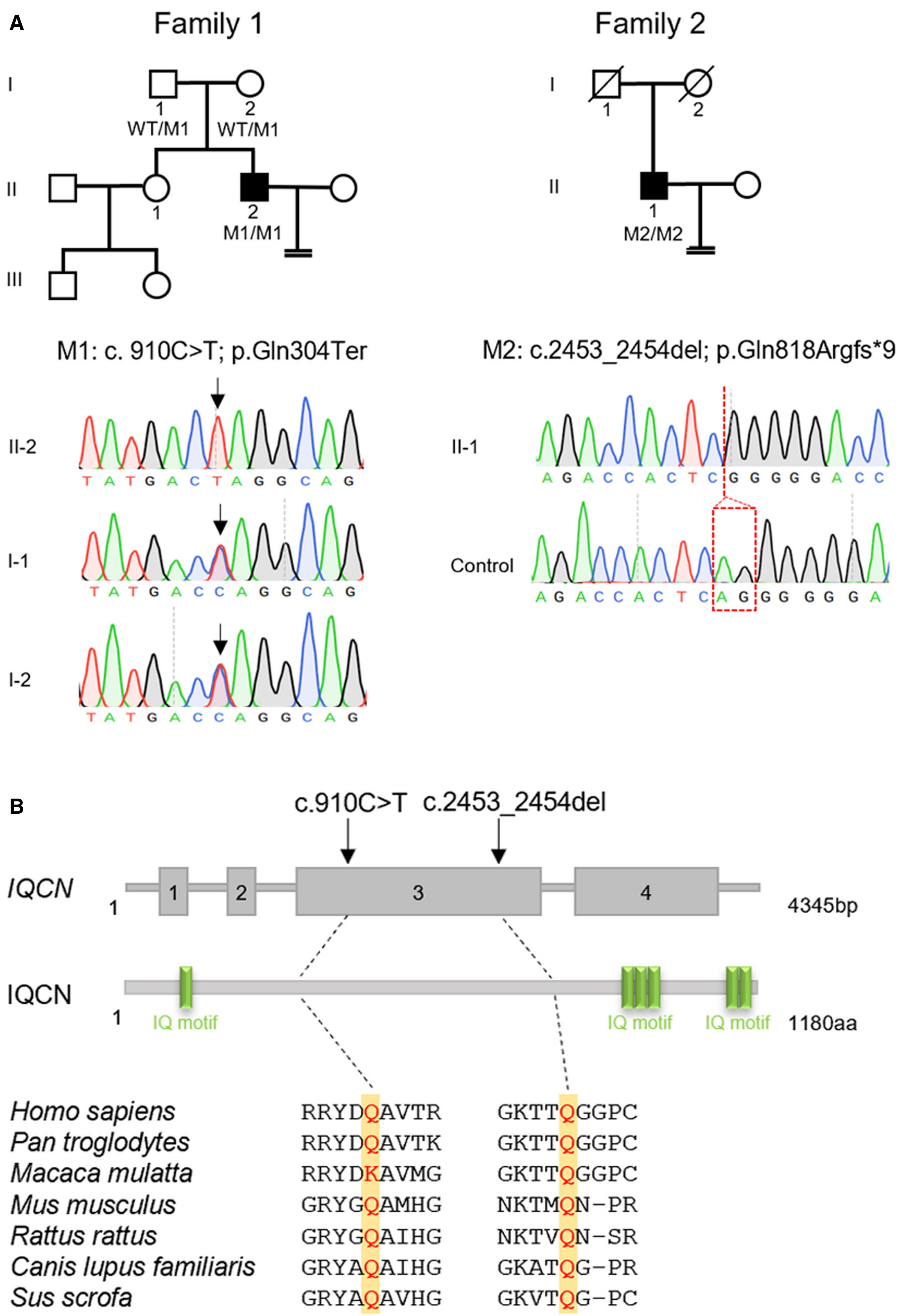
A
Family 1
Family 2
I
II
III
1
WT/M1
2
WT/M1
1
2
M1/M1
M2/M2
M1: c. 910C>T; p.Gln304Ter
M2: c.2453_2454del; p.Gln818Argfs*9
II-2
I-1
I-2
II-1
Control
B
c.910C>T c.2453_2454del
IQCN
1
2
3
4
4345bp
IQ motif
1180aa
Homo sapiens RRYDQAVTR GKTTQGGPC
Pan troglodytes RRYDQAVTK GKTTQGGPC
Macaca mulatta RRYDKAVMG GKTTQGGPC
Mus musculus GRYGQAMHG NKTMQN-PR
Rattus rattus GRYGQAIHG NKTVQN-SR
Canis lupus familiaris GRYAQAIHG GKATQG-PR
Sus scrofa GRYAQAVHG GKVTQG-PC

Figure 1.

◀ **Figure 1. Identification of pathogenic variants of *IQCN* in affected individuals with total fertilization failure (TFF).**

A Pedigree analysis of two families with TFF. The individuals (black squares) exhibited homozygous variants in *IQCN*; the confirmation by Sanger sequencing is shown below the pedigrees.

B Localization and conservation of mutant amino acids in IQCN. The yellow squares represent the localization of mutant amino acids in seven species.

Table 1. Overview of the *IQCN* pathogenic variants observed in the affected individuals.

Probands in families	Genomic position on chr19 (bp)	cDNA	Protein	Mutation type	SIFT[a]	MutTas[a]	gnomAD[b]	ExAC_EAS[b]
Family 1	18,377,440	c.910C > T	p.Gln304Ter	nonsense	—	D	N/A	N/A
Family 2	18,375,896	c.2453_2454del	p.Gln818Argfs*9	frameshift	—	D	0.0008	N/A

[a]Mutation assessment by SIFT and MutationTaster (MutTas). D, Prediction disease causing.
[b]Allelic frequency of corresponding mutations in the East Asian population according to the gnomAD and ExAC browsers.

Table 2. Clinical characteristics of IVF and ICSI attempts in affected individuals.

	Previous IVF and ICSI cycles						ICSI with AOA cycles					
Individuals	Insemination method	Total oocytes	Mature oocytes	Total fertilization Rate	Normal fertilization Rate	Good-quality embryos rate	Insemination method	Total oocytes	Mature oocytes	Total fertilization rate	Normal fertilization rate	Good-quality embryos rate
II-2 in Family 1	IVF+ Rescue ICSI	6	4	0% (0/4)	0% (0/4)	—— (0/0)	ICSI+AOA	4	3	66.7% (2/3)	66.7% (2/3)	50% (1/2)
	ICSI	6	5	0% (0/5)	0% (0/5)	—— (0/0)						
	ICSI Donor oocyte	17	10	0% (0/10)	0% (0/10)	—— (0/0)						
II-1 in Family 2	ICSI	6	5	0% (0/5)	0% (0/5)	—— (0/0)	ICSI+AOA	10	10	90% (9/10)	80% (8/10)	75% (6/8)

and F) and sperm counts (Fig 3D). As normal spermatozoa morphology is essential for fertilization and male fertility, we next determined the sperm morphology by H&E staining and TEM. Consistent with the clinical phenotypes of men with variants in *IQCN*, sperm from the cauda epididymis in *Iqcn*$^{-/-}$ male mice displayed a higher proportion of no sickle shape head than WT male mice by H&E staining (15.1% ± 0.5% vs. 4.7% ± 0.9%) (Fig 3E and F). To further investigate the ultrastructure of the sperm, we conducted TEM in sperm from the cauda epididymis. Discontinuous and loosely anchored acrosome structures were observed in the sperm of *Iqcn*$^{-/-}$ male mice (Fig 3G). Immunostaining analysis of lectin demonstrated a higher proportion of abnormal acrosomes in *Iqcn*$^{-/-}$ male mice compared with WT male mice (53.7% ± 0.9% vs. 8.0% ± 0.6%), revealing that the acrosome is not sickle-shaped and loosely anchored to the nuclear envelope (Fig 3H and I). In the acrosome reaction (AR) assay, the percentages of sperm with spontaneous and induced AR were not significantly different between spermatozoa from WT and *Iqcn*$^{-/-}$ male mice (Fig 3J).

Subsequently, we observed the fertilization and embryonic development potential by ICSI. We found that sperm from *Iqcn*$^{-/-}$ mice exhibited TFF and even had no 2-cell embryo formation compared with WT mice (Fig 4A and B). To further investigate the oocyte activating capacity of sperm after ICSI, oocytes from WT females were injected with WT and *Iqcn*$^{-/-}$ sperm to monitor Ca^{2+} oscillations. As a result, 8–9 Ca^{2+} spikes were observed in oocytes injected with WT sperm at 3-h postinjection, but the spikes were absent in oocytes injected with *Iqcn*$^{-/-}$ sperm (Fig 4C). As it observed the absence of calcium oscillations in oocytes, PLCζ is the main factor to activate calcium oscillations. We evaluated PLCζ status in the oocyte by injecting with WT and *Iqcn*$^{-/-}$ spermatozoa. In the oocyte injected with WT spermatozoa, PLCζ diffused into the ooplasm, and the oocytes exited to metaphase II (MII) arrest and entered the second anaphase of meiosis (Fig 4D). However, in the oocyte injected with *Iqcn*$^{-/-}$ spermatozoa, the oocyte remained in MII stage, the spermatozoa head changed into premature chromosomal condensation (PCC) status, and the PLCζ signal was absent in the ooplasm (Fig 4D). These phenomena imply that *Iqcn*$^{-/-}$ mice had a phenotype that resembled TFF due to oocyte activation factor deficiency.

Sperm-borne oocyte activation factor PLCζ was lost during acrosomal biogenesis

Since PLCζ is absent in oocytes, we therefore investigated the status of PLCζ in sperm and spermatids. It found that PLCζ was mainly located in the acrosomal region of the sperm head in epididymal spermatozoa from WT male mice (Fig 4E). However, PLCζ was absent in epididymal sperm from *Iqcn*$^{-/-}$ male mice (Fig 4E). We then detected the localization of PLCζ during acrosomal biogenesis. In the testes of WT male mice, PLCζ was localized in the acroplaxome from the Golgi phase to the maturation phase, which was tightly localized between the acrosome and the nuclear envelope with the appearance of the acrosome (Fig 4F). During the acrosomal biogenesis of *Iqcn*$^{-/-}$ male mice, PLCζ was normally localized in the acroplaxome in the Golgi phase (steps 1–3) (Fig 4F). However, in the cap phase (steps 4–7) and acrosome phase (steps 8–12), PLCζ was only localized to the tip of the head, not the region covered by

© 2022 The Authors

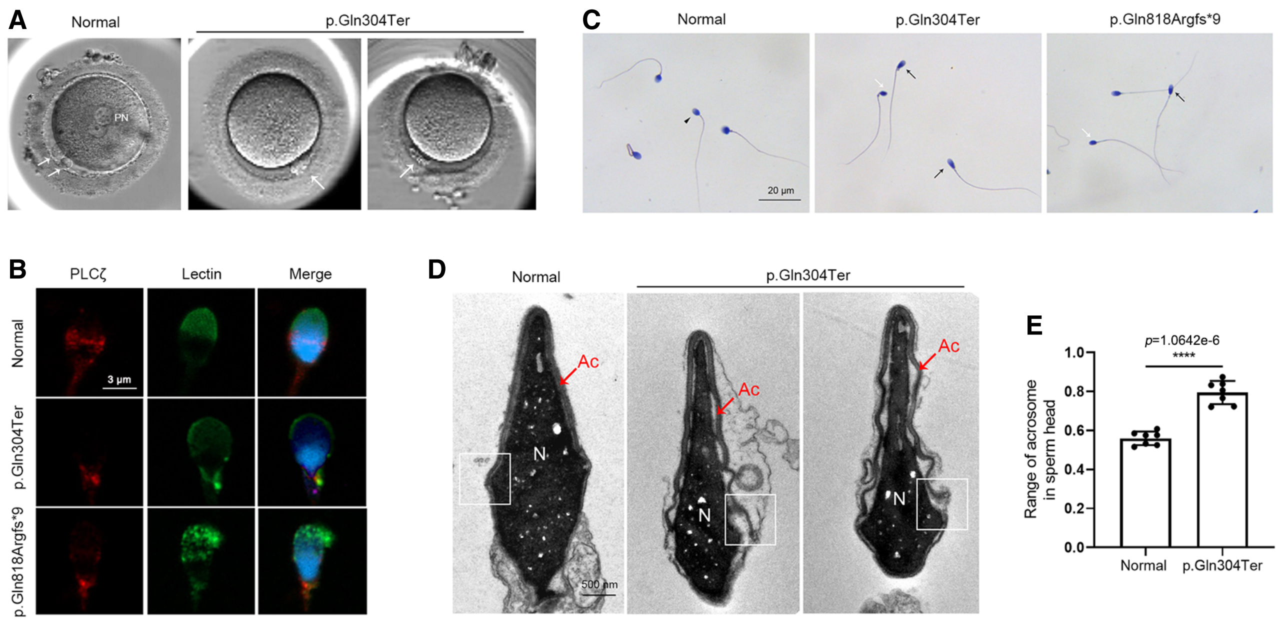

Figure 2. Affected individuals with variants in *IQCN* exhibited TFF and defects in the acrosomal ultrastructure.

A The unfertilized oocytes were injected with spermatozoa with a variant in *IQCN* at 15–18 h postinjection. The white arrowheads represent the polar body (PB). PN represents pronucleus.

B The expression and localization of PLCζ in sperm from the affected individuals. Scale bars, 3 µm.

C H&E staining of spermatozoa from affected individuals. The black arrow represents normal spermatozoa. The black arrowheads represent tapered-head spermatozoa with increased major axis length and normal minor axis length. The white arrowheads represent thin spermatozoa with decreased major axis length and minor axis length. Scale bars, 20 µm.

D The ultrastructure of spermatozoa from normal and affected individual. The white squares represent the perinuclear ring. Ac represents acrosome. N represents nucleus. Scale bars, 500 nm.

E The range of acrosomes in sperm heads from normal and affected individuals ($n = 7$).

Data information: Data represent mean ± SEM. ****$P < 0.0001$ by unpaired Student's *t*-test (E).
Source data are available online for this figure.

the entire acrosome (Fig 4F). In the maturation phase (steps 13–16), PLCζ was lost from mature spermatozoa (Fig 4F).

IQCN is essential for manchette assembly in elongating spermatids

To further focus on the mechanisms of abnormal acrosome structure and TFF, we explored the function of IQCN during spermiogenesis. To this end, we performed immunostaining analysis of IQCN and α-tubulin of manchette microtubules in the testis and found that IQCN was expressed in elongating spermatids (steps 8–14) during spermiogenesis and was co-localized with α-tubulin (Fig 5A), which indicates that IQCN was close to the manchette structure in elongating spermatids. We next determined the ultrastructure of the manchette of testes using TEM (Fig 5B). Elongating spermatids in WT mice showed initiation of manchette microtubule formation near the perinuclear ring at step 8, as well as thinning of the cytoplasm and a gradual orientation of the acrosome toward the overlying plasma membrane in WT spermatids. Then, the manchette was elongated so that the acrosome surrounded approximately half of the sperm head at steps 13–14 in WT spermatids. Subsequently, the manchette was degraded, which was accompanied by the expulsion of cytoplasmic droplets at steps 15–16 (Fig 5B). However, in *Iqcn*$^{-/-}$ mice, the manchette was ectopically present near the perinuclear ring at steps 8–12 and excessively elongated at steps 13–14, resulting in the complete encirclement of the sperm head by the acrosome. Moreover, cytoplasmic droplets failed to extrude at steps 15–16 (Fig 5B). This phenomenon was confirmed by immunostaining of α-tubulin, which showed that the length of the manchette structure in *Iqcn*$^{-/-}$ mice was significantly longer than that in WT mice (8.6 ± 0.3 µm vs. 4.7 ± 0.2 µm), and was accompanied by an aberrant nucleation site in spermatids at steps 13–14 (Fig 5C and D). The results suggest that the disruption of IQCN led to a defective manchette structure, which resulted in head deformity with aberrant acrosome in epididymal sperm.

IQCN interacts with calmodulin to facilitate manchette assembly

Previous studies have shown that calmodulin plays an important role in sperm function and proteins containing IQ motifs can bind to calmodulin (Martin Bahler, 2002). Here, we first investigated the localization of calmodulin during spermiogenesis by immunostaining. The results showed that in the elongating spermatids, calmodulin was co-localized with α-tubulin at steps 8–14 in WT mice. However, in *Iqcn*$^{-/-}$ mice, calmodulin was only partially co-localized with α-tubulin (Fig 6A). Additionally, we found that IQCN was co-immunoprecipitated with calmodulin in the testes from WT mice. However, IQCN lost the interaction with calmodulin in the

© 2022 The Authors

Table 3. Semen parameters and sperm morphology analyses of the affected individuals

	II-2 in Family 1	II-1 in Family 2	Low reference limit
Semen parameters			
Semen volume (ml)	1.6	3.5	1.5[a]
Sperm concentration (10^6/ml)	281.9	99.8	15[a]
Total motility (%)	47.9	69.0	40[a]
Sperm morphology			
Normal spermatozoa (%)	3.2	1.8	4[a]
Tapered head (%)	65.0[c]	36.6[c]	3[b]
Thin head (%)	2.0	40.5[c]	14[b]
Macrocephalic (%)	4.4	1.3	7[b]
Microcephalic (%)	2.8[c]	2.2[c]	1[b]
Multiple heads (%)	0.0	0.0	2[b]
Abnormal postacrosomal region (%)	9.6	6.6	42[b]
Abnormal acrosomal region (%)	6.0	2.6	60[b]

[a]Reference limits according to the World Health Organization Standards, Fifth Edition (Cooper *et al*, 2010).
[b]Reference limits according to the distribution range of morphologically normal spermatozoa observed in 926 fertile individuals (Auger *et al*, 2016).
[c]Abnormal values.

testes from *Iqcn*$^{-/-}$ mice (Fig 6B). These findings suggest that the IQCN–calmodulin complex participates in manchette-related functions during spermiogenesis.

To further explore how IQCN affects calmodulin and thus regulates microtubule assembly, we performed *IQCN* (WT, M1, and M2) and *CALM3* transfection in HeLa cells. Following transfection, calmodulin was co-localized with α-tubulin in the mitotic apparatus (Fig 6C). The expression and localization of calmodulin showed no significant difference between the WT and mutant *IQCN* transfection groups, but the percentage of multipolar spindles increased significantly in the mutant *IQCN* transfection groups (Fig 6C and D). Furthermore, we added the calmodulin antagonist W-7 when transfecting WT + oe-*CALM3*. The results showed that compared with the WT + oe-*CALM3* group, the percentage of multipolar spindles in the group treated with W-7 increased significantly, which was similar to that in the mutant *IQCN* transfection groups (Fig 6C and D). These results suggest that IQCN is an upstream molecule of calmodulin that can affect microtubule assembly.

Calmodulin-binding proteins are altered in the *Iqcn*$^{-/-}$ mouse model

Since we have confirmed the interaction and regulating relationship of IQCN and calmodulin, to further explore the alteration of calmodulin-binding proteins (CaMBPs) when calmodulin no longer interacts with IQCN, we performed immunoprecipitation followed by LC–MS to analyze the differentially expressed CaMBPs in the testes of WT and *Iqcn*$^{-/-}$ mice (Fig 7A). The results of data quality control displayed a high correlation between the two groups of replicates (Fig EV2A and B). IQCN and three other known manchette-related proteins, LZTFL1, KIF27, and RSPH6A, were exclusively detected with high LFQ intensities in the WT group (Fig 7B). After normalization of raw data, 624 differentially expressed proteins (DEPs; fold change [FC] >1.5) were identified between WT and *Iqcn*$^{-/-}$ testes, with more downregulated proteins (457, 73.2%) (Appendix Table S2) than upregulated proteins (168, 26.9%); the trend is consistent under each distribution of fold changes (Fig 7C). Gene ontology (GO) analysis revealed downregulated proteins substantially enriched in the cytoskeleton assembly pathway, such as microtubule-based movement (GO: 0007018), negative regulation of cytoskeleton organization (GO: 0051494), actin cytoskeleton organization (GO: 0030036), and microtubule bundle formation (GO: 0001578), as the top 15 enriched biological processes (BPs) (Fig 7D). We further investigated the predicted regulatory networks between cytoskeleton-associated DEPs. The results showed that they were roughly divided into three groups, with clustering of two gene families, including IFT family proteins and its motor protein (IFT22, IFT43, IFT74, IFT81, IFT140, IFT172, WDR19, TTC21B, and DYNC2H1) and ribosomal protein family (RPS5, RPS25, RPS27, and RPSA) (Fig 7E). We selected two IFT family proteins (IFT74 and IFT81) for further immunoblot verification and found the trend of change is completely consistent with that of LC–MS data (Fig EV2C).

IQCN-associated male infertility could be rescued by ICSI with assisted oocyte activation

ICSI with AOA with Ca^{2+} ionophore A23187 was a technique that has been clinically applied for TFF or low fertilization rate in ART (Bonte *et al*, 2019; Li *et al*, 2019). A series of studies have demonstrated the effectiveness and safety of this technique (Yeste *et al*, 2016; Shan *et al*, 2021; Kashir *et al*, 2022). Because *Iqcn*$^{-/-}$ sperm failed to induce Ca^{2+} oscillations in oocytes, we explored whether *Iqcn*-associated TFF could be rescued via ICSI with AOA by Ca^{2+} ionophore exposure. In *Iqcn*$^{-/-}$ mice, 79.1% ± 4.9% of oocytes achieved successful fertilization and 68.0% ± 6.2% of oocytes developed into 2-cell embryos, and 39.4% ± 1.6% of embryos developed into blastocysts (Fig EV3).

The affected individuals volunteered to attempt this method and gave their informed consent. After mutation screening for the partners of the individuals to exclude rare pathogenic variants in *IQCN*, oocytes were injected with *IQCN* mutant sperm. As a result, 66.7% and 80% of oocytes achieved successful normal fertilization, then 50.0% and 75.0% of zygotes developed into good-quality blastocyst embryos, respectively (Table 2). After embryo selection, we transferred two blastocysts on day 5, and two healthy babies were born in family 2. These results suggest that *IQCN*-associated infertility can be successfully overcome with ICSI with AOA.

Discussion

In this study, we first identified homozygous pathogenic variants in a novel gene *IQCN* from 42 men with TFF after ICSI. In *Iqcn*$^{-/-}$ male mice, mutant IQCN lost the interaction with calmodulin and disrupted the assembly of the manchette, resulting in sperm head deformity and abnormal localization of PLCζ. Fortunately, we confirmed that ICSI with AOA can successfully overcome TFF in humans and mice.

 © 2022 The Authors

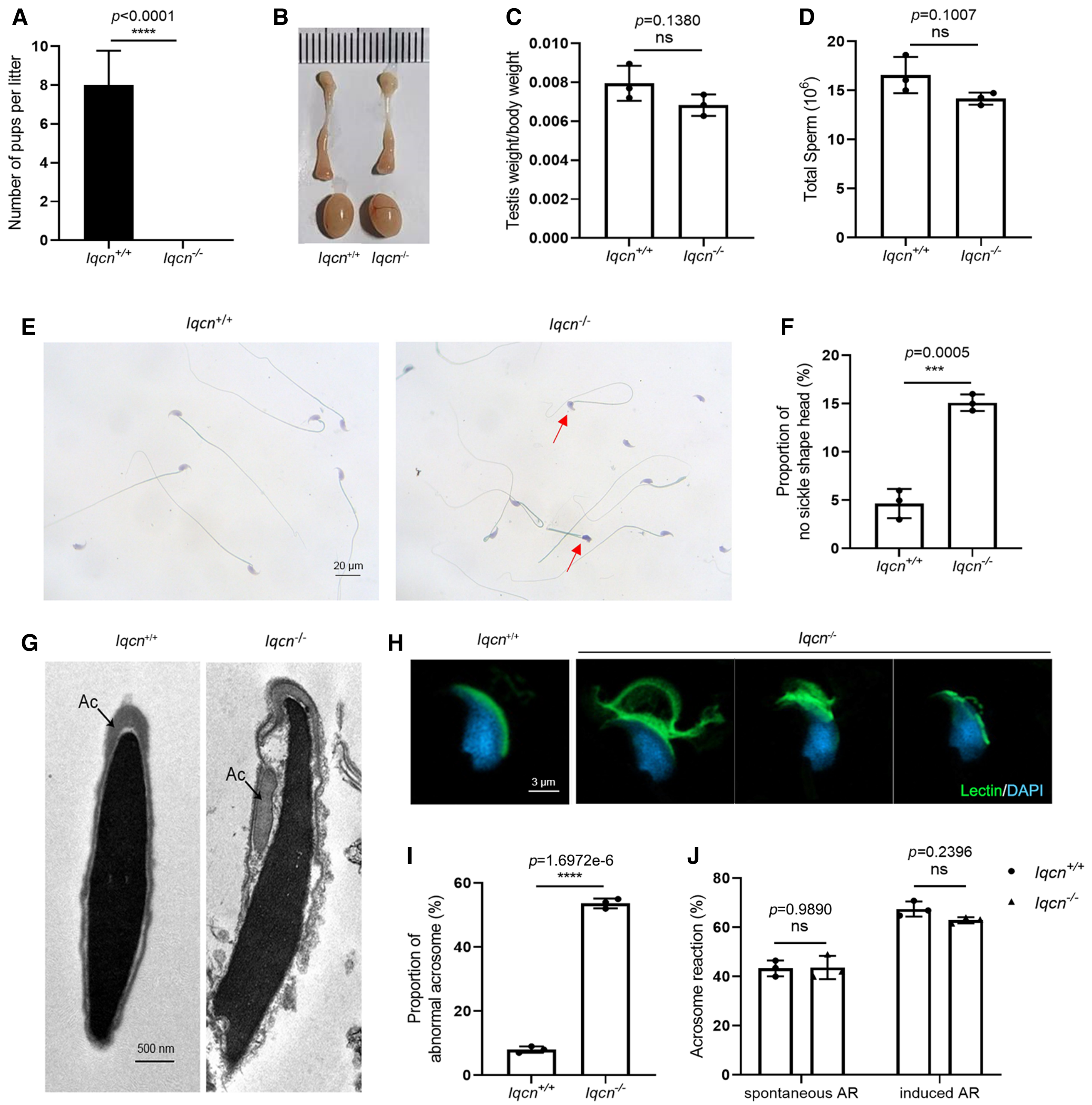

Figure 3. Knockout of *Iqcn* leads to male infertility and acrosomal defects in a mouse model.

A Fertility assessment experiments in WT and *Iqcn*$^{-/-}$ male mice after mating with WT female mice (n = 18).

B Size of the testes and epididymides in WT and *Iqcn*$^{-/-}$ male mice.

C Quantification ratio of the testis weight/body weight in WT and *Iqcn*$^{-/-}$ male mice (n = 3).

D Sperm counts in the caudal epididymis from WT and *Iqcn*$^{-/-}$ male mice (n = 3).

E H&E staining of sperm from WT and *Iqcn*$^{-/-}$ male mice. Red arrowheads represent sperm with no sickle shape heads. Scale bars, 20 μm.

F Percentage of sperm without sickle shape heads from WT and *Iqcn*$^{-/-}$ male mice (n = 3).

G Ultrastructure of spermatozoa from WT and *Iqcn*$^{-/-}$ male mice. Ac represents the acrosome. Scale bars, 500 nm.

H Immunostaining of lectin (green) in spermatozoa from WT and *Iqcn*$^{-/-}$ male mice. Scale bars, 3 μm.

I Percentage of abnormal acrosomes in spermatozoa from WT and *Iqcn*$^{-/-}$ male mice (n = 3).

J Percentage of spontaneous and induced acrosome reactions in spermatozoa from WT and *Iqcn*$^{-/-}$ male mice (n = 3).

Data information: Data represent mean ± SEM. ***P < 0.001, ****P < 0.0001, ns represents no significance by the unpaired Student's t-test (A, C, D, F and I), or by one-way ANOVA (J).
Source data are available online for this figure.

© 2022 The Authors

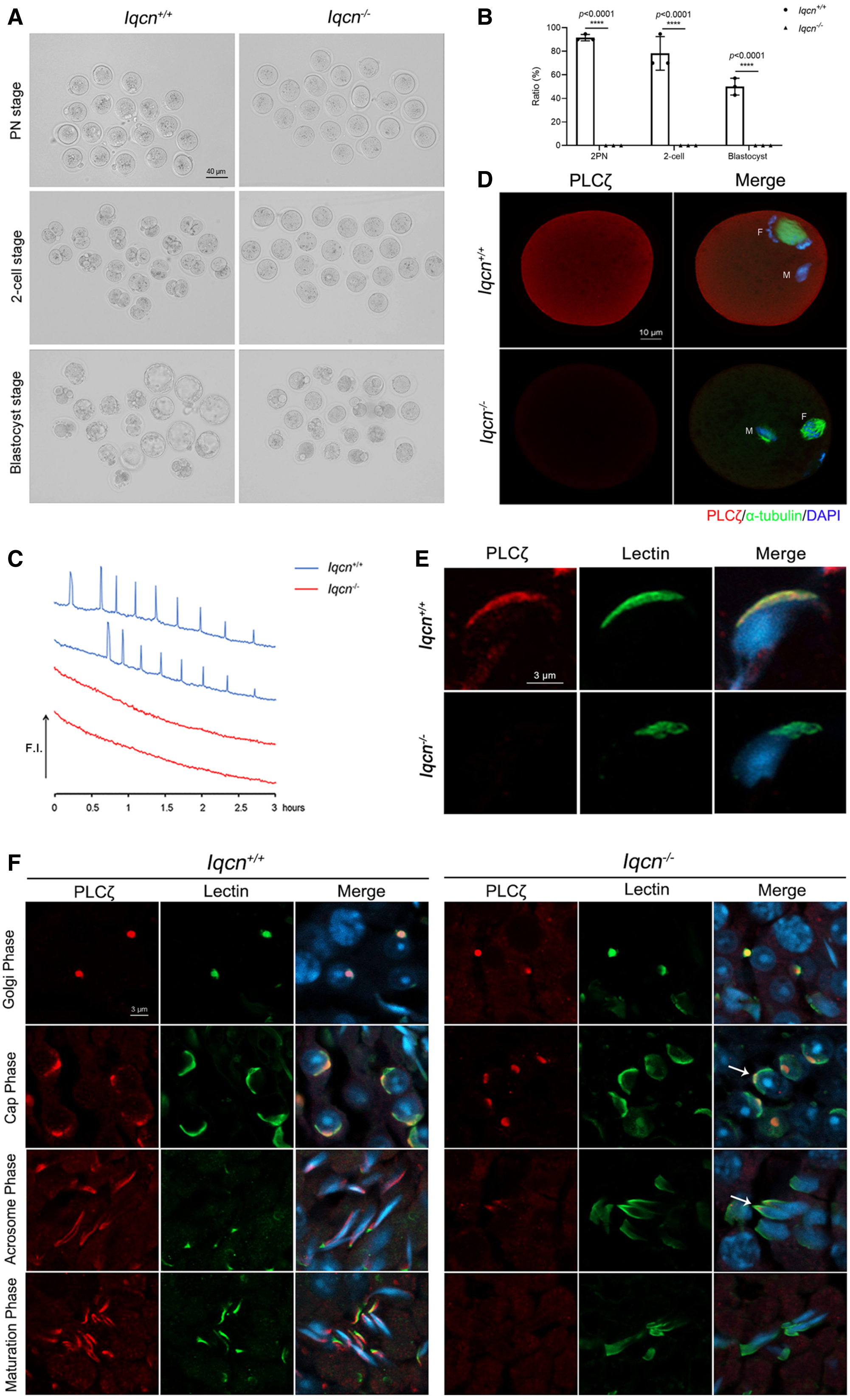

Figure 4.

 © 2022 The Authors

Figure 4. $Iqcn^{-/-}$ male mice induce TFF with oocyte activation failure.

A Representative two pronucleus (2PN) zygotes, two-cell embryos, and blastocysts after ICSI in WT and $Iqcn^{-/-}$ male mice. Scale bars, 40 μm.
B Percentage of 2PN zygotes, two-cell embryos, and blastocysts after ICSI in WT and $Iqcn^{-/-}$ male mice ($n = 3$).
C Profile of Ca^{2+} responses induced by oocyte injection with spermatozoa from WT and $Iqcn^{-/-}$ male mice ($n = 5$). Spermatozoa from WT male mice induced 8–9 Ca^{2+} spikes over a 3-h period (blue line), while spermatozoa from $Iqcn^{-/-}$ male mice failed to induce spikes (red line).
D Immunostaining of oocytes injected with spermatozoa from WT and $Iqcn^{-/-}$ male mice. The expression of PLCζ (red) and α-tubulin (green) was detected in oocytes. M represents male nucleus and F represents the female nucleus. Scale bars, 10 μm.
E Expression and localization of PLCζ in sperm from WT and $Iqcn^{-/-}$ male mice. Scale bars, 3 μm.
F Expression and localization of PLCζ during acrosomal biogenesis from WT and $Iqcn^{-/-}$ male mice. PLCζ was localized in the acroplaxome in WT spermatids. PLCζ was only localized in the tip of the head in the cap and acrosome phases (white arrowheads) and was lost during the maturation phase in $Iqcn^{-/-}$ mice. Scale bars, 3 μm.

Data information: Data represent mean ± SEM. ****$P < 0.0001$ by one-way ANOVA (B).
Source data are available online for this figure.

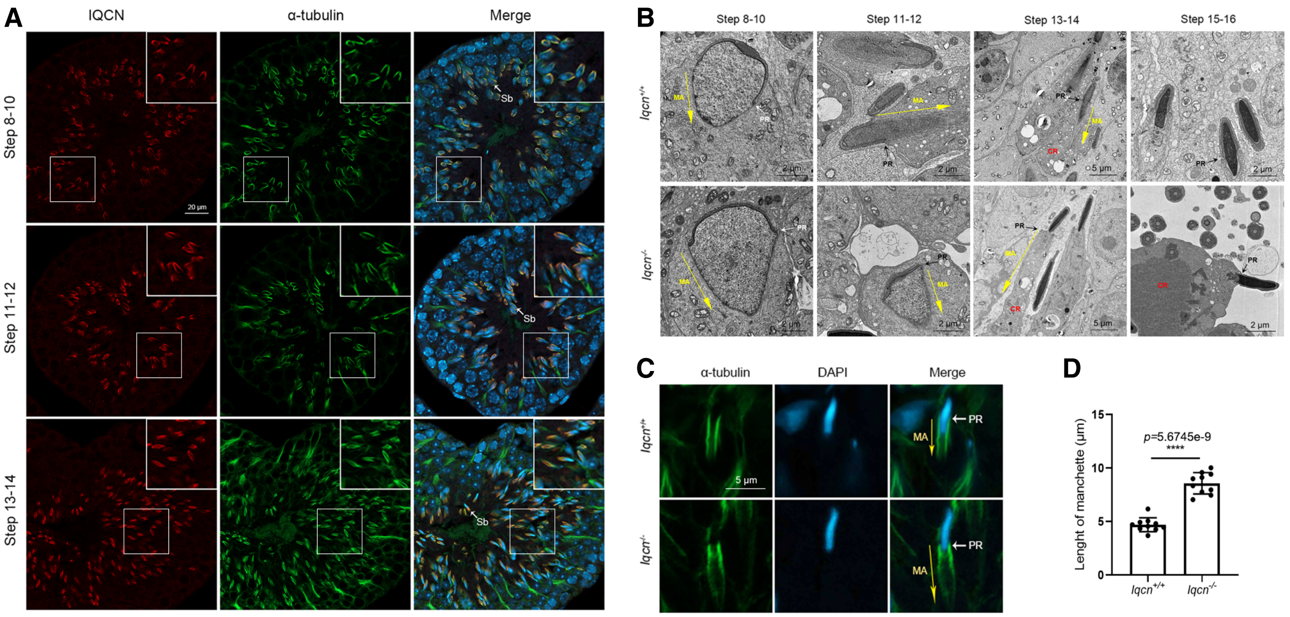

Figure 5. $Iqcn^{-/-}$ male mice cause defects in manchette assembly during spermiogenesis.

A Expression and localization of IQCN during spermiogenesis in the testes of WT male mice. IQCN (red) was co-localized with α-tubulin (green) from steps 8–14. Sb represents late spermatids. Scale bars, 20 μm.
B Ultrastructure of the testes from WT and $Iqcn^{-/-}$ male mice from steps 8–16. MA represents manchette. PR represents perinuclear ring. CD represents cytoplasmic droplets. Scale bars, 2 μm (steps 8–12; 15–16) and 5 μm (steps 13–14).
C Immunostaining of α-tubulin (green) in spermatids at steps 13–14. MA represents manchette. PR represents perinuclear ring. Scale bars, 5 μm.
D Length of the manchette structure in spermatids at steps 13–14 from WT and $Iqcn^{-/-}$ male mice ($n = 10$).

Data information: Data represent mean ± SEM. ****$P < 0.0001$ by unpaired Student's *t*-test (D).
Source data are available online for this figure.

The protein domain encoded by IQCN contains six IQ motifs, and the IQCN variant here resulted in the loss of five IQ motifs (Fig 1B). Previous studies have clarified the functions of another two IQ motif-containing proteins, IQCF1 and IQCG, based on knockout mouse models, both of which are highly expressed in testicular tissue and participate in spermatogenesis (Li *et al*, 2014; Fang *et al*, 2015). $Iqcf1^{-/-}$ mice showed sperm motility decreases and acrosome reaction deficiency (Fang *et al*, 2015), while $Iqcg^{-/-}$ mice showed microtubule disorder, flagellum dysfunction, and sperm head deformity (Li *et al*, 2014); however, neither of them had fertilization disorders. We confirmed that IQCN was localized in the manchette and persisted in the elongation stage of steps 8–14 during spermatogenesis. Furthermore, $Iqcn^{-/-}$ showed disordered manchette assembly, resulting in an abnormal acrosome structure and TFF. Combined with previous studies, we suggest that the functions of IQ motif-containing genes are different during spermiogenesis. In our study, men carried *IQCN* variants did not exhibit the sperm motility impairments seen in the $Iqcg^{-/-}$ and $Iqcf1^{-/-}$ mice, nor did the abnormal acrosome reaction as in the $Iqcf1^{-/-}$ mice (Table 3 and Fig 3J). We herein correlated the inner relationship between manchette assembly disorder and TFF for the first time. We speculate that the abnormal localization of PLCζ was due to the abnormal assembly of manchette by variants in *IQCN*, leading to the abnormal localization of PLCζ in the acroplaxome. In this situation,

© 2022 The Authors

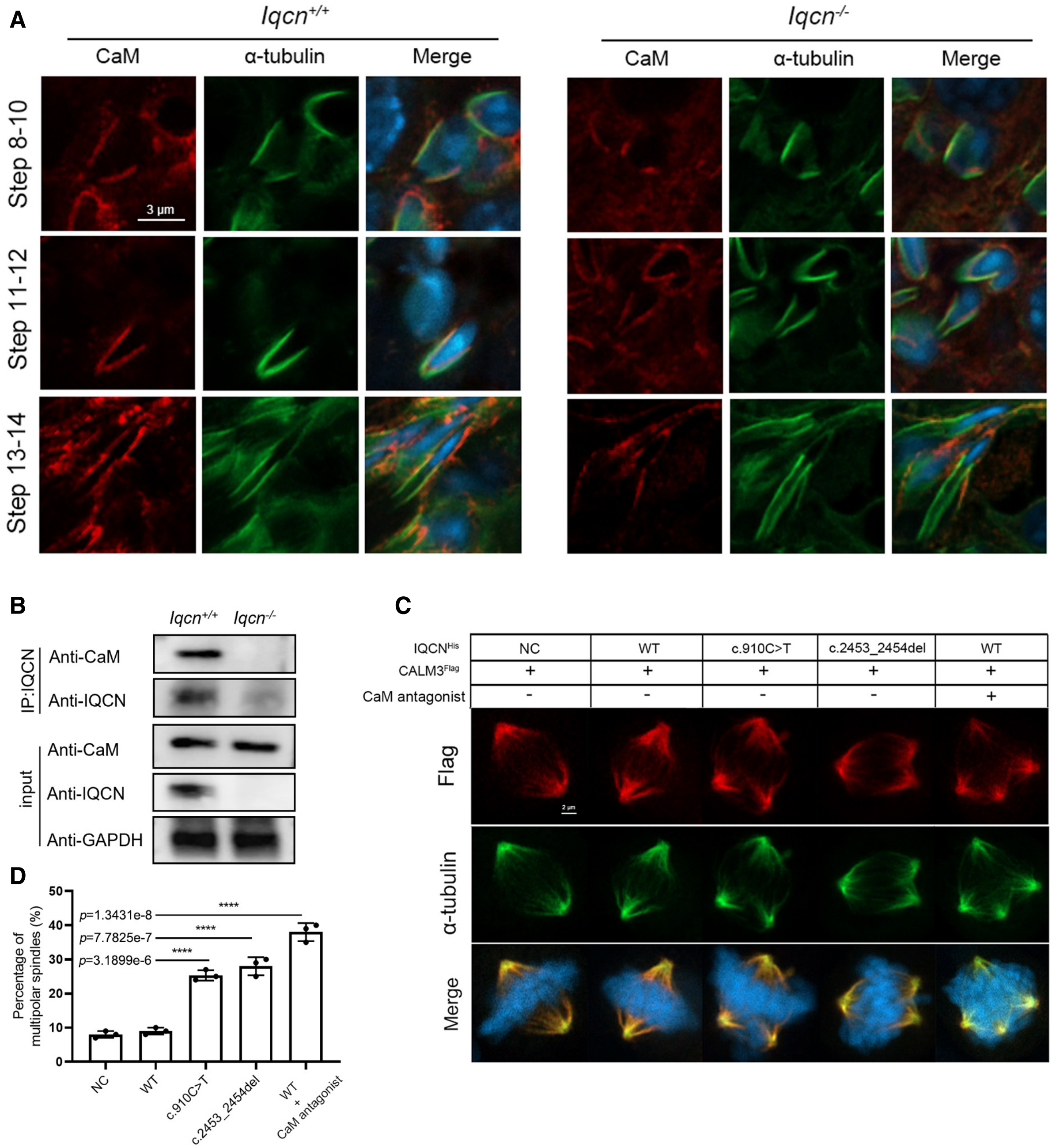

Figure 6. IQCN interacts with calmodulin to participate in manchette assembly.

A Expression and localization of calmodulin during spermiogenesis in the testes of WT and *Iqcn*$^{-/-}$ male mice. Calmodulin (red) was co-localized with α-tubulin (green) from steps 8 to 14 in WT mice but failed to co-localize in steps 13–14 in *Iqcn*$^{-/-}$ male mice. Scale bars, 3 μm.

B Interaction between IQCN and calmodulin (CaM) following co-IP by IQCN antibody in testes from WT and *Iqcn*$^{-/-}$ male mice ($n = 3$). IQCN interacts with calmodulin in WT mice, the interaction of which is completely lost in *Iqcn*$^{-/-}$ male mice. GAPDH was used as the internal control.

C Immunostaining of calmodulin (red) and α-tubulin (green) in HeLa cells. The normal (WT) and mutant (c.910C > T and c.2453_2454del) plasmids of *IQCN* were transfected into HeLa cells. HeLa cells were treated with an antagonist of calmodulin W-7. Multipolar spindles were observed in HeLa cells transfected with mutant plasmids and W-7 treatment. Scale bars, 2 μm.

D Percentage of multipolar spindles in WT and mutant IQCN transfected HeLa cells. ($n = 3$ biological replicates).

Data information: Data represent mean ± SEM. ****$P < 0.0001$ by one-way ANOVA (D).
Source data are available online for this figure.

© 2022 The Authors

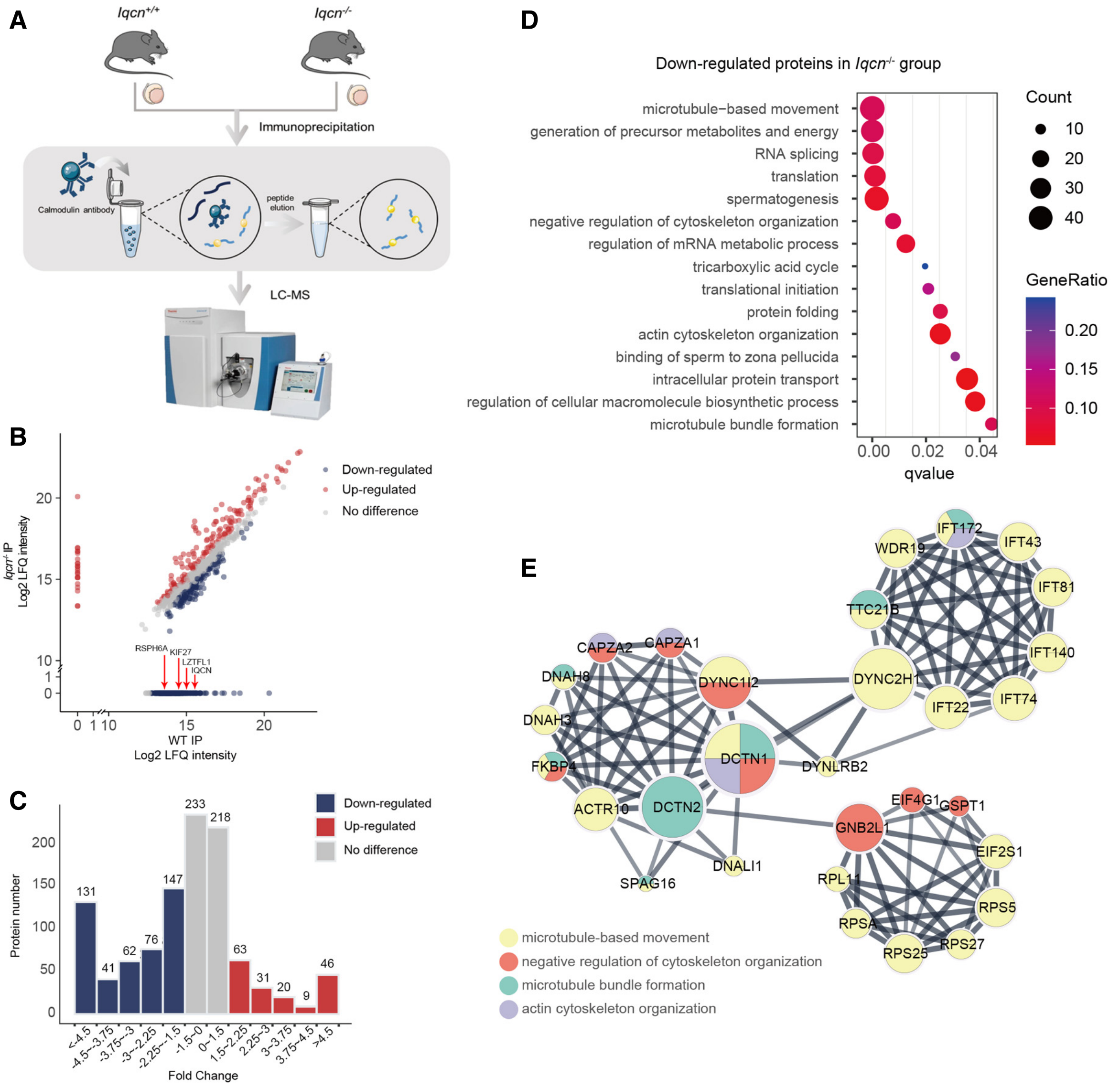

Figure 7. Potential downstream molecular changes in calmodulin regulated by IQCN.

A Workflow of immunoprecipitation (IP) followed by LC–MS analysis of the testes in WT and *Iqcn*$^{-/-}$ male mice.

B Mass spectrometry results from *Iqcn*$^{-/-}$ IP and WT IP ($n = 2$). Red arrowheads represent the IQCN and manchette-related proteins exclusively detected in the WT group. Upregulated DEPs are shown in red; downregulated DEPs are shown in blue.

C Distribution and counts of up- and downregulated DEPs. Upregulated DEPs are shown in red. Downregulated DEPs are shown in blue.

D GO term enrichment analyses of downregulated DEPs.

E Protein–protein interaction network of downregulated DEPs enriched in microtubule-related processes. Yellow dots represent microtubule-based movement process; red dots represent negative regulation of the cytoskeleton organization process; green dots represent the microtubule bundle formation process; and purple dots represent the actin cytoskeleton organization process.

the oocyte failed to be activated, and sperm chromatin changed into PCC because of the presence of meiosis promoting factor (Schmiady *et al*, 1996).

The IQ motif has been shown to interact with calmodulin, which is a small, highly conserved, 148 amino acid-long acidic protein (Marshall *et al*, 2015). Calmodulin recruits related CaMBPs and plays an important role in sperm motility, capacitation, acrosome reaction, and sperm egg membrane fusion (Schlingmann *et al*, 2007; Navarrete *et al*, 2015). However, the functions of calmodulin during spermiogenesis are largely unknown. It has only been reported that calmodulin is accumulated in the subacrosomal layer of elongating spermatids and the perinuclear ring, while, during the maturation phase of spermatids, it is located in the postacrosomal layer (Kann *et al*, 1991). From these phenomena, we speculate that calmodulin plays an important role in manchette development and regulation of the subacrosomal actin aggregation state during spermiogenesis. In our study, we confirmed that IQCN affects the assembly of the manchette structure via interacting with calmodulin. In the

© 2022 The Authors

in vitro study, we confirmed a high incidence of multipolar spindles in mutant *IQCN* transfected cells, which was reproduced after calmodulin antagonist W-7 treatment. The appearance of multipolar spindles revealed defects in the microtubule organizing center (MTOC) during nucleation. During spermiogenesis, the appearance of microtubules first to the perinuclear ring might function as MTOC. Some reports suggested that the perinuclear ring serves as a possible nucleation site (Yoshida *et al*, 1994). Interestingly, we also observed that the appearance site of microtubules in spermatids of *Iqcn*$^{-/-}$ was closer to the sperm neck and not tightly anchored to the nucleus, which indicates a deficiency of nucleation during spermiogenesis. Because of the differences between cells and spermatids, it is difficult to obtain a completely consistent phenotype. However, combined with the above results, we can speculate that the abnormality of IQCN is caused by the nucleation deficiency by interacting with calmodulin.

Furthermore, we performed IP followed by LC–MS. We identified IQCN, and the other three manchette structure proteins were detected in the WT group and absent in the *Iqcn*$^{-/-}$ group. Among these, the LZTFL1 had the highest protein abundance in the WT testes. A previous study indicated that LZTFL1 is present in the developing flagella, close to the manchette (Huang *et al*, 2021). *Lztfl1*-knockout mice show a high level of abnormal sperm and reduced fertilization ability, which is similar to the phenotypes we observed (Huang *et al*, 2021). As is known, proteins are transported through the manchette by intramanchette transport (IMT) to the base of the sperm tail by IFT (Lehti & Sironen, 2016). From GO analysis, a group of IFT proteins was found to be downregulated in the *Iqcn*$^{-/-}$ group. All seven IFT proteins were predominantly expressed in mouse testes, with increasing abundance during spermatogenesis (Fig EV4). Additionally, deficiency in IFT74, IFT81, IFT140, and IFT172 have been proven to be related to spermiogenesis defects and male infertility (Shi *et al*, 2019; Wang *et al*, 2019; Qu *et al*, 2020; Zhang *et al*, 2020). Above, we speculated that variants in IQCN may disturb the function of IFT-related proteins via calmodulin regulation, and found that ribosomal protein family members are downregulated under IQCN knockout. A previous study suggested that ribosomal protein loss is associated with microtubule-based defects (Jang *et al*, 2021); however, the exact regulatory mechanism and the role in spermiogenesis require further investigation.

Presently, ART has expanded opportunities for infertile couples. In previous studies, most cases of TFF caused by a deficiency of PLCζ could be overcome by ICSI with AOA (Dai *et al*, 2020), which implies that PLCζ could become a molecular marker for judging male fertilization ability. Our findings demonstrate that ICSI with AOA could overcome IQCN-associated TFF in humans and mice, which provided an effective clinical intervention. The dynamic changes of PLCζ during capacitation and acrosome reaction have been reported (Grasa *et al*, 2008; Mejia-Flores *et al*, 2017). However, the specific mechanism has not been elucidated. Previous studies reported that actin-like family members, *ACTL9* and *ACTL7A*, were localized in the arcoplaxome and maintained the precise localization of PLCζ (Xin *et al*, 2020; Dai *et al*, 2021). In this study, the abnormal localization of PLCζ was caused by defects in manchette assembly. Above all, we established a relationship between the dynamic changes of PLCζ and acrosomal biogenesis. However, the exact molecular mechanism needs further investigation.

In conclusion, our study documented a novel gene, *IQCN*, and demonstrated that IQCN is crucial to fertilization and male fertility, especially by regulating manchette assembly to affect sperm head formation, resulting in the oocyte activation deficiency. These results will provide new insights into the mechanism of spermatogenesis, and inform decision-making and appropriate genetic counseling of male-source TFF.

Materials and Methods

Study subjects

We recruited 42 infertile men from the Reproductive and Genetic Hospital of CITIC-Xiangya from January 2014 to May 2022. The study of human and animal subjects was approved by the Ethics Committee of the Reproductive and Genetic Hospital of CITIC-Xiangya (LL-SC-2017-009) and the Animal Welfare Ethics Committee of Central South University (XMSB-2022-0057), respectively. Informed consent was obtained from all subjects and the experiments conformed to the principles set out in the WMA Declaration of Helsinki and the Department of Health and Human Services Belmont Report.

The inclusion criteria were as follows: (i) men with primary infertility; (ii) ≥ one ICSI attempt; (iii) ≥ five MII oocytes retrieved in a single attempt; (iv) TFF or poor fertilization (fertilization rate < 20%); (v) male source based on the mouse oocyte activation test as previously described (Heindryckx *et al*, 2005, 2008). The exclusion criterion was men with globozoospermia.

Whole-exome sequencing (WES)

Genomic DNA (gDNA) was isolated from peripheral blood samples of the affected men using the QIAamp DNA blood mini kit (Qiagen, 51,106). The gDNA from the men was subjected to WES. Briefly, gDNA was enriched using xGen Exome Research Panel V.1.0 and sequenced using the HiSeq2000 sequencing platform (Illumina). For data analysis, the Genome Analysis Toolkit package was used following the recommended best practices, including base recalibration variant calling with Haplotype Caller, variant quality score recalibration, and variant annotation using the ANNOVAR software. Suspicious variants were selected using the following criteria: (i) a low allele frequency (< 1%) in the Genome AD and Exome Aggregation Consortium databases; (ii) homozygous or compound-heterozygous variants; (iii) exonic nonsynonymous or splice-site variants or coding INDELs; and (iv) proteins that were specifically expressed in human testicular tissue. After filtering, deleterious missense variants were predicted by SIFT and MutationTaster analysis, before validating by Sanger sequencing. The primers used for the validation of variants in *IQCN* are listed in Appendix Table S3.

Semen parameter and sperm morphology analyses

For semen parameters of the affected individuals, semen samples were collected through masturbation following 2–7 days of sexual abstinence. Semen analysis was conducted according to the WHO Standards, Fifth Edition (Cooper *et al*, 2010). After liquefaction, the

© 2022 The Authors

sperm concentration, viability, and motility were analyzed by a sperm analysis system (SAS Medical).

To estimate the sperm counts of mice, sperm was released from the epididymides into 1 ml of human tubal fluid (HTF; Nanjing Aibei Biotechnology, M1130) medium for 15 min at 37°C. Sperm counts were analyzed by a computer-assisted sperm analysis system (CASA; SAS medical).

For sperm morphology analysis, 20 μL of semen was spread over slides, dried at room temperature, and fixed in 95% ethanol for hematoxylin and eosin (H&E) staining. Spermatozoa were then assessed by ×100 oil-immersion bright-field objective. Anomalies of the head, including tapered head, thin head, microcephalic, macrocephalic, multiple heads, abnormal postacrosomal region, and abnormal acrosomal region, were assessed. We examined at least 200 spermatozoa to evaluate the percentage of morphologically abnormal spermatozoa.

Transmission electron microscopy

Spermatozoa from humans and mice and testes from mice were used for TEM. Briefly, the testes and spermatozoa were fixed with 2.5% glutaraldehyde (Sigma Aldrich, G5882) overnight at 4°C. The samples were immersed in 1% osmium tetroxide, dehydrated with graded concentrations of ethanol, and embedded in Epon 812, dodecenylsuccinic anhydride, methylnadic anhydride, and dimethylaminomethyl phenol. Ultrathin, 80-nm-thick sections were stained with uranyl acetate and lead citrate, and the ultrastructure in the head of spermatozoa and elongating spermatids were observed and then photographed via a G2 Spirit TWIN transmission electron microscope (Tecnai) with a GatanCCD camera (Orius) system.

Mouse models

The *Iqcn*-knockout (*Iqcn*$^{-/-}$) and wild-type (WT) C57BL/6J mice were purchased from Cyagen Biosciences Inc. The *Iqcn*$^{-/-}$ mouse model was generated using CRISPER-Cas9 technology. Briefly, to generate *Iqcn*$^{-/-}$ mice, we designed single-guide RNAs (Appendix Table S4) against exon 3 and exon 4 of *Iqcn*. The gRNA to the mouse *Iqcn* gene and Cas9 mRNA were co-injected into fertilized mouse eggs to generate targeted knockout offspring. The founder mice were identified by PCR followed by Sanger sequencing (Appendix Table S5). The mice were maintained under specific pathogen-free (SPF) conditions at 21–23°C and had free access to water and food. This study was conducted in accordance with the recommendations of the *Guide for the Care and Use of Laboratory Animals*.

Fertility assessment of mice

Three sexually mature WT and *Iqcn*$^{-/-}$ male mice (8–10 weeks, n = 3) were mated with WT C57BL/6J female mice (4–6 weeks, n = 3) to assess fertility. The vaginal plugs of the mice were examined every morning, and the mice with vaginal plugs were separately fed. The number of pups per litter was recorded during the fertility assessment of *Iqcn*$^{-/-}$ and WT mice. If the female mice did not generate pups within 22 days, the mice were deemed not pregnant and euthanized to confirm the result. Each male mouse underwent six cycles of the breeding assay.

Acrosome reaction assay

Sperm from sexually mature WT and *Iqcn*$^{-/-}$ mice (8–10 weeks) were collected from the cauda epididymides in HTF medium for 40 min at 37°C to capacitation. Then, the induced acrosome reaction (AR) was treated with 15 μM Ca^{2+} ionophore A23187 (Sigma, C9275), and the spontaneous AR was treated in HTF medium for 30 min at 37°C. After fixation in 4% paraformaldehyde (PFA, Sangon Biotech, E672002), the percentage of sperm with AR was determined by peanut lectin (1:100; Sigma Aldrich, L7381) staining. At least 200 spermatozoa were assayed for three independent experiments to calculate the percentage of sperm with AR.

ICSI for mice

Three-week-old C57BL/6J female mice were first stimulated with 10 IU of pregnant mare serum gonadotropin (PMSG, Solarbio, P9970), and then injected with 10 IU of human chorionic gonadotropin (hCG, Livzon) 48 h later. The sperm from sexually mature WT and *Iqcn*$^{-/-}$ mice (8–10 weeks) was collected from the cauda epididymides and allowed to "swim out" in 1 mL of HTF medium. For ICSI, oocytes obtained from the oviduct of superovulated female mice and the cumulus were removed by incubation of hyaluronidase from bovine testes (Sigma, H4272). The sperm head was injected into an oocyte as previously described (Lyu *et al*, 2010). Then, the injected oocytes were cultured in KSOM medium in 6% CO_2 at 37°C. The 2PN rates, 2-cell embryo rates, and blastocyst rates were evaluated.

Ca^{2+} monitoring

Oocytes obtained from the oviduct of superovulated female mice were preincubated in 7.5 μM Ca^{2+}-sensitive fluorescent dye Fluo-3 AM (Beyotime, S1056) for 30 min at 37 °C. The oocytes were washed and injected with spermatozoa from WT and *Iqcn*$^{-/-}$ mice. Then, the injected oocytes were transferred to a dish with KSOM medium drops and monitored using the Celldiscoverer 7 fluorescence microscope (Zeiss) in 6% CO_2, 5% O_2, and 89% N_2 at 37°C. The fluorescence intensity was measured by recording the intensity of fluorescence induced by 519 nm excitation. Images were acquired every 5 s for a duration of 3 h, and intracellular Ca^{2+} oscillations were recorded based on the fluorescence intensity.

Immunostaining analyses

For immunostaining of the testes, testes were obtained from 8- to 10-week-old WT and *Iqcn*$^{-/-}$ mice and fixed in 4% PFA for 24 h at room temperature. The testes were embedded in paraffin and sectioned into slides. The slices were deparaffinized with xylene and alcohol, retrieved in sodium citrate buffer, and heated in a microwave oven for antigenic retrieval. Endogenous peroxidase activity was blocked using 0.3% hydrogen peroxidase for 10 min. A Novolink Polymer detection kit (Leica Microsystems, RE7140-K) was used for immunostaining according to the manufacturer's protocol (Fan *et al*, 2019). For immunostaining of sperm, the spermatozoa from WT and *Iqcn*$^{-/-}$ mice were fixed with 4% PFA for 30 min. The spermatozoa were then subjected to permeabilization with 1% Triton X-100 (Sigma, T8787) for 15 min and blocking

© 2022 The Authors

with 5% donkey serum albumin (Jackson ImmunoResearch, 017-000-121) at 4°C overnight. For immunostaining of unfertilized oocytes, the oocytes injected with spermatozoa from WT and *Iqcn*$^{-/-}$ mice were fixed with 4% PFA for 30 min, permeabilized with 0.5% Triton X-100 for 20 min and blocked with 5% donkey serum albumin at 4°C overnight.

The primary antibodies used in the study were as follows: rabbit polyclonal KIAA1683 (IQCN) antibody (1:200; Invitrogen, PA5-59972), mouse monoclonal calmodulin antibody (1:100; Invitrogen, MA3-917), rabbit polyclonal Phospholipase C-zeta-1 (PLC-zeta-1) antibody (1:200, Covalab, PAB0367-P [human]; 1:200, Abcam, ab124446 [mouse]) and mouse monoclonal α-tubulin-FITC antibody (1:200; Sigma Aldrich, F2168). The samples were then incubated with secondary antibodies (Invitrogen), lectin from peanut (1:100), and 4′, 6′-diamidino-2-phenylindole (DAPI, Sigma Aldrich, D8417). The Celldiscoverer 7 Fluorescence microscope (Zeiss) and Zen 2 software were used to photograph fluorescence signals. The stages of the seminiferous epithelium cycle and spermatid development were determined as previously described (Hess & Renato de Franca, 2008).

Immunoprecipitation and immunoblot analyses

IQCN IP was conducted as described previously with some modifications (Yuan *et al*, 2015). In brief, 10 μg of rabbit polyclonal IQCN antibody (synthesized by YouKe Biotechnology [immunogen: 1–200 amino acids in mouse IQCN], China) was cross-linked to 30 μl of Protein A/G-coated magnetic beads from the Pierce™ Crosslink Magnetic IP kit (Thermo Fisher, 88805) using standard methods. Two 8-week-old WT and *Iqcn*$^{-/-}$ mice testes were homogenized in 800 μl of lysis buffer and protease inhibitor PMSF (Beyotime, ST506). The lysate particles were removed by centrifugation at 20,000 *g* for 15 min at 4°C. The lysate was then cleared by incubation with 15 μl prepared beads. The lysate and beads were incubated at 4°C overnight on an orbital shaker. After elution and neutralization, the eluents were used for the immunoblot analysis.

The proteins in the eluents and input solutions were incubated at 70°C for 10 min. The protein samples were separated on 4–20% FuturePAGE™ gels (ACE, F15420Gel) in MOPS–SDS Running Buffer (ACE, F00001Gel) at 160 V for 40 min. The gels were then transferred to a polyvinylidene difluoride (PVDF) membrane (Millipore), which was incubated overnight at 4°C with the IQCN antibody (1:1,000; synthesized by YouKe Biotechnology), calmodulin antibody (1:1,000; Abcam, ab45689), and GAPDH antibody (1:1,000; Beyotime, AF0006). The membranes were then washed in TBST and incubated with HRP-conjugated secondary antibodies (1:5,000; Beyotime, A0208, and A0216) for 2 h at room temperature. Then, the blots were revealed using the SuperSignal™ West Atto Ultimate Sensitivity Substrate (Thermo Fisher, A38554). The assays were repeated three times.

Cell transfection and calmodulin antagonist treatment

We constructed plasmids encoding wild-type (WT) *IQCN* (pCDNA3.1-*IQCN*^{His}), mutant *IQCN* (M1: pCDNA3.1-*IQCN*^{His}-p.Gln304Ter and M2: pCDNA3.1-*IQCN*^{His}-p.Gln818Argfs*9), and *CALM3* (pCDNA3.1-*CALM3*^{Flag}). HeLa cells (ATCC, USA) were authenticated and tested for mycoplasma contamination. They were grown to 70–80% density in Dulbecco's Modified Eagle's Medium (DMEM; GIBCO, 11965092) with 10% fetal bovine serum (Hyclone, SH30070). Transient transfections were performed using Lipofectamine 3000 (Thermo Fisher Scientific, L3000015) in accordance with the manufacturer's protocol. The cells were divided into four groups as follows: transfected with the negative control (NC) with overexpression (oe)-*CALM3*, WT-*IQCN* with oe-*CALM3*, M1-*IQCN* with oe-*CALM3*, or M2-*IQCN* with oe-*CALM3* plasmids. The calmodulin antagonist, W-7 (Sigma Aldrich, 681629) was dissolved in DMSO. After incubation in DMEM for 24 h, HeLa cells were treated with 30 μM of W-7 and incubated for 48 h while transfecting WT + oe-*CALM3*. The medium was changed to a fresh complete medium every 24 h. After cell transfection and W7 treatment, the cells were fixed in 4% PFA and used for immunostaining analysis with mouse monoclonal calmodulin antibody (1:100; Invitrogen, MA3-917) and mouse monoclonal α-tubulin-FITC antibody (1:200; Sigma Aldrich, F2168). The percentage of multipolar spindles was calculated in HeLa cells with three replicates.

The paper explained

Problem

Fertilization is the process by which male and female gametes are fused to initiate the development of a new organism. The total fertilization failure (TFF) is a common difficulty in assisted reproductive technology. Among couples with TFF, severe sperm head deformity is a frequent reason for male-source TFF. However, the genetic basis and molecular mechanisms of TFF caused by male factors remain to be clarified.

Results

We identified two homozygous pathogenic variants of a novel gene IQ motif-containing N (*IQCN*) by whole-exome sequencing of 42 males with TFF after ICSI. Abnormal acrosome structures were found in the spermatozoa of two affected individuals. We generated *Iqcn* (*Iqcn*$^{-/-}$) knockout mice using CRISPR-Cas9 technology, which exhibited similar phenotypes as observed in humans. *In vivo* and *in vitro* studies confirmed that IQCN regulates microtubule nucleation during manchette assembly through calmodulin and calmodulin-related binding proteins. ICSI with assisted oocyte activation was successfully used to overcome genetic infertility. Our results suggest that IQCN plays an important role in spermatogenesis and male infertility by interacting with calmodulin to affect manchette assembly.

Impact

We provide direct evidence that homozygous variation of IQCN leads to abnormal acrosome structure and male infertility. These findings provide new insight into the relationship between manchette assembly and fertilization during spermatogenesis and provide a target for the early diagnosis and treatment of male TFF. We also reveal that the IQCN–calmodulin complex regulates manchette assembly to affect the formation of the sperm head, resulting in defects in oocyte activation.

Liquid chromatography-mass spectrometry (LC–MS) and bioinformatics analysis

Reciprocal IP of calmodulin was performed. First, 10 μg of calmodulin 1/2/3 antibody (Abcam, ab2860) was used for IP. The IP eluents were collected for subsequent LC–MS analysis by a Q ExactiveTM

 © 2022 The Authors

HF-X (ThermoFisher Scientific) with a nano-electrospray ion source in the Jingjie PTM BioLabs. The raw data files were searched against the Mus_musculus_10090_SP_20210721.fasta (17,089 entries) using the MaxQuant search engine (v.1.6.15.0) and its implemented Andromeda search engine to obtain protein identifications and their respective label-free quantification (LFQ) values using standard parameters. The ProteinGroup.txt file was analyzed by the following steps: (i) contaminant proteins, reverse sequences, and proteins identified "only by site" were filtered out; and (ii) the LFQ intensity was normalized by the median of commonly identified proteins in each sample and the missing values were imputed with the minimum value. Differentially expressed proteins were selected with the fold change of the normalized mean LFQ in the WT and *Iqcn*$^{-/-}$ groups, which were enriched by more than 1.5-fold. The enrichment analysis was performed using the Metascape for GO databases against the mouse reference proteome. An adjusted *P*-value cut-off of 0.05 was used to determine the significantly enriched GO term. The prediction of the protein–protein interaction network was performed using the STRING database with high confidence (0.7) and was visualized using Cytoscape (Version 3.9.1) (degree > 2). All other figures were generated in R (Version 4.0.2) using the package ggplot2 (Version 3.3.5). The immunoblot of the DEPs (IFT74 and IFT81) was conducted using IFT74 polyclonal antibody (1:1,000; Proteintech, 27334-1-AP) and IFT81 polyclonal antibody (1:1,000; Proteintech, 11744-1-AP). The validation assays were repeated three times.

ICSI with AOA in mice and humans

For AOA using spermatozoa in mice, oocytes were obtained from the oviduct of superovulated WT females. Then, sperm were collected from the cauda epididymides of *Iqcn*$^{-/-}$ mice. The oocytes were exposed to a solution of 5 μM Ca^{2+} ionophore A23187 for 5 min after injection, before washing extensively and culturing in 6% CO_2 at 37°C in a humidified incubator.

For AOA to the partner of the affected individuals, ovarian stimulation was performed and follicular maturation was triggered by human chorionic gonadotropin (HCG, Livzon), and oocyte retrieval was performed 36 h after HCG injection. Oocytes were exposed to a solution of 10 μM Ca^{2+} ionophore A23187 for 5 min after injection. The oocytes were then extensively washed and cultured in 6% CO_2, 5% O_2, and 89% N_2 at 37°C in a humidified incubator.

Statistical analysis

All data are presented as the mean ± standard error of the mean (SEM). Statistical analyses were performed using GraphPad Prism 5.01 software (GraphPad Software, Inc., USA). Data were evaluated for significance by the unpaired Student's *t*-test or one-way ANOVA, as indicated in the figure legends. Correlations were evaluated by the Spearman's correlation analysis. *P*-values < 0.05 were considered significant. All data were obtained from ≥ 3 independent experiments.

Data availability

The mass spectrometry proteomics data have been deposited to the ProteomeXchange Consortium via the PRIDE partner repository with the dataset identifier PXD036197 (http://www.ebi.ac.uk/pride/archive/projects/PXD036197). The WES data have been deposited in the Genome Variation Map in National Genomics Data Center, China National Center for Bioinformation / Beijing Institute of Genomics, Chinese Academy of Sciences, under accession number GVM000400 that can be publicly accessible at http://bigd.big.ac.cn/gvm/getProjectDetail?project = GVM000400.

Expanded View for this article is available online.

Acknowledgements

We wish to thank the patients for participating in and supporting this study. We also wish to thank the Center of Cryo-Electron Microscopy at Central South University for their technical support. This work was supported by grants from the National Natural Science Foundation of China (81974230 and 82202053), the Changsha Municipal Natural Science Foundation (kq2202072), the Hunan Provincial Natural Science Foundation (2022JJ40658), and the Scientific Research Foundation of Reproductive and Genetic Hospital of China International Trust Investment Corporation (CITIC) Xiangya (YNXM-202114).

Author contributions

Jing Dai: Conceptualization; funding acquisition; investigation; writing – original draft. **Qi Li:** Data curation; validation; investigation. **Qinwei Zhou:** Investigation. **Shen Zhang:** Investigation. **Junru Chen:** Visualization. **Yize Wang:** Validation. **Jing Guo:** Supervision. **Yifan Gu:** Resources. **Fei Gong:** Resources; supervision. **Yueqiu Tan:** Writing – review and editing. **Guangxiu Lu:** Conceptualization; writing – review and editing. **Wei Zheng:** Conceptualization; writing – review and editing. **Ge Lin:** Conceptualization; supervision; funding acquisition; writing – review and editing.

Disclosure and competing interests statement

The authors declare that they have no conflict of interest.

For more information

i GenBank, https://www.ncbi.nlm.nih.gov/genbank/
ii OMIM, https://www.omim.org/
iii ExAC Browser, https://gnomad.broadinstitute.org/
iv gnomAD, https://gnomad.broadinstitute.org/
v GTEx, http://gtexportal.org/home/index.html
vi MutationTaster, http://www.mutationtaster.org/
vii SIFT, https://sift.bii.a-star.edu.sg/
viii MultiAlin, http://multalin.toulouse.inra.fr/multalin/multalin.html
ix SMART, http://smart.embl-heidelberg.de/
x STRING, http://string-db.org/

References

Auger J, Jouannet P, Eustache F (2016) Another look at human sperm morphology. *Hum Reprod* 31: 10 – 23

Bonte D, Ferrer-Buitrago M, Dhaenens L, Popovic M, Thys V, De Croo I, De Gheselle S, Steyaert N, Boel A, van den Meerschaut F *et al* (2019) Assisted oocyte activation significantly increases fertilization and pregnancy outcome in patients with low and total failed fertilization after intracytoplasmic sperm injection: a 17-year retrospective study. *Fertil Steril* 112: 266 – 274

Chen SR, Batool A, Wang YQ, Hao XX, Chang CS, Cheng CY, Liu YX (2016) The control of male fertility by spermatid-specific factors: searching for contraceptive targets from spermatozoon's head to tail. *Cell Death Dis* 7: e2472

© 2022 The Authors

Cooper TG, Noonan E, von Eckardstein S, Auger J, Baker HW, Behre HM, Haugen TB, Kruger T, Wang C, Mbizvo MT *et al* (2010) World Health Organization reference values for human semen characteristics. *Hum Reprod Update* 16: 231 – 245

Dai J, Dai C, Guo J, Zheng W, Zhang T, Li Y, Lu C, Gong F, Lu G, Lin G (2020) Novel homozygous variations in PLCZ1 lead to poor or failed fertilization characterized by abnormal localization patterns of PLCzeta in sperm. *Clin Genet* 97: 347 – 351

Dai J, Zhang T, Guo J, Zhou Q, Gu Y, Zhang J, Hu L, Zong Y, Song J, Zhang S *et al* (2021) Homozygous pathogenic variants in ACTL9 cause fertilization failure and male infertility in humans and mice. *Am J Hum Genet* 108: 469 – 481

Fan X, Li X, Li Y, Liao J, Chen H, Li Y, Lu GX, Lin G, Gong F (2019) Endometrial CD138 count appears to be a negative prognostic indicator for patients who have experienced previous embryo transfer failure. *Fertil Steril* 112: 1103 – 1111

Fang P, Xu W, Li D, Zhao X, Dai J, Wang Z, Yan X, Qin M, Zhang Y, Xu C *et al* (2015) A novel acrosomal protein, IQCF1, involved in sperm capacitation and the acrosome reaction. *Andrology* 3: 332 – 344

Grasa P, Coward K, Young C, Parrington J (2008) The pattern of localization of the putative oocyte activation factor, phospholipase Czeta, in uncapacitated, capacitated, and ionophore-treated human spermatozoa. *Hum Reprod* 23: 2513 – 2522

Heindryckx B, Van der Elst J, De Sutter P, Dhont M (2005) Treatment option for sperm- or oocyte-related fertilization failure: assisted oocyte activation following diagnostic heterologous ICSI. *Hum Reprod* 20: 2237 – 2241

Heindryckx B, De Gheselle S, Gerris J, Dhont M, De Sutter P (2008) Efficiency of assisted oocyte activation as a solution for failed intracytoplasmic sperm injection. *Reprod Biomed Online* 17: 662 – 668

Hess RA, Renato de Franca L (2008) Spermatogenesis and cycle of the seminiferous epithelium. *Adv Exp Med Biol* 636: 1 – 15

Huang Q, Li W, Zhou Q, Awasthi P, Cazin C, Yap Y, Mladenovic-Lucas L, Hu B, Jeyasuria P, Zhang L *et al* (2021) Leucine zipper transcription factor-like 1 (LZTFL1), an intraflagellar transporter protein 27 (IFT27) associated protein, is required for normal sperm function and male fertility. *Dev Biol* 477: 164 – 176

Jang S, Lee J, Mathews J, Ruess H, Williford AO, Rangan P, Betran E, Buszczak M (2021) The *Drosophila* ribosome protein S5 paralog RpS5b promotes germ cell and follicle cell differentiation during oogenesis. *Development* 148: dev199511

Kann ML, Feinberg J, Rainteau D, Dadoune JP, Weinman S, Fouquet JP (1991) Localization of calmodulin in perinuclear structures of spermatids and spermatozoa: a comparison of six mammalian species. *Anat Rec* 230: 481 – 488

Kashir J, Heindryckx B, Jones C, De Sutter P, Parrington J, Coward K (2010) Oocyte activation, phospholipase C zeta and human infertility. *Hum Reprod Update* 16: 690 – 703

Kashir J, Ganesh D, Jones C, Coward K (2022) Oocyte activation deficiency and assisted oocyte activation: mechanisms, obstacles and prospects for clinical application. *Hum Reprod Open* 2022: hoac003

Khawar MB, Gao H, Li W (2019) Mechanism of acrosome biogenesis in mammals. *Front Cell Dev Biol* 7: 195

Kierszenbaum AL (2002) Intramanchette transport (IMT): managing the making of the spermatid head, centrosome, and tail. *Mol Reprod Dev* 63: 1 – 4

Laiho A, Kotaja N, Gyenesei A, Sironen A (2013) Transcriptome profiling of the murine testis during the first wave of spermatogenesis. *PLoS One* 8: e61558

Lehti MS, Sironen A (2016) Formation and function of the manchette and flagellum during spermatogenesis. *Reproduction* 151: R43 – R54

Li RK, Tan JL, Chen LT, Feng JS, Liang WX, Guo XJ, Liu P, Chen Z, Sha JH, Wang YF *et al* (2014) Iqcg is essential for sperm flagellum formation in mice. *PLoS One* 9: e98053

Li J, Zheng X, Lian Y, Li M, Lin S, Zhuang X, Chen L, Liu P, Qiao J (2019) Artificial oocyte activation improves cycles with prospects of ICSI fertilization failure: a sibling oocyte control study. *Reprod Biomed Online* 39: 199 – 204

Lyu QF, Deng L, Xue SG, Cao SF, Liu XY, Jin W, Wu LQ, Kuang YP (2010) New technique for mouse oocyte injection via a modified holding pipette. *Reprod Biomed Online* 21: 663 – 666

Marshall CB, Nishikawa T, Osawa M, Stathopulos PB, Ikura M (2015) Calmodulin and STIM proteins: two major calcium sensors in the cytoplasm and endoplasmic reticulum. *Biochem Biophys Res Commun* 460: 5 – 21

Martin Bahler AR (2002) Calmodulin signaling via the IQ motif. *FEBS Lett* 513: 107 – 113

Mejia-Flores I, Chiquete-Felix N, Palma-Lara I, Uribe-Carvajal S, de Lourdes Juarez-Mosqueda M (2017) During capacitation in bull spermatozoa, Actin and PLC-zeta undergo dynamic interactions. *Zygote* 25: 558 – 566

Modarres P, Tavalaee M, Ghaedi K, Nasr-Esfahani MH (2019) An overview of the Globozoospermia as a multigenic identified syndrome. *Int J Fertil Steril* 12: 273 – 277

Nasr-Esfahani MH, Deemeh MR, Tavalaee M (2010) Artificial oocyte activation and intracytoplasmic sperm injection. *Fertil Steril* 94: 520 – 526

Navarrete FA, Garcia-Vazquez FA, Alvau A, Escoffier J, Krapf D, Sanchez-Cardenas C, Salicioni AM, Darszon A, Visconti PE (2015) Biphasic role of calcium in mouse sperm capacitation signaling pathways. *J Cell Physiol* 230: 1758 – 1769

Qu W, Yuan S, Quan C, Huang Q, Zhou Q, Yap Y, Shi L, Zhang D, Guest T, Li W *et al* (2020) The essential role of intraflagellar transport protein IFT81 in male mice spermiogenesis and fertility. *Am J Physiol Cell Physiol* 318: C1092 – C1106

Schlingmann K, Michaut MA, McElwee JL, Wolff CA, Travis AJ, Turner RM (2007) Calmodulin and CaMKII in the sperm principal piece: evidence for a motility-related calcium/calmodulin pathway. *J Androl* 28: 706 – 716

Schmiady H, Tandler-Schneider A, Kentenich H (1996) Premature chromosome condensation of the sperm nucleus after intracytoplasmic sperm injection. *Hum Reprod* 11: 2239 – 2245

Shan Y, Zhao H, Zhao D, Wang J, Cui Y, Bao H (2021) Assisted oocyte activation with calcium Ionophore improves pregnancy outcomes and offspring safety in infertile patients: a systematic review and meta-analysis. *Front Physiol* 12: 751905

Shi L, Zhou T, Huang Q, Zhang S, Li W, Zhang L, Hess RA, Pazour GJ, Zhang Z (2019) Intraflagellar transport protein 74 is essential for spermatogenesis and male fertility in micedagger. *Biol Reprod* 101: 188 – 199

Swann K (2022) SPERM FACTORS AND EGG ACTIVATION: PLCzeta as the sperm factor that activates eggs: 20 years on. *Reproduction* 164: E1 – E4

Wang X, Sha YW, Wang WT, Cui YQ, Chen J, Yan W, Hou XT, Mei LB, Yu CC, Wang J (2019) Novel IFT140 variants cause spermatogenic dysfunction in humans. *Mol Genet Genomic Med* 7: e920

van der Westerlaken L, Helmerhorst F, Dieben S, Naaktgeboren N (2005) Intracytoplasmic sperm injection as a treatment for unexplained total fertilization failure or low fertilization after conventional in vitro fertilization. *Fertil Steril* 83: 612 – 617

Xin A, Qu R, Chen G, Zhang L, Chen J, Tao C, Fu J, Tang J, Ru Y, Chen Y *et al* (2020) Disruption in ACTL7A causes acrosomal ultrastructural defects in

© 2022 The Authors

human and mouse sperm as a novel male factor inducing early embryonic arrest. *Sci Adv* 6: eaaz4796

Yeste M, Jones C, Amdani SN, Patel S, Coward K (2016) Oocyte activation deficiency: a role for an oocyte contribution? *Hum Reprod Update* 22: 23–47

Yoshida T, Ioshii SO, Imanaka-Yoshida K, Izutsu K (1994) Association of cytoplasmic dynein with manchette microtubules and spermatid nuclear envelope during spermiogenesis in rats. *J Cell Sci* 107: 625–633

Yuan S, Stratton CJ, Bao J, Zheng H, Bhetwal BP, Yanagimachi R, Yan W (2015) Spata6 is required for normal assembly of the sperm connecting piece and tight head-tail conjunction. *Proc Natl Acad Sci U S A* 112: E430–E439

Zhang S, Liu Y, Huang Q, Yuan S, Liu H, Shi L, Yap YT, Li W, Zhen J, Zhang L *et al* (2020) Murine germ cell-specific disruption of Ift172 causes defects in spermiogenesis and male fertility. *Reproduction* 159: 409–421

License: This is an open access article under the terms of the Creative Commons Attribution License, which permits use, distribution and reproduction in any medium, provided the original work is properly cited.

Expanded View Figures

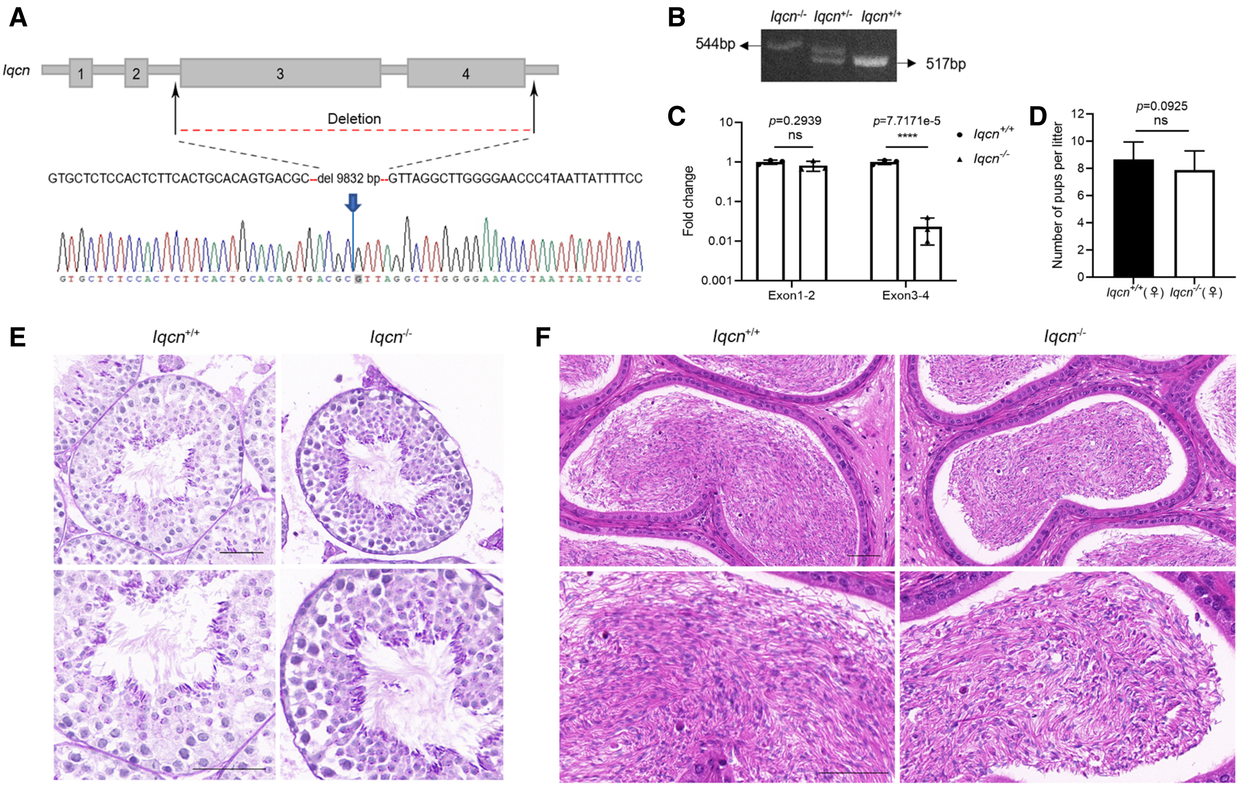

Figure EV1. Identification of *Iqcn*-knockout mice and analysis of spermatogenesis.

A Schematic diagram of the *Iqcn*-knockout strategy; confirmation by Sanger sequencing is shown below the diagram.

B Identification of *Iqcn* homozygous knockout mice by gel electrophoresis.

C Validation of the knockout efficiency in mice by qRT–PCR ($n = 3$). The fold changes of exons 1–2 and exons 3–4 are shown in the histogram.

D Fertility assessment experiments in WT and $Iqcn^{-/-}$ female mice after mating with WT male mice ($n = 18$).

E The histomorphology of seminiferous tubules by PAS staining of testes in WT and $Iqcn^{-/-}$ male mice. Scale bars, 50 μm.

F The histomorphology of epididymis in WT and $Iqcn^{-/-}$ male mice. Scale bars, 50 μm.

Data information: Data represent mean ± SEM. $^{****}P < 0.0001$, ns represents no significance by the one-way ANOVA (C) or by the unpaired Student's *t*-test (D).

 © 2022 The Authors

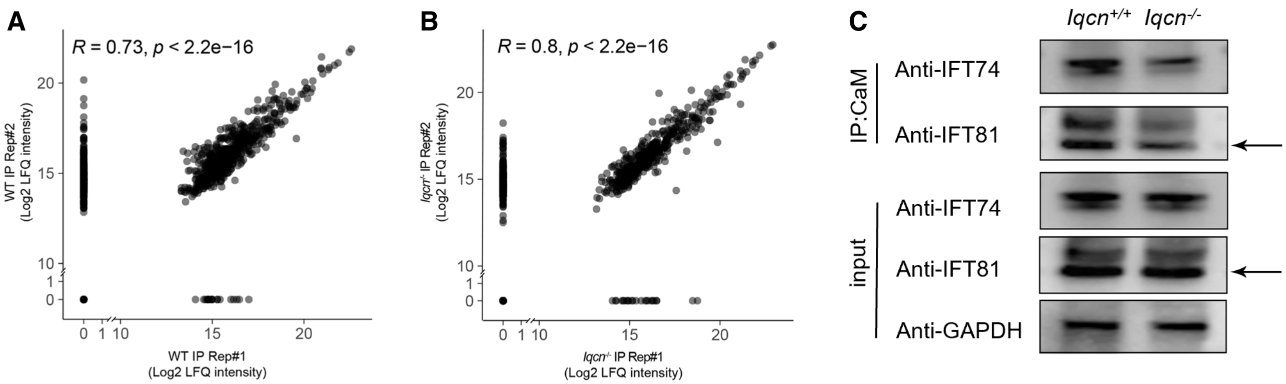

Figure EV2. Data quality control between the two groups of replicates.

- A Mass spectrometry results from two replicates in the WT group.
- B Mass spectrometry results from two replicates in the *Iqcn*$^{-/-}$ group.
- C Interaction of calmodulin with IFT74 and IFT81 following co-IP by calmodulin antibody in testes from WT and *Iqcn*$^{-/-}$ male mice ($n = 3$). The interaction of calmodulin with IFT74 and IFT81 was lower in *Iqcn*$^{-/-}$ mice than in WT mice. GAPDH was used as the internal control.

Data information: Mass spectrometry results evaluated by the Spearman's correlation analysis (A and B).
Source data are available online for this figure.

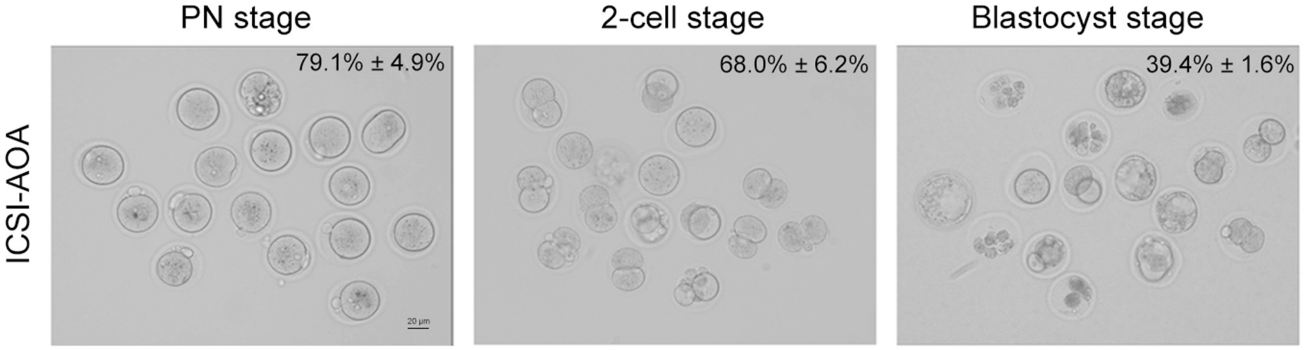

Figure EV3. Outcomes of ICSI with AOA treatment using sperm from *Iqcn*$^{-/-}$ mice.

The percentages of PN stage, 2-cell stage, and blastocyst stage were 79.1% ± 4.9%, 68.0% ± 6.2%, and 39.4% ± 1.6%, respectively ($n = 3$ biological replicates). Scale bars, 20 μm.

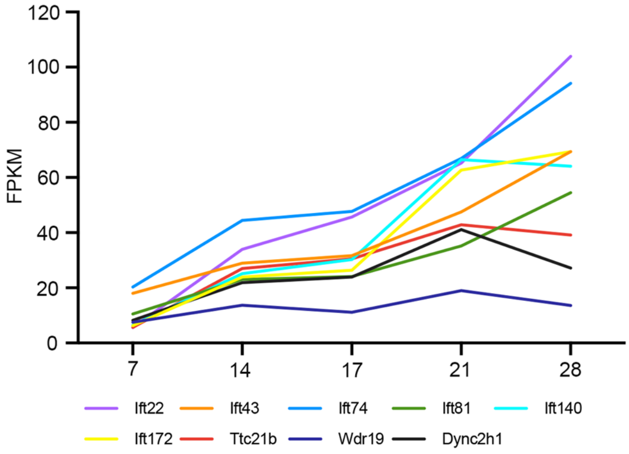

Figure EV4. Expression of IFT family genes during spermatogenesis.

According to the transcriptome sequencing data of Laiho *et al* (2013), the expression of IFT family genes increased with the growth of development days during spermatogenesis.

 © 2022 The Authors
