# Supplementary material for: IQCN disruption causes fertilization failure and male infertility due to manchette assembly defect
Source: EMBO Mol Med. 2022 Nov 2;14(12):e16501. doi: 10.15252/emmm.202216501 (PMC9728048; doi:10.15252/emmm.202216501)
Supplement: Supplementary file 10 [file EMMM-14-e16501-s003.pdf]

EMBO Molecular Medicine

IQCN disruption causes fertilization failure and male infertility due to manchette assembly defect

Jing Dai, Qi Li, Qinwei Zhou, Shen Zhang, Junru Chen, Yize Wang, Jing Guo, Yifan Gu, Fei Gong, Yue-Qiu tan, Guangxiu Lu, Wei Zheng, and Ge Lin
DOI: 10.15252/emmm.202216501

Corresponding authors: Ge Lin (linggf@hotmail.com)

Review Timeline:

Submission Date:	27th Jun 22
Editorial Decision:	21st Jul 22
Revision Received:	31st Aug 22
Editorial Decision:	22nd Sep 22
Revision Received:	29th Sep 22
Accepted:	10th Oct 22

Editor: Lise Roth

Transaction Report:

(Note: With the exception of the correction of typographical or spelling errors that could be a source of ambiguity, letters and reports are not edited. Depending on transfer agreements, referee reports obtained elsewhere may or may not be included in this compilation. Referee reports are anonymous unless the Referee chooses to sign their reports.)

21st Jul 2022

Dear Dr. Lin,

Thank you for the submission of your manuscript to EMBO Molecular Medicine. We have now heard back from the referees who agreed to evaluate your manuscript. As you will see below, the reviewers raise substantial concerns on your work, which unfortunately preclude its publication in EMBO Molecular Medicine in its current form. The reviewers find that the question addressed by the study is of potential interest, however they remain unconvinced that some of the major conclusions are sufficiently supported by the data.

If you feel you can satisfactorily address the points raised by the referees, you may wish to submit a revised version of your manuscript. Please be particularly attentive to ethical considerations and include a statement that informed consent was obtained from all subjects and that the experiments conformed to the principles set out in the WMA Declaration of Helsinki and the Department of Health and Human Services Belmont Report.

Please also attach a covering letter giving details of the way in which you have handled each of the points raised by the referees. A revised manuscript will once again be subject to review and we cannot guarantee at this stage that the eventual outcome will be favorable.
Addressing the other reviewers' concerns in full will be necessary for further considering the manuscript in our journal, and acceptance of the manuscript will entail a second round of review. EMBO Molecular Medicine encourages a single round of revision only and therefore, acceptance or rejection of the manuscript will depend on the completeness of your responses included in the next, final version of the manuscript. For this reason, and to save you from any frustrations in the end, I would strongly advise against returning an incomplete revision.

When submitting your revised manuscript, please carefully review the instructions that follow below. We perform an initial quality control of all revised manuscripts before re-review; failure to include requested items will delay the evaluation of your revision.

We require:

1) A .docx formatted version of the manuscript text (including legends for main figures, EV figures and tables). Please make sure that the changes are highlighted to be clearly visible.

2) Individual production quality figure files as .eps, .tif, .jpg (one file per figure). For guidance, download the 'Figure Guide PDF' (https://www.embopress.org/page/journal/17574684/authorguide#figureformat).

3) A .docx formatted letter INCLUDING the reviewers' reports and your detailed point-by-point responses to their comments. As part of the EMBO Press transparent editorial process, the point-by-point response is part of the Review Process File (RPF), which will be published alongside your paper.

4) A complete author checklist, which you can download from our author guidelines (https://www.embopress.org/page/journal/17574684/authorguide#submissionofrevisions). Please insert information in the checklist that is also reflected in the manuscript. The completed author checklist will also be part of the RPF.

5) It is mandatory to include a 'Data Availability' section after the Materials and Methods. Before submitting your revision, primary datasets produced in this study need to be deposited in an appropriate public database, and the accession numbers and database listed under 'Data Availability'. Please remember to provide a reviewer password if the datasets are not yet public (see https://www.embopress.org/page/journal/17574684/authorguide#dataavailability).
In case you have no data that requires deposition in a public database, please state so in this section. Note that the Data Availability Section is restricted to new primary data that are part of this study.

6) For data quantification: please specify the name of the statistical test used to generate error bars and P values, the number (n) of independent experiments (specify technical or biological replicates) underlying each data point and the test used to calculate p-values in each figure legend. The figure legends should contain a basic description of n, P and the test applied. Graphs must include a description of the bars and the error bars (s.d., s.e.m.). Please indicate the exact p values, not a range.

7) We would also encourage you to include the source data for figure panels that show essential data. Numerical data should be provided as individual .xls or .csv files (including a tab describing the data). For blots or microscopy, uncropped images should be submitted (using a zip archive if multiple images need to be supplied for one panel). Additional information on source data and instruction on how to label the files are available at
.

8) Our journal encourages inclusion of *data citations in the reference list* to directly cite datasets that were re-used and obtained from public databases. Data citations in the article text are distinct from normal bibliographical citations and should directly link to the database records from which the data can be accessed. In the main text, data citations are formatted as follows: "Data ref: Smith et al, 2001" or "Data ref: NCBI Sequence Read Archive PRJNA342805, 2017". In the Reference list, data citations must be labeled with "[DATASET]". A data reference must provide the database name, accession number/identifiers and a resolvable link to the landing page from which the data can be accessed at the end of the reference. Further instructions are available at .

9) We replaced Supplementary Information with Expanded View (EV) Figures and Tables that are collapsible/expandable online. A maximum of 5 EV Figures can be typeset. EV Figures should be cited as 'Figure EV1, Figure EV2" etc... in the text and their respective legends should be included in the main text after the legends of regular figures.
- For the figures that you do NOT wish to display as Expanded View figures, they should be bundled together with their legends in a single PDF file called *Appendix*, which should start with a short Table of Content. Appendix figures should be referred to in the main text as: "Appendix Figure S1, Appendix Figure S2" etc.
- Additional Tables/Datasets should be labeled and referred to as Table EV1, Dataset EV1, etc. Legends have to be provided in a separate tab in case of .xls files. Alternatively, the legend can be supplied as a separate text file (README) and zipped together with the Table/Dataset file.
See detailed instructions here:
.

10) For more information: There is space at the end of each article to list relevant web links for further consultation by our readers. Could you identify some relevant ones and provide such information as well? Some examples are patient associations, relevant databases, OMIM/proteins/genes links, author's websites, etc...

11) Conflict of interest: We updated our journal's competing interests policy in January 2022 and request authors to consider both actual and perceived competing interests. Please review the policy https://www.embopress.org/competing-interests and update your competing interests if necessary.

12) Every published paper now includes a 'Synopsis' to further enhance discoverability. Synopses are displayed on the journal webpage and are freely accessible to all readers. They include a short stand first (maximum of 300 characters, including space) as well as 2-5 one-sentences bullet points that summarizes the paper. Please write the bullet points to summarize the key NEW findings. They should be designed to be complementary to the abstract - i.e. not repeat the same text. We encourage inclusion of key acronyms and quantitative information (maximum of 30 words / bullet point). Please use the passive voice. Please attach these in a separate file or send them by email, we will incorporate them accordingly.
Please also suggest a striking image or visual abstract to illustrate your article as a PNG file 550 px wide x 300-600 px high.

13) As part of the EMBO Publications transparent editorial process initiative (see our Editorial at http://embomolmed.embopress.org/content/2/9/329), EMBO Molecular Medicine will publish online a Review Process File (RPF) to accompany accepted manuscripts.
In the event of acceptance, this file will be published in conjunction with your paper and will include the anonymous referee reports, your point-by-point response and all pertinent correspondence relating to the manuscript. Let us know whether you agree with the publication of the RPF and as here, if you want to remove or not any figures from it prior to publication.
Please note that the Authors checklist will be published at the end of the RPF.

EMBO Molecular Medicine has a "scooping protection" policy, whereby similar findings that are published by others during review or revision are not a criterion for rejection. Should you decide to submit a revised version, I do ask that you get in touch after three months if you have not completed it, to update us on the status.

I look forward to receiving your revised manuscript.

Yours sincerely,

Lise Roth

Lise Roth, PhD
Senior Editor
EMBO Molecular Medicine

Please use this link to login to the manuscript system and submit your revision:
Link Not Available

***** Reviewer's comments *****

Referee #1 (Remarks for Author):

Manuscript #EMM-2022-16501

Title: IQCN disruption causes fertilization failure and male infertility due to manchette assembly defect.

By: Jing Dai, Qi Li, Qinwei Zhou, Shen Zhang, Junru Chen, Jing Guo, Yifan Gu, Fei Gong, Yueqiu Tan, Guangxiu Lu, Wei Zheng, and Ge lin.

Dai et al. have reported, for the first time, that the novel IQ motif containing N (IQCN) gene, which is specifically expressed in the testis, is essential for spermatogenesis and for fertilization. In a cohort of 42 men who underwent total fertilization failure (TFF), they identified homozygous pathogenic variants of IQCN in two of them. The authors confirmed the role of IQCN in fertilization in Iqcn knockout (Iqcn-/-) mice, that showed abnormal acrosome structure and manchette assembly, probably due to lack of IQCN interaction with calmodulin. ICSI with assisted oocyte activation treatment is an efficacious treatment for both Iqcn-/- mice and men carrying homozygous IQCN variants. Therefore, this study indicates a new genetic diagnostic marker of TFF and male-derived infertility and suggests a positive treatment for men who carry homozygous IQCN variants.

The MS is well written, and the experimental design is appropriate. The topic is of interest since TFF is a frequent problem, especially in the most industrialized countries. Therefore, the research of new strategies to improve TFF can be of great help to enhance the fertilization rate.

The only issue that needs to be verified, in my opinion, independently of the fertilization, is if the mutant spermatozoa can make an appropriately acrosome reaction, to be sure that the abnormal spermatozoa can also give rise to an appropriate acrosome reaction.

Another curiosity is about the choice of two distinct primary IQCN antibodies for immunostaining (by Invitrogen) and IP (by Youke Biotechnology).

However, the paper is attractive, and the reviewer considers it worthy of publication in EMBO.

Referee #2 (Remarks for Author):

Total fertilization failure (TFF) is frequently encountered in assisted reproductive technology. However, the genetic markers of TFF were still not fully elucidated. Dai., et al. have identified a novel gene, IQCN, is indispensable for normal fertilization both in human and mice. They clarified mutant IQCN disrupted the manchette assembly and resulted in acrosome formation defects and TFF. Additionally, they found ICSI with assisted oocyte activation could overcame IQCN-associated male infertility. This work is interesting with high novelty. However, some aspects of current manuscript could be improved as mentioned below.
1. More detailed information of inclusion and exclusion criteria of the cohort is necessary. For example, globozoospermia was one of the main causes of TFF, so what is the proportion in the cohort herein.
2. The authors found IQCN biallelic variants in two individuals of local TFF cohort (n=42), what are the specific characteristics of these individuals? And what is the frequency of IQCN variants in controls?
3. The authors analyzed some potentially affected downstream pathways and differentially expressed proteins through LC-MS. It is recommended to verify the credibility of the MS data in a variety of ways.
4. How about the genotype of the sister (II-1) of the affected individual in family 1? Does IQCN defects interference the female fertility? The pedigree needs to be optimized, such as the alignment of family member numbers and genotypes.
5. The statistical analysis methods should be detailly described in the figure legends, and the p-values and sample number in each bar chart should be added accordingly.

Referee #3 (Remarks for Author):

The manuscript from Dai et al identified IQCN mutations in two individuals with from a cohort of men experiencing total fertilization failure (fertilization failure after ICSI) using whole exon sequencing. Defects in the sperm from these men revealed defects in oocyte activation after fertilization (due to PLCzeta absence from the sperm head) and defects in acrosome morphology. The authors went on to analyze mouse Iqcn by generating a similar mutation to the human patients (deletion of several but not all IQ motifs) and found that similar defects were seen in the mouse sperm. This suggest that indeed the defects

seen in the human patients are the result of mutations in IQCN. The authors then show in mouse that IQCN colocalizes with calmodulin to the manchette (a microtubule-based structure that helps shape sperm) during spermiogenesis and that in the absence of IQCN there are defects in calmodulin localization and manchette defects. Using immunoprecipitation, the authors show an association of IQCN and Calmodulin. In cell culture, the IQCN mutations (identical to the mutations found in the patients) can generate multi-polar spindles during mitosis. Multipolar spindles can also be generated with a Calmodulin antagonist, the authors suggest that these cell culture experiments show that IQCN mutations have a negative effect on Calmodulin. Immunoprecipitation of Calmodulin in the absence of IQCN from mouse testis shows a decrease in association of proteins involved in the cytoskeleton (including intraflagellar transport proteins), which demonstrates that IQCN can affect Calmodulin's proteome and that this effect is what is causing the defects in sperm morphology in the absence of Iqcn. The authors end with ICSI experiments using a calcium ionophore (A23187) to activate the fertilized oocytes. These ICSI experiments overcome the fertility defect seen in the mice. The authors then go on to perform similar ICSI experiments on human patient number 2. Using this technique, ICSI resulted in two children being born from patient number 2's sperm and his female partner's eggs.

Major points
1. There is a lack of mechanistic explanation of the failure to activate oocytes after ICSI. The authors demonstrate that PLCzeta is aberrantly localized in the absence IQCN; however, no mechanism is presented on how this happens.

2. There is a lack of novelty to the paper since another IQ motif protein (IQCG) was also shown to localize to the manchette and also shown to be involved in manchette formation.

3. We have a major concern with the ethical considerations of the ICSI experiments done with the human patients. The ask that the editors check the ethics statement and make sure all rules and regulations were followed in these ICSI experiments.

Response to Reviewers and Editorial Board

We greatly appreciate the insightful suggestions from the reviewers and editors, which have helped us to improve the quality of the manuscript. The manuscript has now been revised in accordance with these comments and suggestions, and the modified sentences or paragraphs are indicated by red text in the revised manuscript. We sincerely hope that the revision will be satisfactory for publication. Thank you again for your extensive efforts in reviewing the manuscript.

***** Reviewer’s comments *****

Referee #1 (Remarks for Author):

Dai et al. have reported, for the first time, that the novel IQ motif containing N (IQCN) gene, which is specifically expressed in the testis, is essential for spermatogenesis and for fertilization. In a cohort of 42 men who underwent total fertilization failure (TFF), they identified homozygous pathogenic variants of IQCN in two of them. The authors confirmed the role of IQCN in fertilization in Iqcn knockout (Iqcn-/-) mice, that showed abnormal acrosome structure and manchette assembly, probably due to lack of IQCN interaction with calmodulin. ICSI with assisted oocyte activation treatment is an efficacious treatment for both Iqcn-/- mice and men carrying homozygous IQCN variants. Therefore, this study indicates a new genetic diagnostic marker of TFF and male-derived infertility and suggests a positive treatment for men who carry homozygous IQCN variants.

The MS is well written, and the experimental design is appropriate. The topic is of interest since TFF is a frequent problem, especially in the most industrialized countries. Therefore, the research of new strategies to improve TFF can be of great help to enhance the fertilization rate.

Re: Thank you for your affirmation of our research. We are grateful for your

comments.

The only issue that needs to be verified, in my opinion, independently of the fertilization, is if the mutant spermatozoa can make an appropriately acrosome reaction, to be sure that the abnormal spermatozoa can also give rise to an appropriate acrosome reaction.

Re: Thank you for your suggestion. We have performed spontaneous and induced acrosome reaction (AR) assays in spermatozoa from WT and *Iqcn*$^{-/-}$ male mice as previously described (Liu et al., 2022).

We found the percentage of spontaneous AR was not significantly different between the WT and *Iqcn*$^{-/-}$ sperm (43.3% ± 1.9% vs. 43.7% ± 4.7%). After inducing the acrosome reaction with the Ca^{2+} ionophore A23187, the percentage of induced AR was neither significantly different between the WT and *Iqcn*$^{-/-}$ sperm (67.5% ± 3.0% vs. 62.9% ± 1.2%). The results indicate that the abnormal spermatozoa in *Iqcn*$^{-/-}$ mice can give rise to an appropriate acrosome reaction. The results have been added in **Figure 3J** and a related description has been added in the revised manuscript, as follows: "In the acrosome reaction (AR) assay, the percentages of sperm with spontaneous and induced AR were not significant different between spermatozoa from WT and *Iqcn*$^{-/-}$ male mice (Fig. 3J)." (Results section, page 6, lines 23–26)

The related methods are listed in the manuscript as follows: "Sperm from sexually mature WT and *Iqcn*$^{-/-}$ mice (8–10 weeks) were collected from the cauda epididymides in HTF medium for 40 min at 37°C to capacitation. Then, the induced acrosome reaction (AR) was treated by 15 μM Ca^{2+} ionophore A23187 (Sigma, C9275) and the spontaneous AR was treated in HTF medium for 30 min at 37°C. After fixation in 4% paraformaldehyde (PFA, Sangon Biotech, E672002), the percentage of sperm with AR was determined by peanut lectin (1:100; Sigma Aldrich, L7381) staining. At least 200 spermatozoa were assayed for three independent experiments to calculate the percentage of sperm with AR." (Methods and Materials section, page 16, lines 15–22)

Another curiosity is about the choice of two distinct primary IQCN antibodies for immunostaining (by Invitrogen) and IP (by Youke Biotechnology).

Re: Thank you. We purchased the antibody (PA5-59972) from Invitrogen. The antibody PA5-59972 (immunogen: 194–311 amino acids in human IQCN), as outlined on the website, is suitable for immunohistochemistry and immunofluorescence in human and mouse samples. However, this antibody was not suitable for immunoblot. For this reason, we synthesized an antibody (immunogen: 1–200 amino acids amino acids in mouse IQCN) at Youke Biotechnology. Therefore, we used two distinct primary IQCN antibodies for immunofluorescence and immunoprecipitation, respectively.

However, the paper is attractive, and the reviewer considers it worthy of publication in EMBO.

Re: Thank you for your appreciation of this study.

Referee #2 (Remarks for Author):

Total fertilization failure (TFF) is frequently encountered in assisted reproductive technology. However, the genetic markers of TFF were still not fully elucidated. Dai., et al. have identified a novel gene, IQCN, is indispensable for normal fertilization both in human and mice. They clarified mutant IQCN disrupted the manchette assembly and resulted in acrosome formation defects and TFF. Additionally, they found ICSI with assisted oocyte activation could overcame IQCN-associated male infertility. This work is interesting with high novelty. However, some aspects of current manuscript could be improved as mentioned below.

Re: Thank you for your advice. We have addressed all of your concerns

point-by-point in the revised manuscript.

1. More detailed information of inclusion and exclusion criteria of the cohort is necessary. For example, globozoospermia was one of the main causes of TFF, so what is the proportion in the cohort herein.

Re: Thanks for your professional advice. The inclusion and exclusion criteria are listed in the revised manuscript, as follows: "The inclusion criteria were as follows: (1) men with primary infertility; (2) ≥ one ICSI attempt; (3) ≥ five MII oocytes retrieved in single attempt; (4) TFF or poor fertilization (fertilization rate < 20%); (5) male-source based on the mouse oocyte activation test as previously described (Heindryckx et al., 2008, Heindryckx et al., 2005). The exclusion criteria were men with globozoospermia." (Methods and Materials section, page 14, lines 7–12)

Indeed, globozoospermia was one of the known causes of TFF due to absence of oocyte activating factor PLCζ, we excluded such patients in our cohort. We apologize for the absence of exclusion criteria. We have added the related information in the revised manuscript. (Methods and Materials section, page 14, lines 11–12)

2. The authors found IQCN biallelic variants in two individuals of local TFF cohort (n=42), what are the specific characteristics of these individuals? And what is the frequency of IQCN variants in controls?

Re: Thank you for highlighting this. The inclusion criteria of our cohort are listed in the revised manuscript. The specific characteristics of these two individuals, including medical history and basic examination, have been added in **Appendix Table S1**.

For the frequency of *IQCN* variants, both of *IQCN* variants were absent in the control populations. The related information has been listed as "Both *IQCN* variants were absent in our control database of 100 men with normal fertility." (Results section, page 4, lines 16–17)

3. The authors analyzed some potentially affected downstream pathways and differentially expressed proteins through LC-MS. It is recommended to verify the credibility of the MS data in a variety of ways.

Re: Thank you. We selected two DEPs, IFT74 and IFT81, in the IFT protein family to verify the credibility of the MS data by immunoblot. The related information has been listed in the Methods and Results section, as follows: "The immunoblot of the DEPs (IFT74 and IFT81) was conducted using IFT74 polyclonal antibody (1:1000; Proteintech, 27334-1-AP) and IFT81 polyclonal antibody (1:1000; Proteintech, 11744-1-AP). The validation assays were repeated three times." (Methods and Materials section, page 20, lines 10–13). Additionally, we added the results described as "We selected two IFT family proteins (IFT74 and IFT81) for further immunoblot verification and found the trend of change is completely consistent with that of LC-MS data (**Fig. EV2C**)." (Results section, page 10, lines 3–5)

4. How about the genotype of the sister (II-1) of the affected individual in family 1? Does IQCN defects interference the female fertility? The pedigree needs to be optimized, such as the alignment of family member numbers and genotypes.

Re: Thank you for your suggestion. Unfortunately, the genotype of member II-1 in family 1 is still unknown due to the unavailability of her DNA samples. However, due to our fertility test in homozygous female mice, we suggest IQCN is dispensable for female fertility. We have added related contents in the Results section, as follows: "For fertility assessment, *Iqcn*$^{-/-}$ male mice failed to produce offspring (Fig. 3A), while *Iqcn*$^{-/-}$ female mice have normal fertility (**Fig. EV1D**), suggesting that IQCN is exclusively indispensable for male fertility in mice." (Results section, page 6, lines 7–9)

Thanks for your kind reminder, we have optimized the pedigree in the revised manuscript.

5. The statistical analysis methods should be detailly described in the figure legends, and the p-values and sample number in each bar chart should be added accordingly.

Re: Thank you. The data were evaluated for significance by unpaired Student's t-test or one-way ANOVA, as indicated in the figure legends, and the *P*-values and sample number in each bar chart have been added according to the journal's requirement.

Referee #3 (Remarks for Author):

The manuscript from Dai et al identified IQCN mutations in two individuals with from a cohort of men experiencing total fertilization failure (fertilization failure after ICSI) using whole exon sequencing. Defects in the sperm from these men revealed defects in oocyte activation after fertilization (due to PLCzeta absence from the sperm head) and defects in acrosome morphology. The authors went on to analyze mouse Iqcn by generating a similar mutation to the human patients (deletion of several but not all IQ motifs) and found that similar defects were seen in the mouse sperm. This suggest that indeed the defects seen in the human patients are the result of mutations in IQCN. The authors then show in mouse that IQCN colocalizes with calmodulin to the manchette (a microtubule-based structure that helps shape sperm) during spermiogenesis and that in the absence of IQCN there are defects in calmodulin localization and manchette defects. Using immunoprecipitation, the authors show an association of IQCN and Calmodulin. In cell culture, the IQCN mutations (identical to the mutations found in the patients) can generate multi-polar spindles during mitosis. Multipolar spindles can also be generated with a Calmodulin antagonist, the authors suggest that these cell culture experiments show that IQCN mutations have a negative effect on Calmodulin. Immunoprecipitation of Calmodulin in the absence of IQCN from mouse testis shows a decrease in association of proteins involved in the cytoskeleton (including intraflagellar transport proteins), which demonstrates that IQCN can affect Calmodulin's proteome and that this effect is what is causing the defects in sperm morphology in the absence of Iqcn. The authors end with ICSI

experiments using a calcium ionophore (A23187) to activate the fertilized oocytes. These ICSI experiments overcome the fertility defect seen in the mice. The authors then go on to perform similar ICSI experiments on human patient number 2. Using this technique, ICSI resulted in two children being born from patient number 2's sperm and his female partner's eggs.

Re: Thank you very much for the comments, which have been helpful to improve the quality of the article. We have revised the manuscript paying close attention to your comments.

Major points

1. There is a lack of mechanistic explanation of the failure to activate oocytes after ICSI. The authors demonstrate that PLCzeta is aberrantly localized in the absence IQCN; however, no mechanism is presented on how this happens.

Re: Thank you for your suggestion. We confirmed by complementary experiments that IQCN deletion caused fertilization failure after ICSI due to the absence of PLCζ in the oocyte (**Figure 4D**) (Results section, page 7, lines 3–10).

It is well known that PLCζ is a main sperm borne oocyte activation factor. The deficiencies, abnormal localization, and reduced activity/expression of PLCζ are all associated with oocyte activation failure and TFF after ICSI (Aghajanpour et al., 2011, Heytens et al., 2009, Heytens et al., 2010, Kashir et al., 2012a, Kashir et al., 2012b, Lee et al., 2014, Nomikos et al., 2011, Yelumalai et al., 2015, Yoon et al., 2008).

A deeper question is the mechanism of PLCζ aberrantly localization. The dynamic changes of PLCζ during capacitation and acrosome reaction have been reported (Grasa et al., 2008, Mejia-Flores et al., 2017). However, the specific mechanism has not been elucidated. Previous studies by our and Sun's groups on actin-like family members (*ACTL9* and *ACTL7A*) indicated that the precise localization of PLCζ relies on normal acrosome structure (Dai et al., 2021, Xin et al., 2020). To answer your question herein, we further observed the dynamic changes of PLCζ during acrosomal

biogenesis of the Golgi phase, cap phase, acrosome phase, and maturation phase in the presence and absence of IQCN (**Figure 4F**). We found no difference in the Golgi phase (steps 1–3). However, starting from the cap phase (steps 4–7), the localization of PLCζ was markedly abnormal in *Iqcn*$^{-/-}$ mice, with a limited localization at the tip of the head, resulting in the loss of PLCζ in epididymal spermatozoa. The loss pattern of PLCζ was similar to that of *Actl7a* mutant mice (Dai et al., 2022). We speculated that PLCζ is discharged with excess cytoplasmic droplets. Above all, we established a relationship between the dynamic changes of PLCζ and acrosomal biogenesis. However, the exact molecular mechanism is under investigation. We have added related contents in the result (Results section, page 7, lines 13–25) and discussion sections (Discussion section, page 13, lines 6–13).

2. There is a lack of novelty to the paper since another IQ motif protein (IQCG) was also shown to localize to the manchette and also shown to be involved in manchette formation.

Re: We apologize for the unclear description of this part. Our research highlights a novel molecular diagnostic marker for male-source total fertilization failure derived from clinical cases and suggests a positive intervention. Currently, the role of IQ motif-containing genes family in spermiogenesis and fertilization has not been clearly elucidated. Although IQCG has a similar localization with IQCN, the phenotype is totally different. *Iqcg*$^{-/-}$ mice represent as disorganized sperm flagellum and total immobility spermatozoa. However, patients carried *IQCN* variants have not shown the asthenozoospermia phenotype (Table 3), suggesting that IQCN and IQCG are not functionally redundancy, providing a new insight into the functional differences of IQ motif-containing genes family in spermiogenesis.

3. We have a major concern with the ethical considerations of the ICSI experiments done with the human patients. The ask that the editors check the ethics statement and make sure all rules and regulations were followed in these ICSI experiments.

Re: Thanks for your professional comment. Informed consent has been obtained from all subjects and that the experiments conformed to the principles set out in the WMA Declaration of Helsinki and the Department of Health and Human Services Belmont Report. (Materials and Methods section, page 13, line 29; page 14, lines 1–6) And we have uploaded the relevant informed consent forms in the revised manuscripts.

Actually, ICSI with assisted oocyte activation (AOA) with Ca^{2+} ionophore A23187 is a technique that has been clinically applied for TFF or low fertilization rate under ICSI in assisted reproductive technology, and a series of international studies have demonstrated its effectiveness and safety (Bonte et al., 2019, Kashir et al., 2022, Li et al., 2019, Shan et al., 2021, Yeste et al., 2016). We have added related statements in the revised manuscript (Results section, page 10, lines 9–13).

The editorial office
Comments to Authors:

1) A. docx formatted version of the manuscript text (including legends for main figures, EV figures and tables). Please make sure that the changes are highlighted to be clearly visible.

Re: The changes in the revised manuscript are highlighted by revision marks.

2) Individual production quality figure files as. Eps,. Tif,. Jpg (one file per figure). For guidance, download the 'Figure Guide PDF' (https://www.embopress.org/page/journal/17574684/authorguide#figureformat).

Re: The quality of the figures has been improved according to the "Figure guidance".

3) A. docx formatted letter INCLUDING the reviewers' reports and your detailed point-by-point responses to their comments. As part of the EMBO Press transparent editorial process, the point-by-point response is part of the Review Process File (RPF),

which will be published alongside your paper.

Re: The response to reviewers has been provided with point-by-point responses.

4) A complete author checklist, which you can download from our author guidelines (https://www.embopress.org/page/journal/17574684/authorguide#submissionofrevisions). Please insert information in the checklist that is also reflected in the manuscript. The completed author checklist will also be part of the RPF.

Re: A complete author checklist has been uploaded.

5) It is mandatory to include a 'Data Availability' section after the Materials and Methods. Before submitting your revision, primary datasets produced in this study need to be deposited in an appropriate public database, and the accession numbers and database listed under 'Data Availability'. Please remember to provide a reviewer password if the datasets are not yet public (see https://www.embopress.org/page/journal/17574684/authorguide#dataavailability).
In case you have no data that requires deposition in a public database, please state so in this section. Note that the Data Availability Section is restricted to new primary data that are part of this study.

Re: The mass spectrometry proteomics data have been deposited to the ProteomeXchange Consortium via the PRIDE partner repository with the dataset identifier PXD036197. Reviewer account details: Username: reviewer_pxd036197@ebi.ac.uk; Password: KyyjnuGb.

6) For data quantification: please specify the name of the statistical test used to generate error bars and P values, the number (n) of independent experiments (specify technical or biological replicates) underlying each data point and the test used to calculate p-values in each figure legend. The figure legends should contain a basic

description of n, P and the test applied. Graphs must include a description of the bars and the error bars (s.d., s.e.m.). Please indicate the exact p values, not a range.

Re: The data have been quantified in the revised manuscript.

7) We would also encourage you to include the source data for figure panels that show essential data. Numerical data should be provided as individual. Xls or. Csv files (including a tab describing the data). For blots or microscopy, uncropped images should be submitted (using a zip archive if multiple images need to be supplied for one panel). Additional information on source data and instruction on how to label the files are available at <https://www.embopress.org/page/journal/17574684/authorguide#sourcedata>.

Re: The source data for the figure panels have been uploaded according to the guidance.

8) Our journal encourages inclusion of *data citations in the reference list* to directly cite datasets that were re-used and obtained from public databases. Data citations in the article text are distinct from normal bibliographical citations and should directly link to the database records from which the data can be accessed. In the main text, data citations are formatted as follows: "Data ref: Smith et al, 2001" or "Data ref: NCBI Sequence Read Archive PRJNA342805, 2017". In the Reference list, data citations must be labeled with "[DATASET]". A data reference must provide the database name, accession number/identifiers and a resolvable link to the landing page from which the data can be accessed at the end of the reference. Further instructions are available at <https://www.embopress.org/page/journal/17574684/authorguide#referencesformat>.

Re: Thank you. We did not re-use the datasets from public databases.

9) We replaced Supplementary Information with Expanded View (EV) Figures and Tables that are collapsible/expandable online. A maximum of 5 EV Figures can be typeset. EV Figures should be cited as 'Figure EV1, Figure EV2" etc... in the text and their respective legends should be included in the main text after the legends of regular figures.

- For the figures that you do NOT wish to display as Expanded View figures, they should be bundled together with their legends in a single PDF file called *Appendix*, which should start with a short Table of Content. Appendix figures should be referred to in the main text as: "Appendix Figure S1, Appendix Figure S2" etc.
- Additional Tables/Datasets should be labeled and referred to as Table EV1, Dataset EV1, etc. Legends have to be provided in a separate tab in case of. xls files. Alternatively, the legend can be supplied as a separate text file (README) and zipped together with the Table/Dataset file.

See detailed instructions here:
<https://www.embopress.org/page/journal/17574684/authorguide#expandedview>.

Re: Thank you. We have listed the EV Figures and Tables in the revised manuscript according to the instructions.

10) For more information: There is space at the end of each article to list relevant web links for further consultation by our readers. Could you identify some relevant ones and provide such information as well? Some examples are patient associations, relevant databases, OMIM/proteins/genes links, author's websites, etc...

Re: The relevant websites have been listed in the revised manuscript.

11) Conflict of interest: We updated our journal's competing interests policy in January 2022 and request authors to consider both actual and perceived competing interests. Please review the policy https://www.embopress.org/competing-interests and update your competing interests if necessary.

Re: The conflict of interest has been re-considered by all of the authors.

12) Every published paper now includes a ‘Synopsis’ to further enhance discoverability. Synopses are displayed on the journal webpage and are freely accessible to all readers. They include a short stand first (maximum of 300 characters, including space) as well as 2-5 one-sentences bullet points that summarizes the paper. Please write the bullet points to summarize the key NEW findings. They should be designed to be complementary to the abstract - i.e. not repeat the same text. We encourage inclusion of key acronyms and quantitative information (maximum of 30 words / bullet point). Please use the passive voice. Please attach these in a separate file or send them by email, we will incorporate them accordingly.

Please also suggest a striking image or visual abstract to illustrate your article as a PNG file 550 px wide x 300-600 px high.

Re: As suggested, we have added a Synopsis and Graphical Abstract, as shown below.

1) Synopsis:

The present study identified homozygous pathogenic variants in *IQCN* in two male patients experienced TFF. IQCN is essential for regulating nucleation during microtubule assembly by interacting with calmodulin and calmodulin-related binding proteins.

- The two affected individuals showed a significant increase in the proportion of sperm head malformations, and the acrosome structure could not be tightly anchored to the nucleus, showing the characteristics of the ruffled acrosome.
- Abnormal acrosome caused aberrant oocyte activation factor PLCζ localization and TFF after ICSI.
- The phenotype of *Iqcn* knockout (*Iqcn*$^{-/-}$) male mice is similar to that of humans.
- IQCN interacts with calmodulin to promote prolonged manchette assembly in spermatids, and the disappearance of the interaction with calmodulin after IQCN leads to nucleation defects.

- *IQCN*-related male infertility can be rescued by ICSI with assisted oocyte activation.

2) Graphical Abstract

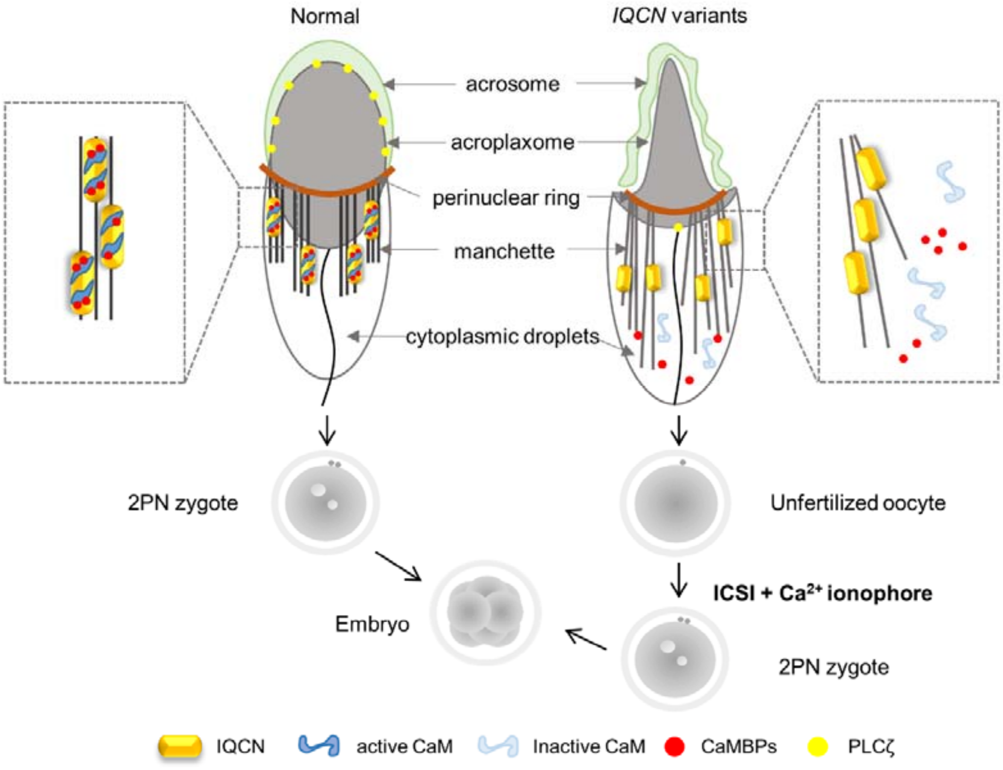

13) As part of the EMBO Publications transparent editorial process initiative (see our Editorial at http://embomolmed.embopress.org/content/2/9/329), EMBO Molecular Medicine will publish online a Review Process File (RPF) to accompany accepted manuscripts.

In the event of acceptance, this file will be published in conjunction with your paper and will include the anonymous referee reports, your point-by-point response and all pertinent correspondence relating to the manuscript. Let us know whether you agree with the publication of the RPF and as here, if you want to remove or not any figures from it prior to publication.

Please note that the Authors checklist will be published at the end of the RPF.

Re: We agree to publish the related RPF.

Reference:

Aghajanpour S, Ghaedi K, Salamian A, Deemeh MR, Tavalaee M, Moshtaghian J, Parrington J, Nasr-Esfahani MH (2011) Quantitative expression of phospholipase C zeta, as an index to assess fertilization potential of a semen sample. *Hum Reprod* 26: 2950-6

Bonte D, Ferrer-Buitrago M, Dhaenens L, Popovic M, Thys V, De Croo I, De Gheselle S, Steyaert N, Boel A, Vanden Meerschaut F *et al.* (2019) Assisted oocyte activation significantly increases fertilization and pregnancy outcome in patients with low and total failed fertilization after intracytoplasmic sperm injection: a 17-year retrospective study. *Fertil Steril* 112: 266-274

Dai J, Chen Y, Li Q, Zhang T, Zhou Q, Gong F, Lu G, Zheng W, Lin G (2022) Pathogenic variant in ACTL7A causes severe teratozoospermia characterized by bubble-shaped acrosomes and male infertility. *Mol Hum Reprod* 28

Dai J, Zhang T, Guo J, Zhou Q, Gu Y, Zhang J, Hu L, Zong Y, Song J, Zhang S *et al.* (2021) Homozygous pathogenic variants in ACTL9 cause fertilization failure and male infertility in humans and mice. *Am J Hum Genet* 108: 469-481

Grasa P, Coward K, Young C, Parrington J (2008) The pattern of localization of the putative oocyte activation factor, phospholipase Czeta, in uncapacitated, capacitated, and ionophore-treated human spermatozoa. *Hum Reprod* 23: 2513-22

Heindryckx B, De Gheselle S, Gerris J, Dhont M, De Sutter P (2008) Efficiency of assisted oocyte activation as a solution for failed intracytoplasmic sperm injection. *Reprod Biomed Online* 17: 662-8

Heindryckx B, Van der Elst J, De Sutter P, Dhont M (2005) Treatment option for sperm- or oocyte-related fertilization failure: assisted oocyte activation following diagnostic heterologous ICSI. *Hum Reprod* 20: 2237-41

Heytens E, Parrington J, Coward K, Young C, Lambrecht S, Yoon SY, Fissore RA, Hamer R, Deane CM, Ruas M *et al.* (2009) Reduced amounts and abnormal forms of phospholipase C zeta (PLCzeta) in spermatozoa from infertile men. *Hum Reprod* 24: 2417-28

Heytens E, Schmitt-John T, Moser JM, Jensen NM, Soleimani R, Young C, Coward K, Parrington J, De Sutter P (2010) Reduced fertilization after ICSI and abnormal phospholipase C zeta presence in spermatozoa from the wobbler mouse. *Reprod Biomed Online* 21: 742-9

Kashir J, Ganesh D, Jones C, Coward K (2022) Oocyte activation deficiency and assisted oocyte activation: mechanisms, obstacles and prospects for clinical application. *Hum Reprod Open* 2022: hoac003

Kashir J, Konstantinidis M, Jones C, Heindryckx B, De Sutter P, Parrington J, Wells D, Coward K (2012a) Characterization of two heterozygous mutations of the oocyte activation factor phospholipase C zeta (PLCzeta) from an infertile man by use of minisequencing of individual sperm and expression in somatic cells. *Fertil Steril* 98: 423-31

Kashir J, Konstantinidis M, Jones C, Lemmon B, Lee HC, Hamer R, Heindryckx B, Deane CM, De Sutter P, Fissore RA *et al.* (2012b) A maternally inherited autosomal point mutation in human phospholipase C zeta (PLCzeta) leads to male infertility. *Hum Reprod* 27: 222-31

Lee HC, Arny M, Grow D, Dumesic D, Fissore RA, Jellerette-Nolan T (2014) Protein phospholipase C Zeta1 expression in patients with failed ICSI but with normal sperm parameters. *J Assist Reprod Genet* 31: 749-56

Li J, Zheng X, Lian Y, Li M, Lin S, Zhuang X, Chen L, Liu P, Qiao J (2019) Artificial oocyte activation improves cycles with prospects of ICSI fertilization failure: a sibling oocyte control study. *Reprod Biomed Online* 39: 199-204

Liu R, Yan Z, Fan Y, Qu R, Chen B, Li B, Wu L, Wu H, Mu J, Zhao L *et al.* (2022) Bi-allelic variants in KCNU1 cause impaired acrosome reactions and male infertility. *Hum Reprod* 37: 1394-1405

Mejia-Flores I, Chiquete-Felix N, Palma-Lara I, Uribe-Carvajal S, de Lourdes Juarez-Mosqueda M (2017) During capacitation in bull spermatozoa, actin and PLC-zeta undergo dynamic interactions. *Zygote* 25: 558-566

Nomikos M, Elgmati K, Theodoridou M, Calver BL, Cumbes B, Nounesis G, Swann K, Lai FA (2011) Male infertility-linked point mutation disrupts the Ca2+ oscillation-inducing and PIP(2) hydrolysis activity of sperm PLCzeta. *Biochem J* 434: 211-7

Shan Y, Zhao H, Zhao D, Wang J, Cui Y, Bao H (2021) Assisted Oocyte Activation With Calcium Ionophore Improves Pregnancy Outcomes and Offspring Safety in Infertile Patients: A Systematic Review and Meta-Analysis. *Front Physiol* 12: 751905

Xin A, Qu R, Chen G, Zhang L, Chen J, Tao C, Fu J, Tang J, Ru Y, Chen Y *et al.* (2020) Disruption in ACTL7A causes acrosomal ultrastructural defects in human and mouse sperm as a novel male factor inducing early embryonic arrest. *Sci Adv* 6: eaaz4796

Yelumalai S, Yeste M, Jones C, Amdani SN, Kashir J, Mounce G, Da Silva SJ, Barratt CL, McVeigh E, Coward K (2015) Total levels, localization patterns, and proportions of sperm exhibiting phospholipase C zeta are significantly correlated with fertilization rates after intracytoplasmic sperm injection. *Fertil Steril* 104: 561-8 e4

Yeste M, Jones C, Amdani SN, Patel S, Coward K (2016) Oocyte activation deficiency: a role for an oocyte contribution? *Hum Reprod Update* 22: 23-47

Yoon SY, Jellerette T, Salicioni AM, Lee HC, Yoo MS, Coward K, Parrington J, Grow D, Cibelli JB, Visconti PE *et al.* (2008) Human sperm devoid of PLC, zeta 1 fail to induce Ca(2+) release and are unable to initiate the first step of embryo development. *J Clin Invest* 118: 3671-81

22nd Sep 2022

Dear Dr. Lin,

Thank you for the submission of your manuscript to EMBO Molecular Medicine. We have now received the enclosed reports from referees #2 and #3, and as you will see, they are supportive of publication. I am therefore pleased to inform you that we will be able to accept your manuscript once the following editorial points will be addressed:

1/ Manuscript text:
- Please accept the changes and only keep in track changes mode any new modification.
- Material and methods:
o Please indicate whether cells were authenticated (if applicable) and tested for mycoplasma contamination.
o Please indicate the origin of the mice.
- Data Availability section: Thank you for depositing the proteomics data in a public repository. Please also deposit your WES data in a public repository and update the checklist accordingly. The sentence "The source data [...] upon reasonable request" should be removed.

2/ Figures:
- Please provide exact p values where possible (instead of $p<0.0001$).
- Thank you for providing source data. We encourage you to also provide the original uncropped blots for figures 6b and EV2c.

3/ Thank you for providing The Paper Explained. I included minor changes, please let me know if you agree or amend as you see fit:
PROBLEM
Fertilization is the process by which male and female gametes are fused to initiate the development of a new organism. The total fertilization failure (TFF) is a common difficulty in assisted reproductive technology. Among couples with TFF, severe sperm head deformity is a frequent reason for male-source TFF. However, the genetic basis and molecular mechanisms of TFF caused by male factors remain to be clarified.
RESULTS
We identified two homozygous pathogenic variants of a novel gene IQ motif containing N (IQCN) by whole-exome sequencing of 42 males with TFF after ICSI. Abnormal acrosome structures were found in the spermatozoa of two affected individuals. We generated Iqcn (Iqcn-/-) knockout mice using CRISPR-Cas9 technology, which exhibited similar phenotypes as observed in humans. In vivo and in vitro studies confirmed that IQCN regulates microtubule nucleation during manchette assembly through calmodulin and calmodulin-related binding proteins. ICSI with assisted oocyte activation was successfully used to overcome genetic infertility. Our results suggest that IQCN plays an important role in spermatogenesis and male infertility by interacting with calmodulin to affect manchette assembly.
IMPACT
We provide direct evidence that homozygous variation of IQCN leads to abnormal acrosome structure and male infertility. These findings provide a new insight into the relationship between manchette assembly and fertilization during spermatogenesis and provide a target for the early diagnosis and treatment of male TFF. We also reveal that the IQCN-calmodulin complex regulates manchette assembly to affect the formation of the sperm head, resulting in defects in oocyte activation.

4/ Please replace "Additional information" by "For more information". The link http://exac.broadinstitute.org/ seems not to be working.

5/ Thank you for providing a synopsis. I added minor modifications, please let me know if you agree with the following or amend as you see fit:
Homozygous pathogenic variants in IQCN were identified in two male patients experiencing TFF. IQCN was found essential for regulating nucleation during microtubule assembly by interacting with calmodulin and calmodulin-related binding proteins.
- The two affected individuals showed increased proportion of sperm head malformations, and characteristics of a ruffled acrosome.
- Abnormal acrosome caused aberrant oocyte activation factor PLCζ localization and TFF after ICSI.
- The phenotype of Iqcn knockout (Iqcn-/-) male mice is similar to that of the affected individuals.
- IQCN interacts with calmodulin to promote prolonged manchette assembly in spermatids, and the disappearance of this interaction leads to nucleation defects.
- IQCN-related male infertility could be rescued by ICSI with assisted oocyte activation.

Thank you for providing a nice synopsis picture. Please resize it as a PNG/TIFF/JPEG file 550 px wide x 300-600 px high, and make sure that the text remains legible.

We note that you agree with the publication of the Review Process File (RPF) in conjunction with your manuscript. The Authors checklist will be published at the end of the RPF.

I look forward to receiving your revised manuscript.

With kind regards,

Lise Roth

Lise Roth, PhD
Senior Editor
EMBO Molecular Medicine

***** Reviewer's comments *****

Referee #2 (Remarks for Author):

The authors have adequately addressed all my comments in the revised manuscript, I have no more questions.

Referee #3 (Remarks for Author):

The authors did immunostaining of PLCz during spermatogenesis and showed that it localizes to the acroplaxome in the control and is lost in the Iqcn KO by the maturation phase of acrosome development. They also updated the ethics statement on using the calcium ionophore during IVF and included references to other papers who have used this technique for treating infertile patients. The reviewer doesn't have any other concerns about the paper.

The authors performed the requested editorial changes.

10th Oct 2022

Dear Dr. Lin,

I am pleased to inform you that your manuscript is now accepted for publication and will be sent to our publisher to be included in the next available issue of EMBO Molecular Medicine.

Please inform us as soon as the WES data are publicly available.

Congratulations on your interesting work!

With kind regards,

Lise Roth

Lise Roth, Ph.D
Senior Editor
EMBO Molecular Medicine

Follow us on Twitter @EmboMolMed
Sign up for eTOCs at embopress.org/alertsfeeds

EMBO Press Author Checklist

Corresponding Author Name:Ge Lin and Wei Zheng	
Journal Submitted to: EMBO Molecular Medicine	
Manuscript Number: EMM-2022-16501	

USEFUL LINKS FOR COMPLETING THIS FORM
The EMBO Journal - Author Guidelines
EMBO Reports - Author Guidelines
Molecular Systems Biology - Author Guidelines
EMBO Molecular Medicine - Author Guidelines

Reporting Checklist for Life Science Articles (updated January 2022)
This checklist is adapted from Materials Design Analysis Reporting (MDAR) Checklist for Authors. MDAR establishes a minimum set of requirements in transparent reporting in the life sciences (see Statement of Task: 10.31222/osf.io/9sm4x). Please follow the journal's guidelines in preparing your manuscript.
Please note that a copy of this checklist will be published alongside your article.

Abridged guidelines for figures
1. Data
The data shown in figures should satisfy the following conditions:
- the data were obtained and processed according to the field's best practice and are presented to reflect the results of the experiments in an accurate and unbiased manner.
- ideally, figure panels should include only measurements that are directly comparable to each other and obtained with the same assay.
- plots include clearly labeled error bars for independent experiments and sample sizes. Unless justified, error bars should not be shown for technical replicates.
- if n<5, the individual data points from each experiment should be plotted. Any statistical test employed should be justified.
- Source Data should be included to report the data underlying figures according to the guidelines set out in the authorship guidelines on Data Presentation.

2. Captions
Each figure caption should contain the following information, for each panel where they are relevant:
- a specification of the experimental system investigated (eg cell line, species name).
- the assay(s) and method(s) used to carry out the reported observations and measurements.
- an explicit mention of the biological and chemical entity(ies) that are being measured.
- an explicit mention of the biological and chemical entity(ies) that are altered/varied/perturbed in a controlled manner.
- the exact sample size (n) for each experimental group/condition, given as a number, not a range;
- a description of the sample collection allowing the reader to understand whether the samples represent technical or biological replicates (including how many animals, litters, cultures, etc.).
- a statement of how many times the experiment shown was independently replicated in the laboratory.
- definitions of statistical methods and measures:
  - common tests, such as t-test (please specify whether paired vs. unpaired), simple χ2 tests, Wilcoxon and Mann-Whitney tests, can be unambiguously identified by name only, but more complex techniques should be described in the methods section;
  - are tests one-sided or two-sided?
  - are there adjustments for multiple comparisons?
  - exact statistical test results, e.g., P values = x but not P values < x;
  - definition of 'center values' as median or average;
  - definition of error bars as s.d. or s.e.m.

Please complete ALL of the questions below.
Select "Not Applicable" only when the requested information is not relevant for your study.

Materials

Newly Created Materials	Information included in the manuscript?	In which section is the information available? (Reagents and Tools Table, Materials and Methods, Figures, Data Availability Section)
New materials and reagents need to be available; do any restrictions apply?	Not Applicable	

Antibodies	Information included in the manuscript?	In which section is the information available? (Reagents and Tools Table, Materials and Methods, Figures, Data Availability Section)
For **antibodies** provide the following information:<br>- Commercial antibodies: RRID (if possible) or supplier name, catalogue number and or/clone number<br>- Non-commercial: RRID or citation	Yes	Materials and Methods

DNA and RNA sequences	Information included in the manuscript?	In which section is the information available? (Reagents and Tools Table, Materials and Methods, Figures, Data Availability Section)
**Short novel DNA or RNA including primers, probes**: provide the sequences.	Yes	Appendix Table S3.

Cell materials	Information included in the manuscript?	In which section is the information available? (Reagents and Tools Table, Materials and Methods, Figures, Data Availability Section)
**Cell lines:** Provide species information, strain. Provide accession number in repository **OR** supplier name, catalog number, clone number, and/**OR** RRID.	Yes	Materials and Methods: HeLa cells (ATCC, Manassas, USA)
**Primary cultures:** Provide species, strain, sex of origin, genetic modification status.	Not Applicable	
Report if the cell lines were recently **authenticated** (e.g., by STR profiling) and tested for mycoplasma contamination.	Yes	

Experimental animals	Information included in the manuscript?	In which section is the information available? (Reagents and Tools Table, Materials and Methods, Figures, Data Availability Section)
**Laboratory animals or Model organisms:** Provide species, strain, sex, age, genetic modification status. Provide accession number in repository **OR** supplier name, catalog number, clone number, **OR** RRID.	Yes	Materials and Methods
**Animal observed in or captured from the field:** Provide species, sex, and age where possible.	Not Applicable	
Please detail **housing and husbandry conditions**.	Yes	Materials and Methods

Plants and microbes	Information included in the manuscript?	In which section is the information available? (Reagents and Tools Table, Materials and Methods, Figures, Data Availability Section)
**Plants:** provide species and strain, ecotype and cultivar where relevant, unique accession number if available, and source (including location for collected wild specimens).	Not Applicable	
**Microbes:** provide species and strain, unique accession number if available, and source.	Not Applicable	

Human research participants	Information included in the manuscript?	In which section is the information available? (Reagents and Tools Table, Materials and Methods, Figures, Data Availability Section)
If collected and within the bounds of privacy constraints report on age, sex and gender or ethnicity for all study participants.	Yes	Results

Core facilities	Information included in the manuscript?	In which section is the information available? (Reagents and Tools Table, Materials and Methods, Figures, Data Availability Section)
If your work benefited from core facilities, was their service mentioned in the acknowledgments section?	Not Applicable	

Design

Study protocol	Information included in the manuscript?	In which section is the information available? (Reagents and Tools Table, Materials and Methods, Figures, Data Availability Section)
If study protocol has been **pre-registered, provide DOI in the manuscript**. For clinical trials, provide the trial registration number **OR** cite DOI.	Not Applicable	
Report the **clinical trial registration number** (at ClinicalTrials.gov or equivalent), where applicable.	Not Applicable	

Laboratory protocol	Information included in the manuscript?	In which section is the information available? (Reagents and Tools Table, Materials and Methods, Figures, Data Availability Section)
Provide DOI OR other citation details if **external detailed step-by-step protocols** are available.	Not Applicable	

Experimental study design and statistics	Information included in the manuscript?	In which section is the information available? (Reagents and Tools Table, Materials and Methods, Figures, Data Availability Section)
Include a statement about **sample size** estimate even if no statistical methods were used.	Yes	Materials and Methods
Were any steps taken to minimize the effects of subjective bias when allocating animals/samples to treatment (e.g. **randomization procedure**)? If yes, have they been described?	Not Applicable	
Include a statement about **blinding** even if no blinding was done.	Not Applicable	
Describe **inclusion/exclusion criteria** if samples or animals were excluded from the analysis. Were the criteria pre-established?<br><br>If sample or data points were omitted from analysis, report if this was due to attrition or intentional exclusion and provide justification.	Yes	Materials and Methods
For every figure, are **statistical tests** justified as appropriate? Do the data meet the assumptions of the tests (e.g., normal distribution)? Describe any methods used to assess it. Is there an estimate of variation within each group of data? Is the variance similar between the groups that are being statistically compared?	Yes	Materials and Methods, Figure legends

Sample definition and in-laboratory replication	Information included in the manuscript?	In which section is the information available? (Reagents and Tools Table, Materials and Methods, Figures, Data Availability Section)
In the figure legends: state number of times the experiment was **replicated** in laboratory.	Yes	Figure legends
In the figure legends: define whether data describe **technical or biological replicates**.	Yes	Figure legends

Ethics

Ethics	Information included in the manuscript?	In which section is the information available? (Reagents and Tools Table, Materials and Methods, Figures, Data Availability Section)
Studies involving **human participants**: State details of **authority granting ethics approval** (IRB or equivalent committee(s), provide reference number for approval.	Yes	Materials and Methods
Studies involving **human participants**: Include a statement confirming that **informed consent** was obtained from all subjects and that the experiments conformed to the principles set out in the WMA Declaration of Helsinki and the Department of Health and Human Services Belmont Report.	Yes	Materials and Methods
Studies involving **human participants:** For publication of **patient photos**, include a statement confirming that consent to publish was obtained.	Not Applicable	
Studies involving experimental **animals**: State details of **authority granting ethics approval** (IRB or equivalent committee(s), provide reference number for approval. Include a statement of compliance with ethical regulations.	Yes	Materials and Methods
Studies involving **specimen and field samples:** State if relevant **permits** obtained, provide details of authority approving study; if none were required, explain why.	Not Applicable	

Dual Use Research of Concern (DURC)	Information included in the manuscript?	In which section is the information available? (Reagents and Tools Table, Materials and Methods, Figures, Data Availability Section)
Could your study fall under dual use research restrictions? Please check biosecurity documents and list of **select agents and toxins** (CDC): https://www.selectagents.gov/sat/list.htm	Not Applicable	
If you used a select agent, is the security level of the lab appropriate and reported in the manuscript?	Not Applicable	
If a study is subject to dual use research of concern regulations, is the name of the **authority granting approval and reference number** for the regulatory approval provided in the manuscript?	Not Applicable	

Reporting

The MDAR framework recommends adoption of discipline-specific guidelines, established and endorsed through community initiatives. Journals have their own policy about requiring specific guidelines and recommendations to complement MDAR.

Adherence to community standards	Information included in the manuscript?	In which section is the information available? (Reagents and Tools Table, Materials and Methods, Figures, Data Availability Section)
State if relevant guidelines or checklists (e.g., **ICMJE, MIBBI, ARRIVE, PRISMA**) have been followed or provided.	Not Applicable	
For **tumor marker prognostic studies,** we recommend that you follow the **REMARK** reporting guidelines (see link list at top right). See author guidelines, under 'Reporting Guidelines'. Please confirm you have followed these guidelines.	Not Applicable	
For **phase II and III randomized controlled trials**, please refer to the **CONSORT** flow diagram (see link list at top right) and submit the CONSORT checklist (see link list at top right) with your submission. See author guidelines, under 'Reporting Guidelines'. Please confirm you have submitted this list.	Not Applicable	

Data Availability

Data availability	Information included in the manuscript?	In which section is the information available? (Reagents and Tools Table, Materials and Methods, Figures, Data Availability Section)
Have **primary datasets** been deposited according to the journal's guidelines (see 'Data Deposition' section) and the respective accession numbers provided in the Data Availability Section?	Yes	Data availability
Were **human clinical and genomic datasets** deposited in a public access-controlled repository in accordance to ethical obligations to the patients and to the applicable consent agreement?	Not Applicable	
Are **computational models** that are central and integral to a study available without restrictions in a machine-readable form? Were the relevant accession numbers or links provided?	Not Applicable	
If publicly available data were reused, provide the respective **data citations in the reference list**.	Not Applicable	
