# Supplementary material for: IQCN disruption causes fertilization failure and male infertility due to manchette assembly defect
Source: EMBO Mol Med. 2022 Nov 2;14(12):e16501. doi: 10.15252/emmm.202216501 (PMC9728048; doi:10.15252/emmm.202216501)

Figure EV2c

1) IP:CaM: Anti-IFT74

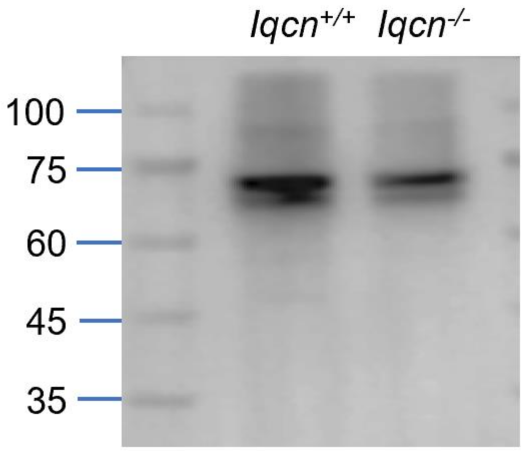

2) IP:CaM: Anti-IFT81

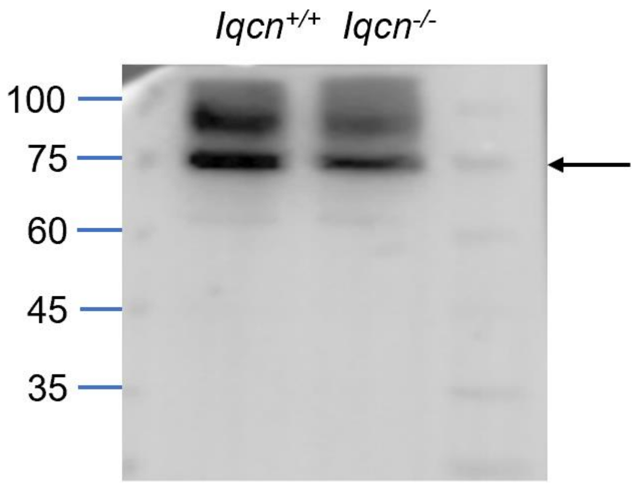

3) Input: Anti-IFT74

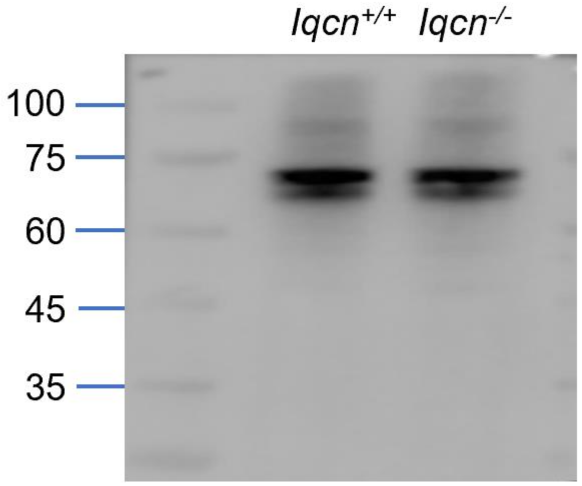

4）Input: Anti-IFT81

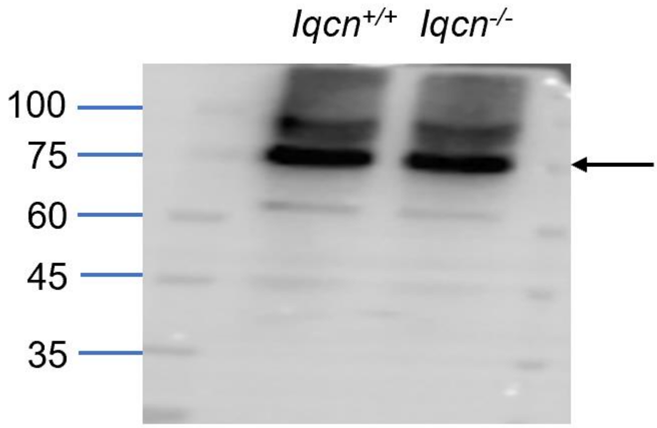

5）Input: Anti-GAPDH

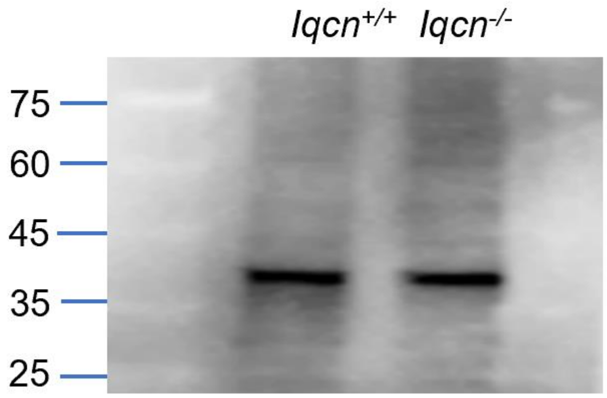

Supplement: Supplementary file 3 — Source Data for Expanded View [file EMMM-14-e16501-s009.zip › emmm202216501-sup-0003-SDataEV/EMM-2022-16501-V3-Figure_EV2_Source_Data-sd.pdf.zip!/EMM-2022-16501-V3-Figure_EV2_Source_Data-sd.pdf]
